# Application of Quinoline Ring in Structural Modification of Natural Products

**DOI:** 10.3390/molecules28186478

**Published:** 2023-09-06

**Authors:** Yu-Qing Zhao, Xiaoting Li, Hong-Yan Guo, Qing-Kun Shen, Zhe-Shan Quan, Tian Luan

**Affiliations:** 1 Key Laboratory of Natural Medicines of the Changbai Mountain, Ministry of Education, College of Pharmacy, Yanbian University, Yanji 133002, China; 2021010881@ybu.edu.cn (Y.-Q.Z.); 0000008735@ybu.edu.cn (X.L.); hongyanguo@ybu.edu.cn (H.-Y.G.); qkshen@ybu.edu.cn (Q.-K.S.); 2 Department of Pharmacy, Shenyang Medical College, Shenyang 110034, China

**Keywords:** natural products, quinoline, pharmacological activities

## Abstract

Natural compounds are rich in pharmacological properties that are a hot topic in pharmaceutical research. The quinoline ring plays important roles in many biological processes in heterocycles. Many pharmacological compounds, including saquinavir and chloroquine, have been marketed as quinoline molecules with good anti-viral and anti-parasitic properties. Therefore, in this review, we summarize the medicinal chemistry of quinoline-modified natural product quinoline derivatives that were developed by several research teams in the past 10 years and find that these compounds have inhibitory effects on bacteria, viruses, parasites, inflammation, cancer, Alzheimer’s disease, and others.

## 1. Introduction

Active molecules of natural products have always been an important source of drug leads due to their diverse chemical structures and extensive pharmacological activities. According to statistics, from January 1981 to September 2019, the FDA has approved 1881 new drugs, of which about half are directly or indirectly derived from natural compounds [1]. Quinoline, also known as benzo[*b*]pyridine, is a nitrogen-containing heterocyclic aromatic molecule with a weak tertiary base that can form salts with acids and perform electrophilic substitution reactions and reactions resembling those of pyridine and benzene. Quinolines have numerous biological effects, such as antibacterial [2,3,4], antifungal [5], antituberculosis [6], antiprotozoal [7,8], antineoplastic [9], anti-viral [10], anti-cholesterol medications [11], analgesics [12], anti-disease Alzheimer’s pharmaceuticals [13], and more.

**Figure 1:**
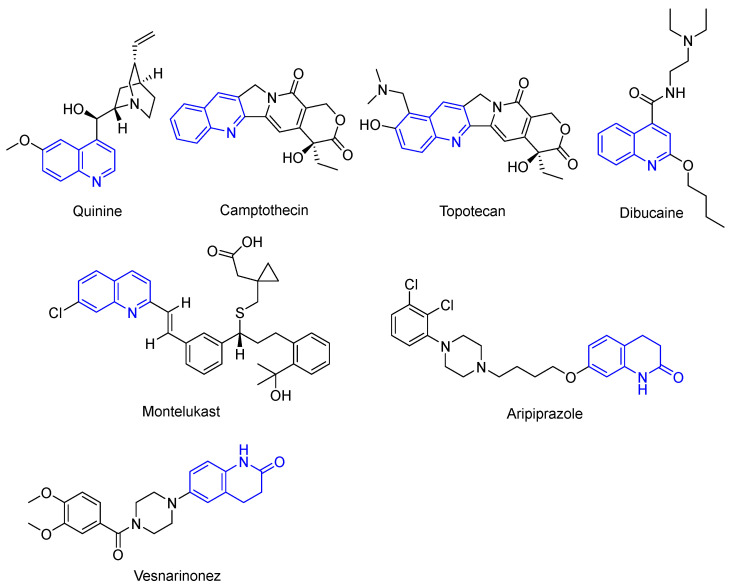
The chemical structures of introduction.

In fact, quinoline drugs are widely used in the pharmaceutical industry. Many drugs contain quinoline rings. For example, quinine (Figure 1) is one of the natural products is present in the bark of cinchona, which has been used to treat malaria. Camptothecin (Figure 1) is a quinoline alkaloid extracted from Camptotheca acuminata, and its analogue, topotecan (Figure 1), are effective antitumor drugs. And other chemical drugs, such as dibucaine (Figure 1) as a local anesthetic, montelukast (Figure 1) for asthma, aripiprazole (Figure 1) as an antipsychotic, vesnarinonez (Figure 1) as a cardiac agent, etc., all contain quinoline structure. Creating novel homologs with enhanced biological activity and fewer potentially harmful side effects has been a goal for many years. Emphasizing the biological activities of the quinolones, we will highlight some recent results regarding the development of novel quinoline-natural product hybrids in this study.

## 2. Method

Published articles, network databases (PubMed, Science Direct, SCI Finder, CNKI), and clinical trial websites https://clinicaltrials.gov/ (accessed on 31 September 2022) related to natural products and quinoline derivatives) were included in the discussion. The 94 studies that met the inclusion criteria were chosen for discussion. It is possible to categorize natural quinoline derivative compounds according to their biological activity by screening. The entire text is divided into sections based on how biologically active their derivatives are, namely, in the following categories: inhibition of bacteria, viruses, parasites, inflammation, cancer, Alzheimer’s disease, etc. The biological activities of quinoline derivatives were discussed in detail. It is worth noting that most derivatives exhibit enhanced biological activity and reduced cytotoxicity compared to lead compounds. Quinoline has many advantages and can be widely used in the synthesis of natural product derivatives to enhance the properties of drug bulks.

## 3. Pharmacological Activities

### 3.1. Anti-Alzheimer’s Disease Activity

**Figure 2:**
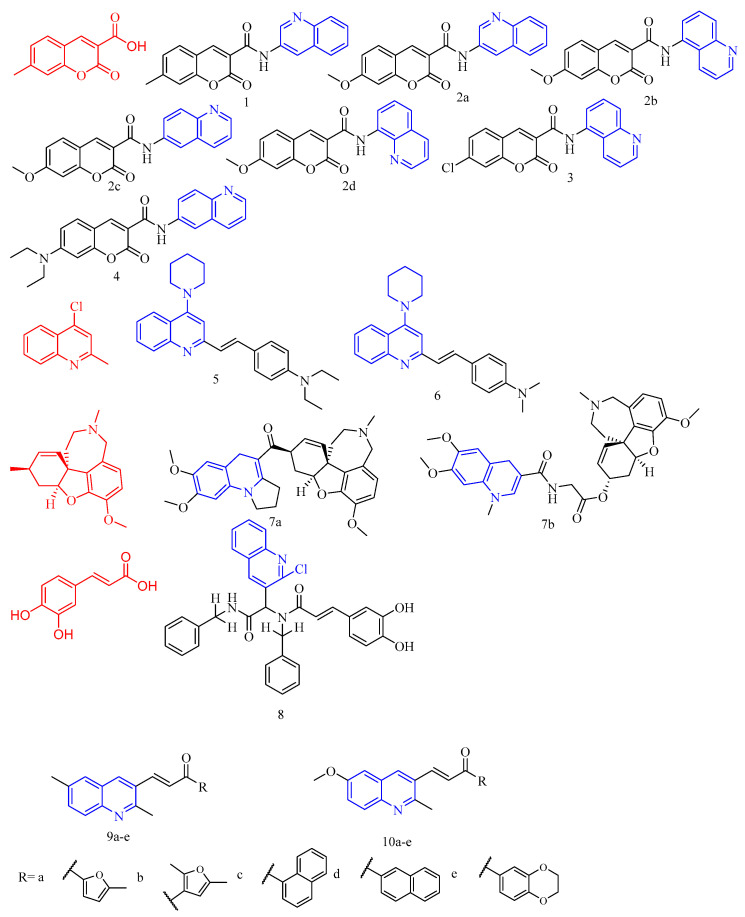
The chemical structures of anti-Alzheimer’s disease compounds **1**–**10**.

Coumarin (chemical formula (CF): C_9_H_6_O_2_, molecular weight (MW): 146.14, 2*H*-1-benzopyran-2-one) is a phenolic compound widely found in orchids, legumes, Umbelliferae, Compositae, Rutaceae*,* and other plants. Duarte and colleagues [14] synthesized quinoline-substituted compounds **1**–**4** (Figure 2) (Table 1) at the seventh position of coumarin and determined their AChE/BChE activity regulation. Compound **2a**, containing a strong electron-donating group (methoxy) at position 7, were against both enzymes (AChE IC_50_ = 194 μM and BChE IC_50_ = 255 μM), being the only representative dual compound of the entire series. However, compound **2b**, which has the same substitution pattern at position 7 and a different aminoquinoline at position 3, showed selective activity against the AChE enzyme (AChE IC_50_ = 181 μM, selectivity ratio > 2.75). Compound **3**, with the same amidoquinoline as compound **2b** at position 3 and an electron-withdrawing atom (chloride atom) at position 7 of the coumarin scaffold, was the only selective BChE inhibitor of the entire series (BChE IC_50_ = 146 μM, selectivity ratio < 0.29). The most active and selective AChE compound among all others was compound **4**, which had an electron-donating group (diethylamine) at position 7 and a 6-quinoline derivative at position 3 (AChE IC_50_ = 159 μM, selectivity ratio > 3.13). This result was intriguing because other electron-donating groups such as methyl and methoxy groups, compounds **1** and **2c**, respectively, present at position 7, showed results that were the opposite of this trend, suggesting that the diethylamine substituents at position 7 are only marginally important for the AChE binding affinity. Compound **4** is also an iron chelator (100 μM Fe chelation = 72.87%), forming a well-defined stacking contact with Phe330 and interacting with Tyr121 residues via hydrogen bonding.

Wang and colleagues [15] designed and synthesized 2-arylethenylquinoline derivatives **5** and **6** (Figure 1) (Table 1) against AChE and BChE. The results showed that compounds **5** and **6** had moderate ChE inhibitory activity (IC_50_ of compound **5** AChE and BChE were 64.0 ± 0.1 and 0.2 ± 0.1 μM, respectively; and the IC_50_ of compound **6** AChE and BChE were 68.3 ± 0.1 and 1.0 ± 0.1 μM, respectively). These two compounds showed high selectivity for BChE; however, their inhibitory activity against ChE was significantly weaker than that of positive control tacrine. Even the most energetic compound **5** was 640- and 7-fold weaker than tacrine against AChE and BChE, respectively.

Galantamine (CF: C_17_H_22_ClNO_3_, MW:323.8145, (4a*S*,6*R*,8a*S*)-4a,5,9,10,11,12-Hexahydro-3-methoxy-11-methyl-6*H*-benzofuro[3a,3,2-ef][2]benzazepin-6-ol) was initially isolated and extracted from the bulbs of snowdrops. However, due to the scarcity of the extraction species and the high cost of the extraction, numerous businesses throughout the world started producing galantamine by chemical synthesis. Ţînţaş and colleagues [16] synthesized dihydroquinoline galanthamine derivatives **7a**–**b** (Figure 2) (Table 1) and evaluated their in vitro inhibitory activity on AChE in the human body. Compound **7a** could not be assessed since it was insoluble in water; however, compound **7b** had an IC_50_ larger than 10 μM. Additionally, its activity was significantly lower than that of the parent galanthamine.

Caffeic acid (CA, CF: C_9_H_8_O_4_, MW: 180.15, 3-(3,4-dihydroxyphenyl)- (9CI, ACI)) is derived from the whole plant of Solidago virga-urea L.var.leiocarpa, the fruit of *Crataegus pinnatifida Bge.*, and others. Benchekroun and colleagues [17] synthesized a caffeic acid derivative **8** (Figure 2) (Table 1) and tested its antioxidant capacity. The experimental findings showed compound **8** showed 25% or more significant neuroprotection against H_2_O_2_ damage at 10 μM.

Chromones (CF: C_9_H_6_O_2_, MW: 146.1427, 2,3-Benzo-4-pyrone), scientific name benzo-γ-pyrone, are widely present in plants, and some are colored substances, so their matrixes are called chromone. Shah and colleagues [18] synthesized chromone quinoline derivatives **9a**–**e** and **10a**–**e** (Figure 2) (Table 1) and evaluated their cholinesterase inhibitory activity. The screening of quinolinyl-chromone derivatives **9a**–**e** and **10a**–**e** for ChE revealed that most are selective BChE inhibitors. The active group connected to the quinoline acyl chalcone derivative has significant AChE inhibitory activity. The most effective molecule against BChE among 2,6-dimethyl quinoline derivatives is **9e** (2,3-dihydrobenzo[b]1,4-dioxin-6-yl as Ar substituent) with an IC_50_ of 0.56 ± 0.02 μM. The exceptional inhibitory potential of compound **9e** may be attributed to the presence of highly electronegative oxygen atoms (1,4-dioxane) and a tiny functional group (-CH_3_). The chemical **10e** likewise contains 1,4-dioxane, but it has a methoxy (-OCH_3_) functional group in its basic structure, which may limit its inhibitory effectiveness against BChE. Compounds **10a** and **10b** inhibit BChE significantly, with IC_50_ of 0.94 ± 0.04 μM and 0.73 ± 0.03 μM, respectively. These compounds have 5-methylfuran-2-yl (for **10a**) and 2,5-dimethylfuran-3-yl (for **10b**) substitutions at the -Ar position of the quinolinyl ring. When the experimental data from both series were examined, a modest decrease in the inhibitory potential was observed in the presence of a 6-methoxy group at the quinolinyl moiety. To investigate the putative mechanism of ChEs inhibition, detailed kinetic investigations of the most effective derivatives were conducted. The results indicated that compound **9e** could interact with both the catalytic anionic site (CAS) and the peripheral anionic site (PAS) of BChE.

**Table 1:** Quinoline derivatives with anti-Alzheimer’s disease activity.

Compd.	Activity	Target	Origin	Ref
**1**	AChE IC_50_ = 324.88 μM	AChE	synthetic	[14]
**2a**	AChE IC_50_ = 194 μM BChE IC_50_ = 255 μM	ACHE BCHE	synthetic	[14]
**2b**	AChE IC_50_ = 181.72 μM, selectivity ratio > 2.75	ACHE	synthetic	[14]
**2c**	AChE IC_50_ > 500 μM BChE IC_50_ > 500 μM	-	synthetic	[14]
**2d**	AChE IC_50_ > 500 μM BChE IC_50_ > 500 μM	-	synthetic	[14]
**3**	BChE IC_50_ = 146.74 μM, selectivity ratio < 0.29	BCHE	synthetic	[14]
**4**	AChE IC_50_ = 159.53 μM, selectivity ratio > 3.13	ACHE	synthetic	[14]
**5**	AChE IC_50_ = 64.0 μM BChE IC_50_ = 0.2 μM	ACHE BCHE	synthetic	[15]
**6**	AChE IC_50_ = 68.3 μM BChE IC_50_ = 1.0 μM	ACHE BCHE	synthetic	[15]
**7a**	-	-	synthetic	[16]
**7b**	AChE IC_50_ > 10 μM	ACHE	synthetic	[16]
**8**	25% H_2_O_2_ damage at 10 μM.	-	synthetic	[17]
**9a**	AChE IC_50_ = 2.99 μM BChE IC_50_ = 0.91 μM	ACHE BCHE	synthetic	[18]
**9b**	AChE IC_50_ = 0.32 μM BChE IC_50_ = 0.90 μM	ACHE BCHE	synthetic	[18]
**9c**	AChE IC_50_ = 3.99 μM BChE IC_50_ = 0.64 μM	ACHE BCHE	synthetic	[18]
**9d**	AChE IC_50_ = 3.45 μM BChE IC_50_ = 0.63 μM	ACHE BCHE	synthetic	[18]
**9e**	AChE IC_50_ = 44.46 μM BChE IC_50_ = 0.56 μM	ACHE BCHE	synthetic	[18]
**10a**	AChE IC_50_ = 43.36 μM BChE IC_50_ = 0.94 μM	ACHE BCHE	synthetic	[18]
**10b**	AChE IC_50_ = 46.42 μM BChE IC_50_ = 0.73 μM	ACHE BCHE	synthetic	[18]
**10c**	AChE IC_50_ = 3.91 μM BChE IC_50_ = 2.15 μM	ACHE BCHE	synthetic	[18]
**10d**	AChE IC_50_ = 3.36 μM BChE IC_50_ = 2.36 μM	ACHE BCHE	synthetic	[18]
**10e**	AChE IC_50_ = 40.29 μM BChE IC_50_ = 1.32 μM	ACHE BCHE	synthetic	[18]

Summary: Coumarin, 2-aryl, and Chromones-linked quinoline derivatives can enhance their AChE/BChE activity. Among them, compounds **9b** and **9e** showed inhibitory effects on AchE and BchE, with IC_50_ values of 0.32 and 0.56 μM, respectively. When the 6 position of Chromones is methyl, the activity is significantly higher than that of methoxy; the activity is most obvious when the R group is furan. In addition, coumarin–quinoline derivatives **3** and **4** showed inhibitory effects on AchE and BchE, with IC_50_ values of 146.74 and 153.53 μM, respectively; therefore, the Chromones-linked quinoline derivatives have the value of further research.

### 3.2. Anti-Osteoporosis Activity

**Figure 3:**
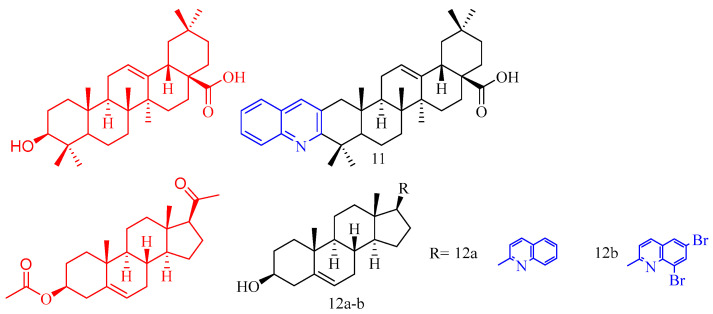
The chemical structures of anti-osteoporosis compounds **11**–**12**.

Oleanolic acid (OA, CF: C_30_H_48_O_3_, MW: 456.70, (3β)-3-Hydroxyolean-12-en-28-oic acid) is a kind of pentacyclic triterpenoid obtained by separating and extracting from the fruit of the whole herb of the gentian family, the genus of the genus Radix, or Ligustrum lucidum. It exists in free form and glycoside in polyphenols in plants. Li and colleagues [19] tested the inhibitory activity of OA derivatives **11** (Figure 3) (Table 2) in the formation of TRAP-positive, osteoclast-like multinucleated cells (OCLs) induced by the effect of 1a, 25-dihydroxy vitamin D_3_ [1a,25(OH)_2_D_3_] effect. The results showed that compound **11** exhibited good activity at 20 μM (OCL% = 73.0%), which was better than OA at 20 μM. Encouragingly, compound **11** showed moderate activity even at 2 μM (OCL% = 18.9%).

Pregnenolone (CF: C_21_H_32_O_2_, MW: 316.48, 3β-Hydroxy-5-pregnen-20-one), a naturally occurring endogenous steroid, is well-known as one of the biosynthetic precursors of steroid hormones. Maurya and colleagues [20] synthesized pregnenolone derivatives **12a**–**b** (Figure 3) (Table 2) and tested its osteogenic effect. Compared with untreated control cells, compounds **12a** and **12b** significantly increased ALP activity. Among the compounds studied, compound **12a** showed the greatest osteogenic effect according to the ALP activity evaluation. At the concentration of 1 pM and 100 pM the ALP activity increased by 100% and 85%, respectively. In addition, studies have shown that compound **12a** can significantly increase the formation of mineral nodules, and compound **12a** upregulates the expression of osteogenic markers BMP-2, RUNX-2, and OCN.

**Table 2:** Quinoline derivatives with anti-osteoporosis activity.

Compd.	Activity	Target	Origin	Ref
**11**	2 μM OCL% = 18.9% 20 μM OCL% = 73.0%	-	synthetic	[19]
**12a**	1 pM ALP% = 100% 100 pM ALP% = 85%	ALP, BMP-2, RUNX-2, OCN	synthetic	[20]
**12b**	1 pM ALP% = 70% 100 pM ALP% = 60%	ALP	synthetic	[20]

Summary: The introduction of quinoline by oleanolic acid only showed moderate activity. The introduction of quinoline by pregnenolone still showed strong ALP activity at a concentration of 1 pM, and there was some research on its mechanism. The anti-osteoporosis activity of compound **12a** has a certain research value.

### 3.3. Anti-Viral Activity

**Figure 4:**
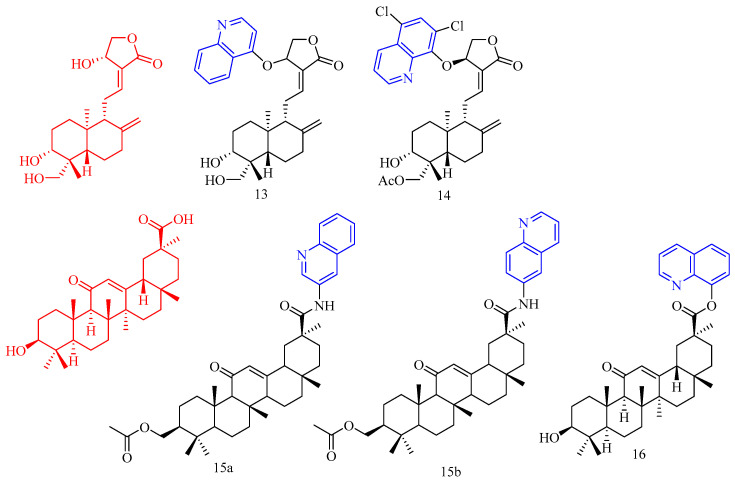
The chemical structures of anti-viral compounds **13**–**16**.

Andrographolide (CF: C_20_H_30_O_5_, MW: 350.45, 3-[2-[Decahydro-6-hydroyx-5-(hydroxymethyl)-5,8a-dimethyl-2-methylene-1-naphthalenylethylidene]dihydro-4-hydroxy-2(3*H*)-furanone), is derived from the leaves of Andrographis paniculata. Li and colleagues [21] synthesized andrographolide quinoline derivative **13**–**14** (Figure 4) (Table 3) and tested their anti-Zika virus activity. The results show that the EC_50_ of compound **13** is 1.3 μM. SI > 16 (CC_50_ values of SNB-19 and Vero cell lines are 22.7 and 20.9 μM). EC_50_ of compound **14** is 4.5 μM. SI > 19 (CC_50_s of SNB-19 and Vero cell lines are 88.7 and 85.0 μM). In conclusion, compounds **13** and **14** have good anti-ZIKV virus activity.

Baltina and colleagues [22] tested the anti-ZIKV virus activity of glycyrrhizic acid derivatives **15a**–**b** (Figure 4) (Table 3) and found that these two compounds **15a**–**b** did not have good anti-Zika virus activity, the IC_50_ value of compound **15a** is less than 30 μM.

Wang and colleagues [23] tested the glycyrrhetinic acid quinoline derivative **16** (Figure 4) (Table 3) and tested its anti-HBV activity. Compound **16** inhibits HBV DNA replication with IC_50_ of 15.30 μM (SI > 111.0), showing strong anti-HBV activity.

**Table 3:** Quinoline derivatives with anti-viral activity.

Compd.	Activity	Target	Origin	Ref
**13**	anti-Zika virus EC_50_ = 1.3 μM	-	synthetic	[21]
**14**	anti-Zika virus EC_50_ = 4.5 μM	-	synthetic	[21]
**15a**	anti-Zika virus IC_50_ < 30 μM	ZIKV NS2B-NS3 protease	synthetic	[22]
**15b**	anti-Zika virus IC_50_ < 30 μM	ZIKV NS2B-NS3 protease	synthetic	[22]
**16**	anti-HBV IC_50_ = 15.30 μM	-	synthetic	[23]

### 3.4. Anti-Hyperglycemic Activity

**Figure 5:**
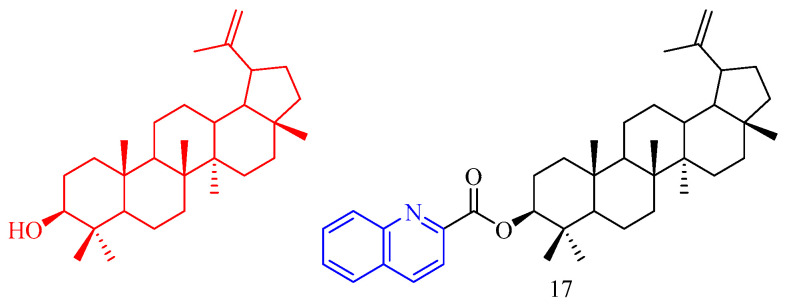
The chemical structures of anti-hyperglycemic compound **17**.

Lupeol (CF: C_30_H_50_O, MW: 426.7174, (3β)-Lup-20(29)-en-3-ol) is a compound found in the epidermis of lupin seeds, fig latex, and rubber. Reddy and colleagues [24] synthesized lupeol quinoline derivative **17** (Figure 5) (Table 4) and measured its anti-lipid activity. The hypolipidemic activity of derivative **17** was screened in mice at a dose of 50 mg/kg body weight. Blood cholesterol decreased by 27% (*p* < 0.05). Compound **17** showed good cholesterol-lowering effectiveness. Unfortunately, compound **17** reduces HDL-C.

**Table 4:** Quinoline derivatives with anti-hyperglycemic activity.

Compd.	Activity	Target	Origin	Ref
**17**	Blood cholesterol decreased 27%	triglycerides	synthetic	[24]

### 3.5. Anti-Inflammatory Activity

**Figure 6:**
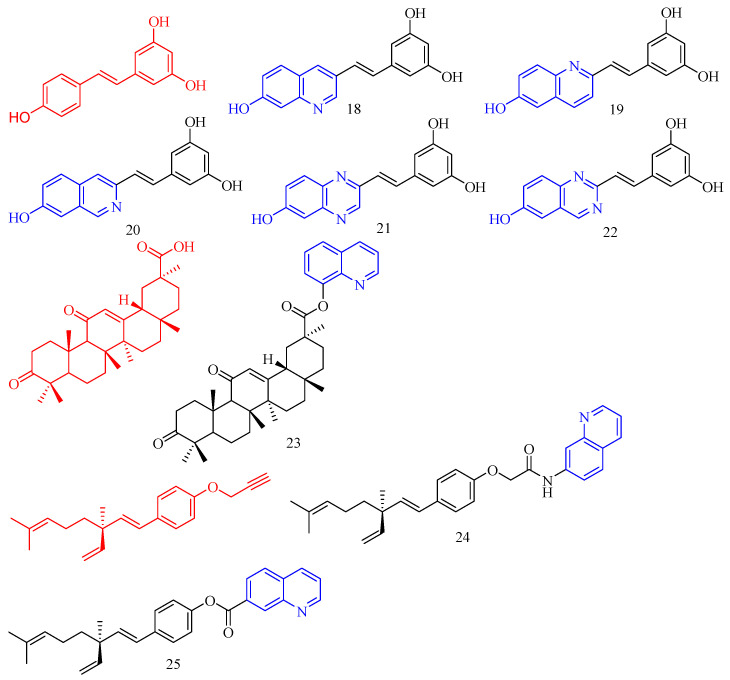
The chemical structures of anti-inflammatory compounds **18**–**25**.

Nam and colleagues [25] synthesized a series of resveratrol derivatives **18**–**22** (Figure 6) (Table 5) and measured their anti-inflammatory activities. The iEI value of compounds **18**–**22** is higher than that of the parent compound (iEI values of compounds **18**–**22** are 3.75, 3.18, 4.84, 2.11, and 4.27, respectively).

Glycyrrhetinic acid (GA, CF: C_30_H_46_O_4_, MW: 470.69, 3*β*-hydroxy-11-oxo-18*βH*-Olean-12-en-30-oic acid) is a well-known pentacyclic triterpene extracted from liquorice root. Bian and colleagues [26] synthesized quinoline glycyrrhetinic acid derivative **23** (Figure 6) (Table 5) and measured its cytotoxicity and anti-inflammatory activities. Because of its low cytotoxicity, compound **23** was chosen for further investigation of anti-inflammatory effects. The inhibitory effect of quinoline compound **23** in glycyrrhetinic acid structure on IL-6 was significantly stronger than that of glycyrrhetinic acid.

Bakuchiol (CF: C_18_H_24_O, MW: 256.383, 4-(3,7-Dimethyl-3-vinylocta-1,6-dien-1-yl)phenol) is a natural plant component found in plant seeds. Ma and colleagues [27] synthesized quinoline bakuchiol derivatives **24**–**25** (Figure 6) (Table 5) and evaluated their anti-inflammatory activity in vitro. Adding quinoline structure did not increase BAK activity but considerably lowered its toxicity. Compound **24** had a moderate inhibitory effect on NO, but compound **25** had a strong inhibitory effect. Compounds **24**–**25** slightly inhibited IL-6 (*p* < 0.05 vs. LPS group) but did not affect TNF-a.

**Table 5:** Quinoline derivatives with anti-inflammatory activity.

Compd.	Activity	Target	Origin	Ref
**18**	iEI = 3.75	IL-1β, IL-6	synthetic	[25]
**19**	iEI = 3.18	IL-1β, IL-6	synthetic	[25]
**20**	iEI = 4.84	IL-1β, IL-6	synthetic	[25]
**21**	iEI = 2.11	IL-1β, IL-6	synthetic	[25]
**22**	iEI = 4.27	IL-1β, IL-6	synthetic	[25]
**23**	IL-6 stronger than that of glycyrrhetinic acid.	IL-6, TNF-α, NO, iNOS, COX-2	synthetic	[26]
**24**	moderate inhibitory effect on NO	Erythroid 2-related factor 2, Heme oxygenase-1	synthetic	[27]
**25**	strong inhibitory effect on NO	Erythroid 2-related factor 2, Heme oxygenase-1	synthetic	[27]

### 3.6. Antithrombotic Activity

**Figure 7:**
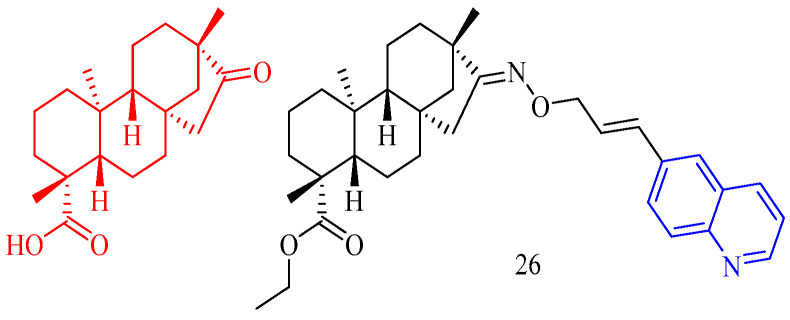
The chemical structures of antithrombotic compound **26**.

Isosteviol (CF: C_20_H_30_O_3_, MW: 318.4504, (4*α*,8*β*,13*β*)-13-Methyl-16-oxo-17-norkauran-18-oic acid) has the structural characteristics of tetracyclic diterpenoids. Chen and colleagues [28] synthesized an isosteviol–quinoline derivative **26** (Figure 7) (Table 6) and evaluated it in vitro FXa inhibition. The modification impact may not be optimum due to the Ki value of **26** on FXa being 4.177 μM, which is lower than the positive control for antithrombotic activity (K_i_ = 3.4 nM).

**Table 6:** Quinoline derivatives with antithrombotic activity.

Compd.	Activity	Target	Origin	Ref
**26**	antithrombotic Ki = 3.4 nM	FXa	synthetic	[28]

### 3.7. Anti-Parasitic Activity

**Figure 8:**
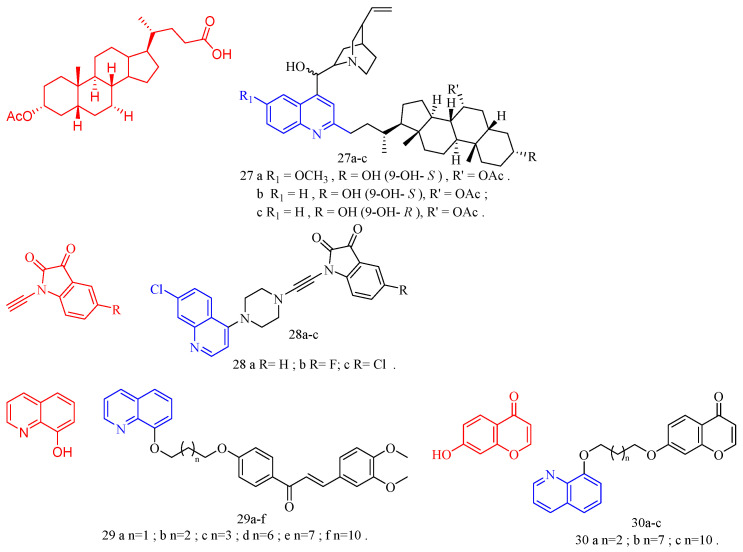
The chemical structures of anti-parasitic compounds **27**–**30**.

Cinchonasine (CF: C_20_H_24_N_2_O_2_, MW: 324.417, (8*S*,9*R*)-6′-Methoxy-cinchona-9-ol sulfate dihydrate) is the main alkaloid in the bark of the cinchona tree and its congeners. The structure of cinchonasine includes a quinoline ring. Leverrier and his colleagues [29] evaluated the anti-parasitic activity of cinchona–alkaloid derivatives **27a**–**c** (Figure 8) (Table 7). According to the results, compounds **27a**–**c** had good anti-*T. brucei* activity (IC_50_s of compound **27a**–**c** are 0.37, 0.39, and 0.40 μg/mL, respectively), and good resistance to anti-*L. mexicana* activity (IC_50_s of compound **27a**–**c** are 3.86, 3.39, and 3.45 μg/mL, respectively).

Isatin (CF: C_8_H_5_NO_2_, MW: 147.13, 2,3-Indolinedione) is an orange-red single co-prism crystal. Nisha and his colleagues [30] synthesized the isatin–quinoline derivatives **28a**–**c** (Figure 8) (Table 7) and determined their anti-trichomonas activity. Compounds **28a**–**c** among them have effective anti-trichomonas action. At 50 μM, the growth inhibition of **28a**–**c** was 98%, 100%, and 100%, respectively. **28a** and **28c** have IC_50_s of 22.2 and 11.3 μM, respectively. **28a**–**28c** had no cytotoxicity to PC-3 cells.

Licorice chalcone A (CF: C_21_H_22_O_4_, MW: 338.4, ((2*E*)-3-[5-(1,1-dimethylprop-2-en-1-yl)-4-hydroxy-2-methoxyphenyl]-1-(4-hydroxyphenyl)prop-2-en-1-one)) is derived from the roots of *Glycyrrhiza glabra* and *G. inflata*. Coa and colleagues [31] synthesized quinoline–chalcone derivatives **29a**–**f** and quinoline–chromone derivatives **30a**–**c** (Figure 8) (Table 7), and evaluated their activity against *Leishmania* (*Viannia*) *panamensis*. Compounds **29a**–**f** and **30a**–**c** demonstrated activity against *Leishmania* (*V*) *panamensis*, while compounds **29b**–**e**, and **30a**–**b** demonstrated activity against *Trypanosoma cruzi* with EC_50_ values less than 18 μg/mL. Compound **29f** was the most active compound against *Leishmania* (*V*) *panamensis* and *Trypanosoma cruzi*, with EC_50_s of 6.11 ± 0.26 and 4.09 ± 0.24 μg/mL. All hybrid compounds outperformed the anti-leishmanial drug meglumine antimoniate. Compounds **29d** and **30b** outperformed benznidazole, the current anti-trypanosomal drug; however, these compounds were toxic to mammalian U-937 cells.

**Figure 9:**
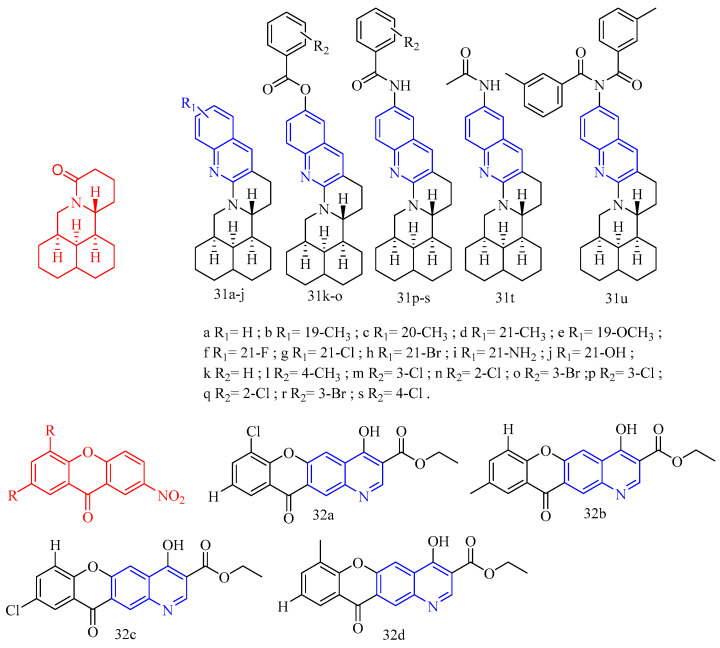
The chemical structures of anti-parasitic compounds **31**–**32**.

Matrine (CF: C_15_H_24_N_2_O, MW: 248.37, (7a*S*,13a*R*,13b*R*,13c*S*)-dodecahydro-1*H*,5*H*,10*H*-dipyrido[2,1-f:3′,2′,1′-ij][1,6]naphthyridin-10-one) is an alkaloid extracted from the dried roots, plants, and fruits of the legume *Sophora flavescens* by ethanol and other organic solvents. Huang and colleagues [32] synthesized quinoline–matrine derivatives **31a**–**u** (Figure 9) (Table 7) and evaluated its acaricidal and insecticidal activities. Interestingly, all quinoline–matrine derivatives (save compound **31c**) outperformed their antecedents in terms of acaricidal activity. In particular, compounds **31g**, **31m**, and **31r** showed the most promising acaricidal action (the MR of compounds **31g**, **31m**, and **31r** at 72 h are 37.1%, 37.1%, and 36.1%, respectively). It is worth noting that the introduction of chlorine atoms at the C-21 position of compound **31a**–**j** is important for the acaricidal activity (the MR of compounds **31d** (including 21-CH_3_), **31f** (including 21-F), **31h** (including 21-Br), **31i** (including 21-NH_2_), and **31j** (including 21-OH) at 72 h are 29.5%, 29.6%, 26.6%, 26.0%, and 26.9%, respectively; the MR of compound **31g** (containing 21-Cl) at 72 h was 37.1%). The insertion of chlorine atoms on the benzoyl oxy group of compound **31k** is required for acaricidal activity in compounds **31k**–**o**. Compound **31r** (having 3-Br on the benzoyl amino group) has a higher acaricidal efficacy than compounds **31p**–**t** (**31p**, **31q**, and **31s** containing chlorine atoms on the benzoyl amino group). The findings suggested that these compounds may have antithrombotic hormone-like properties. All quinoline–matrine derivatives (**31a**–**u**) were more effective against insects than their antecedents. Compounds **31d**–**g**, **31i**, **31p**, and **31s** showed the most effective activity (the FMR of compounds **31d**–**g**, **31i**, **31p**, and **31s** are 65.5%, 62.1%, 65.5%, 69.0%, 62.1%, 65.5%, and 65.5%, respectively). The location of the methyl group is critical for insecticidal efficacy in compounds **31b**–**d**. Compounds **31b** (containing 19-CH_3_) and **31d** (containing 21-CH_3_) have FMRs of 58.6% and 65.5%, respectively; compound **31c** (containing 20-CH_3_) has an FMR of 44.8%. For compounds **31a**–**j**, the quinoline segment of **31a** is the modified position, and the introduction of an appropriate group on the quinoline segment of **31a** can lead to more effective derivatives, such as compounds **31d** (containing 21-CH_3_; FMR: 65.5%), **31e** (containing 19-OCH_3_; FMR: 62.1%), **31f** (containing 21-F; FMR: 65.5%), **31g** (containing 21-Cl; FMR: 69.0%), and **31i** (containing 21-NH_2_; FMR: 62.1%). Although the derivative **31k**–**o** was created by inserting various benzoyl groups into compound 31j, it did not have the same insecticidal action as compound **31a**. When R_2_ is a 3- or 4-chlorine atom, the related compounds **31p** and **31s** have higher insecticidal action than **31a**. Compound **31u** (21-NHCH_3_CO groups; 72 h FMR: 33.2%) showed more activity than compound **31i** (21-NH_2_ groups; 72 h FMR: 26.0%).

Wang and colleagues [33] synthesized 4-hydroxy-11-oxo-11H-chromoene [2,3-*g*] quinoline-3-carboxylic acid ethyl ester **32a**–**d** (Figure 9) (Table 7) and tested their anti-coccidian activity. Compounds **32a**–**d** demonstrated anticoccidial activity against *Eimeria tenella* with ACIs of 147, 123, 133, and 148, respectively.

**Figure 10:**
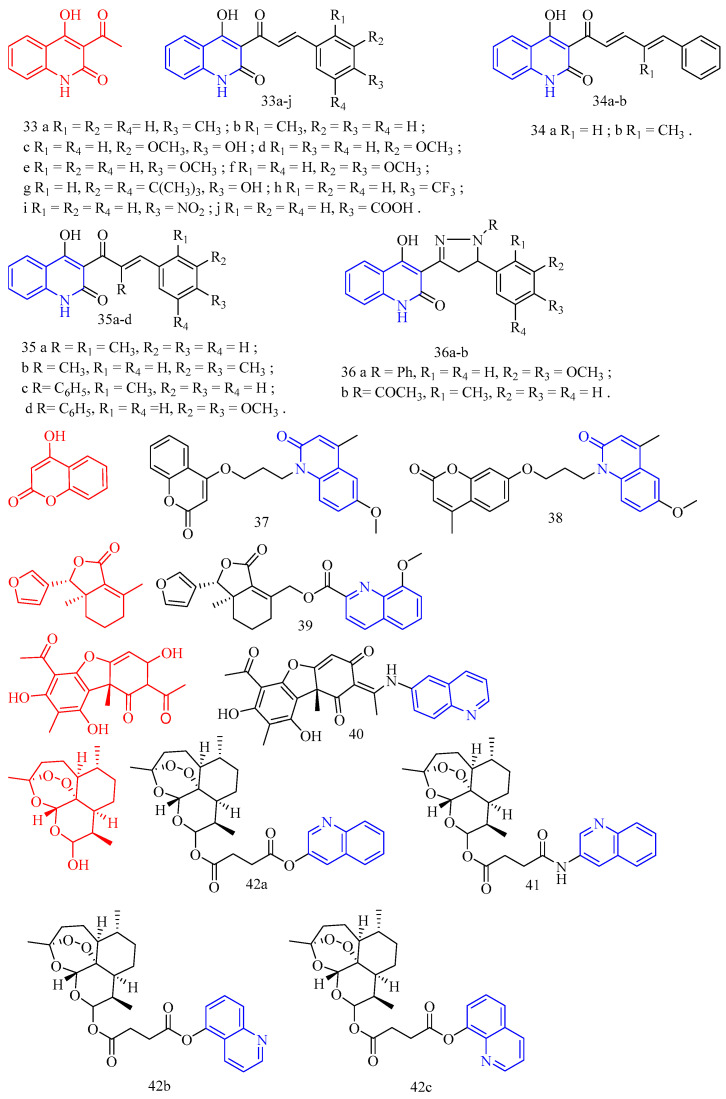
The chemical structures of anti-parasitic compounds **33**–**42**.

Roussaki and colleagues [34] synthesized quinolinone–chalcone derivatives **33**–**36** (Figure 10) (Table 7) and evaluated their biological activities against mammalian T. brucei and Leishmania infantis. Among them, the most effective is compound **33b** (IC_50_ = 2.6 ± 0.1 μM), followed by compounds **33g**, **33c**, **33f**, and **33d** (decreasing potency), all of which contain electron-donating substituents on the B ring of the chalcone group. The data analysis shows that the position and number of these groups contribute to the anti-parasitic properties of the compounds. For the most effective trypanosome agent, compound **33b**, the electron-donating methyl substituent is located at the 2 position of the B ring, and its isomer, compound **33a**, contains a methyl group at the 4 position, which does not affect the growth of trypanosome at a concentration of up to 10 μM. Similarly, compound 33d containing a single methoxy group at the 3 position of the B ring has lethality (IC_50_ = 6.5 ± 0.1 μM). In contrast, the isomer chalcone containing the 4-methoxy group does not affect the growth of the parasite. The presence of two electron-abandoned substituents resulted in a slight increase in trypanosomal activity as follows: Compound **33f** contains two methoxy groups at the 3 and 4 positions of the B ring, with an IC_50_ value of 4.9 ± 0.2 μM, while compound **33c** has an IC_50_ value of 4.9 ± 0.1 μM, and its 3 and 4 positions contain a methoxy group and a hydroxyl group, respectively. The remaining insecticide chalcone, compound **33g**, has a (di-tert-butyl) phenol substitute. Although it is the second most effective anti-trypanosomal structure in the chalcone series (IC_50_ = 3.3 ± 0.1 μM), it shows high cytotoxicity (IC_50_ ≈ 26 μM). For compounds **33b** and **33f**, analogues containing alkyl substituents on the amide nitrogen of the heterocyclic ring of the quinolinone molecule (compounds **35a**–**d**) were synthesized. N-ethyl analogues **35a** and **35b** did not show growth inhibition against Brucella in the blood. Compounds **35c** and **35d**, N-benzyl analogues of chalcones, were less active against Brucella than non-alkyl compounds, although higher than N-ethyl analogues. These results indicate that the hydrogen of heterocyclic amide groups is important in the mechanism of these compounds. Similarly, by adding electron-withdrawing groups (-COOH, -CF_3,_ and -NO_2_) (compounds **33h**, **33i**, and **33j**) to the B ring of chalcone or extending the conjugated system between quinolinone and chalcone patterns (compounds **34a** and **34b**), the quinolinone–chalcone structure was changed in other parts. There was no effect on the growth of trypanosomes at a concentration of 10 μM. The a,b-unsaturated carbonyl system was modified by synthesizing the pyrazoline analogues **36a** and **36b**, significantly increasing anti-parasitic activity against *B. haemolytic.* The IC_50_s of these two compounds were lower than that of the reference drug nifurtimox, so they were the most active against *B. haemolytic* among all the tested compounds in this work. Pyrazoline **36a** (IC_50_ = 1.46 ± 0.1 μM) can be considered a promising anti-parasitic compound because it has no cytotoxicity to THP1 cells. When a series of quinolinone–chalcone hybrids were screened against the astigmatic stage in larval cells, structures (compounds **33**–**34**, **35a**–**b**) were shown to affect the growth of parasites. The most potent compounds were **33g**, **33a**, and **33h**, with IC_50_s of 1.3 ± 0.1, 2.1 ± 0.6, and 3.1 ± 1.0 μM, respectively. Interestingly, the structure–activity relationship against *L. infantum* observed using compound series significantly differs from that against *T. brucei*, and many differences are directly opposite. For example, the shift of the methyl substituent from the 4 position (compound **33a**) to the 2 position (compound **33b**) or the methoxy group from the 4 position (compound **33e**) to the 3 position (compound **33d**) results in a significant decrease in insecticidal activity, which is different from the case of Brucella virus. In addition, the electronic properties of the substituents do not seem to play an important role in anti-Lishmaniasis activity as they do for Brucellosis as follows: Compound **33h** with electron-withdrawing group trifluoromethyl (methyl isomer) at the 4 position of the B ring is the third most effective agent for infantile disease in this series, and derivatives containing-NO_2_ and-COOH (compounds **33i** and **33j**) also affect infantile disease amastigotes. Why these conflicting SAR differences occur is unclear. This may reflect the following different locations of these parasites in mammalian hosts: Brucella is an extracellular pathogen found in the blood of the host, and larvae can invade and grow in the host’s macrophages, residing in an acidic compartment. In addition, this may only be due to the concentration range used in the screening as follows: many leishmaniasis compounds have IC_50_s > 10 μM, which is the highest level used to combat Brucella. The *N*-alkylation of heterocyclic amide groups led to compounds with lower or slightly better anti-Lishmaniasis activity than non-alkylated analogues, indicating that the N-H group may be the key to anti-Lishmaniasis activity, which is also true in anti-filarial activity. Compared with chalcone analogue 9, pyrazoline **36a** has lower activity against infantile lymphoma, while pyrazoline **36b**, an analogue of chalcone 5, is the most active anti-Lishman disease agent among all tested compounds, with an IC_50_ value of 0.71 μM. Notably, pyrazoline **36b** showed the best anti-parasitic activity against both parasites. In order to evaluate their effects on mammalian cells, cytotoxicity tests were performed on differentiated THP-1 macrophages with all compounds. Among the most effective agents (IC_50_ < 10 μM), compounds **33a**, **33b**, **33d**, **33f**, **33h**, and **36a** had no growth inhibitory effect on THP-1 cells at concentrations of 30 μM (**33b**, **33d**, and **33f**) or 50 μM (**33a**, **33h**, and **36a**), while the IC_50_s of compounds **33c** and **33g** were about 20 μM. Due to problems related to the solubility of compounds in DMSO and other common solvents, the exact IC_50_ cytotoxicity data cannot be determined. A comparison of efficacy against parasites and mammalian strains showed that pyrazoline **36a** and chalcone 5 were the most effective trypanosome preparations, while chalcone analogue **36a** was an interesting guide structure for *L. infantum*. In summary, compounds **36a** and **36b** have obvious anti-parasitic activity against *T. brucei* blood in vitro. Although pyrazoline **36b** showed the best activity against both parasites and was the most effective compound among all the parasites tested in this work, its high cytotoxicity to mammalian cells prohibits further consideration as a lead compound. In contrast, pyrazoline **36a** should be considered a drug to induce trypanosomiasis because of its high anti-parasitic activity and no cytotoxicity.

Pan and colleagues [35] synthesized coumarin derivatives **37**–**38** (Figure 10) (Table 7) and tested the nematicidal activity of these derivatives. Compounds **37** and **38** showed significant broad spectrum nematicidal activity. (The LC_50_s for *M. incognita*, *Ditylenchus destructor*, *Bursaphelenchus mucronatus*, *B. xylophilus,* and *Aphelenchoides besseyi* of compound **37** were are 64.0, 52.9, 97.9, 103.2, and 95.2 μmol/L, respectively. The LC_50_s for *M. incognita*, *Ditylenchus destructor*, *Bursaphelenchus mucronatus*, *B. xylophilus,* and *Aphelenchoides besseyi* of compound **38** were 42.4, 68.0, 77.8, 145.5, and 120.7 μmol/L, respectively).

Fraxinellone (CF: C_14_H_16_O_3_, MW: 232.275, 7-dimethyl-3a,4,5,6-tetrahydro-2-benzofuran-1(3H)-one) is isolated from the root bark of the Brassica plant *Dictamnus dasycarpus*. Guo and colleagues [36] synthesized fraxinellone–quinoline derivative **39** (Figure 10) (Table 7) and tested the in vivo insecticidal activity of isolated Mycobacterium pre-3 instar larvae. Compound **39** showed more promising insecticidal activity than positive contrast (the *M. separata* on leaves mortality rate for 10 days, 25 days, and 35 days are 20.7%, 48.1%, and 63.0%, respectively).

Guo and colleagues [37] synthesized usnea acid quinoline derivative **40** (Figure 10) (Table 7) and tested its anti-*T. gondii* activity. Compound **40** had lower cytotoxicity and a higher selectivity index, indicating that its activity was superior to that of the lead drug and positive control drugs. The results of tachyzoite content assessment in mice abdomen showed that the compound **40** inhibition rate of abdominal tachyzoites in mice reached 55.2% (*p* < 0.01), 58.3% (*p* < 0.01), and 64.6% (*p* < 0.001), respectively, the numbers of tachyzoites were significantly reduced, respectively. At the same concentration, (+)-usnic acid and **40** inhibited tachyzoites more effectively than the positive control; moreover, compound **40** has better anti-T. The activity of Toxoplasma gondii is higher than the natural product (+)-usnic acid. The toxicity of compound **40** was further investigated by measuring the ALT and AST levels in the serum of KM mice. Compared with the normal group, the serum ALT level of the Toxoplasma-infected mice was significantly increased (*p* < 0.05). The serum ALT levels were significantly lower (*p* < 0.01) in the compound **40**-treated group than in the (+)-usnic acid-treated group.

Dihydroartemisinin (CF: C_15_H_24_O_5_, MW: 284.35, (4*S*,5*R*,8*S*,9*R*,10*S*,12*R*,13*R*)-1,5,9-trimethyl-11,14,15,16-tetraoxatetracyclo hexadecan-10-ol) is an artemisinin derivative that has a powerful and rapid killing impact on the red internal stage of Plasmodium and can reduce clinical attacks and symptoms swiftly. Deng and colleagues [38] synthesized dihydroartemisinin quinoline derivatives **41**–**42** (Figure 10) (Table 7) and evaluated their anti-Toxoplasma activity. Compounds **41** and **42a**–**c** were more effective against Toxoplasma than dihydroartemisinin (The selectivity index of compounds **41a** and **42a**–**c** was 0.84, 1.02, 0.43, and 1.02).

**Table 7:** Quinoline derivatives with anti-parasitic activity.

Compd.	Activity	Target	Origin	Ref
**27a**	anti-*T. brucei* IC_50_ = 0.37 μg/mL anti-*L. Mexicana* IC_50_ = 3.86 μg/mL	-	synthetic	[29]
**27b**	anti-*T. brucei* IC_50_ = 0.39 μg/mL anti-*L. Mexicana* IC_50_ = 3.39 μg/mL	-	synthetic	[29]
**27c**	anti-*T. brucei* IC_50_ = 0.40 μg/mL anti-*L. Mexicana* IC_50_ = 3.45 μg/mL	-	synthetic	[29]
**28a**	anti-trichomonas IC_50_ = 22.2 μM	-	synthetic	[30]
**28b**	-	-	synthetic	[30]
**28c**	anti-trichomonas IC_50_ = 11.3 μM	-	synthetic	[30]
**29a**	Leishmanicidal EC_50_ = 11.79 μg/mL Trypanocidal EC_50_ = 35.08 μg/mL	-	synthetic	[30]
**29b**	Leishmanicidal EC_50_ = 6.24 μg/mL Trypanocidal EC_50_ = 17.62 μg/mL	-	synthetic	[31]
**29c**	Leishmanicidal EC_50_ = 12.37 μg/mL Trypanocidal EC_50_ = 15.79 μg/mL	-	synthetic	[31]
**29d**	Leishmanicidal EC_50_ = 8.53 μg/mL Trypanocidal EC_50_ = 37.61 μg/mL	-	synthetic	[31]
**29e**	Leishmanicidal EC_50_ = 16.41 μg/mL Trypanocidal EC_50_ = 15.12 μg/mL	-	synthetic	[31]
**29f**	Leishmanicidal EC_50_ = 22.0 μg/mL Trypanocidal EC_50_ = 54.95 μg/mL	-	synthetic	[31]
**30a**	Leishmanicidal EC_50_ = 6.11 μg/mL Trypanocidal EC_50_ = 4.09 μg/mL	-	synthetic	[31]
**30b**	Leishmanicidal EC_50_ = 16.18 μg/mL Trypanocidal EC_50_ > 20 μg/mL	-	synthetic	[31]
**30c**	Leishmanicidal EC_50_ = 2.36 μg/mL Trypanocidal EC_50_ > 2 μg/mL	-	synthetic	[31]
**31a**	Corrected mortality rate 48 h FMR 8.7% 72 h FMR 32.7%	-	synthetic	[32]
**31b**	Corrected mortality rate 48 h FMR 7.8% 72 h FMR 28.0%	-	synthetic	[32]
**31c**	Corrected mortality rate 48 h FMR 2.8% 72 h FMR 19.2%	-	synthetic	[32]
**31d**	Corrected mortality rate 48 h FMR 7.5% 72 h FMR 29.5%	-	synthetic	[32]
**31e**	Corrected mortality rate 48 h FMR 6.9% 72 h FMR 24.4%	-	synthetic	[32]
**31f**	Corrected mortality rate 48 h FMR 6.9% 72 h FMR 29.6%	-	synthetic	[32]
**31g**	Corrected mortality rate 48 h FMR 7.7% 72 h FMR 37.1%	-	synthetic	[32]
**31h**	Corrected mortality rate 48 h FMR 6.3% 72 h FMR 26.6%	-	synthetic	[32]
**31i**	Corrected mortality rate 48 h FMR 5.0% 72 h FMR 26.0%	-	synthetic	[32]
**31j**	Corrected mortality rate 48 h FMR 6.5% 72 h FMR 26.9%	-	synthetic	[32]
**31k**	Corrected mortality rate 48 h FMR 3.8% 72 h FMR 26.5%	-	synthetic	[32]
**31l**	Corrected mortality rate 48 h FMR 2.9% 72 h FMR 20.6%	-	synthetic	[32]
**31m**	Corrected mortality rate 48 h FMR 15.5% 72 h FMR 37.1%	-	synthetic	[32]
**31n**	Corrected mortality rate 48 h FMR 7.2% 72 h FMR 30.7%	-	synthetic	[32]
**31o**	Corrected mortality rate 48 h FMR 5.9% 72 h FMR 28.7%	-	synthetic	[32]
**31p**	Corrected mortality rate 48 h FMR 9.6% 72 h FMR 30.9%	-	synthetic	[32]
**31q**	Corrected mortality rate 48 h FMR 3.2% 72 h FMR 22.5%	-	synthetic	[32]
**31r**	Corrected mortality rate 48 h FMR 9.5% 72 h FMR 36.1%	-	synthetic	[32]
**31s**	Corrected mortality rate 48 h FMR 6.4% 72 h FMR 26.7%	-	synthetic	[32]
**31t**	Corrected mortality rate 48 h FMR 4.8% 72 h FMR 26.6%	-	synthetic	[32]
**31u**	Corrected mortality rate 48 h FMR 8.6% 72 h FMR 33.2%	-	synthetic	[32]
**32a**	ACI = 147	-	synthetic	[33]
**32b**	ACI = 123	-	synthetic	[33]
**32c**	ACI = 133	-	synthetic	[33]
**32d**	ACI = 148	-	synthetic	[33]
**33a**	*T. brucei* IC_50_ > 10 μM *L. infantum* IC_50_ = 2.1 μM	FRD	synthetic	[34]
**33b**	*T. brucei* IC_50_ = 2.6 μM *L. infantum* IC_50_ > 50 μM	FRD	synthetic	[34]
**33c**	*T. brucei* IC_50_ = 4.9 μM *L. infantum* IC_50_ = 11.5 μM	FRD	synthetic	[34]
**33d**	*T. brucei* IC_50_ = 6.5 μM *L. infantum* IC_50_ = 28.4 μM	FRD	synthetic	[34]
**33e**	*T. brucei* IC_50_ > 10 μM *L. infantum* IC_50_ = 12.7 μM	FRD	synthetic	[34]
**33f**	*T. brucei* IC_50_ = 4.9 μM *L. infantum* IC_50_ = 7.5 μM	FRD	synthetic	[34]
**33g**	*T. brucei* IC_50_ = 3.3 μM *L. infantum* IC_50_ = 1.3 μM	FRD	synthetic	[34]
**33h**	*T. brucei* IC_50_ > 10 μM *L. infantum* IC_50_ = 3.1 μM	FRD	synthetic	[34]
**33i**	*T. brucei* IC_50_ > 10 μM *L. infantum* IC_50_ = 26.9 μM	FRD	synthetic	[34]
**33j**	*T. brucei* IC_50_ > 10 μM *L. infantum* IC_50_ = 18.4 μM	FRD	synthetic	[34]
**34a**	*T. brucei* IC_50_ > 10 μM *L. infantum* IC_50_ > 50 μM	FRD	synthetic	[34]
**34b**	*T. brucei* IC_50_ > 10 μM *L. infantum* IC_50_ = 20.0 μM	FRD	synthetic	[34]
**35a**	*T. brucei* IC_50_ > 10 μM *L. infantum* IC_50_ = 24.8 μM	FRD	synthetic	[34]
**35b**	*T. brucei* IC_50_ > 10 μM *L. infantum* IC_50_ > 50 μM	FRD	synthetic	[34]
**35c**	*T. brucei* IC_50_ = 6.17 μM *L. infantum* IC_50_ > 25 μM	FRD	synthetic	[34]
**35d**	*T. brucei* IC_50_ = 5.68 μM *L. infantum* IC_50_ > 25 μM	FRD	synthetic	[34]
**36a**	*T. brucei* IC_50_ = 1.46 μM *L. infantum* IC_50_ = 13.46 μM	FRD	synthetic	[34]
**36b**	*T. brucei* IC_50_ = 1.43 μM *L. infantum* IC_50_ = 0.71 μM	FRD	synthetic	[34]
**37**	*M. incognita* LC_50_ = 64.0 μmol/L *Ditylenchus destructor* LC_50_ = 52.9 μmol/L *Bursaphelenchus mucronatus* LC_50_ = 97.9 μmol/L *B. xylophilus* LC_50_ = 103.2 μmol/L *Aphelenchoides besseyi* LC_50_ = 95.2 μmol/L	-	synthetic	[35]
**38**	*M. incognita* LC_50_ = 42.4 μmol/L *Ditylenchus destructor* LC_50_ = 68.0 μmol/L *Bursaphelenchus mucronatus* LC_50_ = 77.8 μmol/L *B. xylophilus* LC_50_ = 145.5 μmol/L *Aphelenchoides besseyi* LC_50_ = 120.7 μmol/L	-	synthetic	[35]
**39**	*M. separata* on leaves mortality rate 10 days 20.7% 25 days 48.1% 35 days 63.0%	-	synthetic	[36]
**40**	inhibition rate of abdominal tachyzoites in mice 55.2% (*p* < 0.1), 58.3% (*p* < 0.01) 64.6% (*p* < 0.001)	-	synthetic	[37]
**41**	selectivity index 0.84	T*g*CDPK1	synthetic	[38]
**42a**	selectivity index 1.02	T*g*CDPK1	synthetic	[38]
**42b**	selectivity index 0.43	T*g*CDPK1	synthetic	[38]
**42c**	selectivity index 1.02	T*g*CDPK1	synthetic	[38]

Summary: The anti-*T.brucei* activity of Cinchonasine derivative **27a** was the strongest, and the IC_50_ value was 0.37 μg/mL. The anti-*L.mexicana* activity of derivative **27b** was the strongest, and the IC_50_ value was 3.39 μg/mL.

The EC_50_ values of compound **29f** against Leishmania (V) panamensis and Trypanosoma cruzi were 6.11 and 4.09 μg/mL, respectively.

The quinolinone–chalcone derivatives **36a** and **36b** showed the strongest inhibitory effect on *T.brucei*, with IC_50_ values of 1.46 and 1.43 μM. **36a** has no obvious cytotoxicity to THP1 cells and is an anti-parasitic compound with development value. Compounds **42a** and **42c** were more effective against Toxoplasma than dihydroartemisinin (The selectivity index of compounds **42a** was 1.02). The anti-parasitic activity of natural products was enhanced by adding quinoline to natural products; however, the mechanism of action of these compounds has not been studied, so these compounds need further study.

### 3.8. Antimalarial Activity

**Figure 11:**
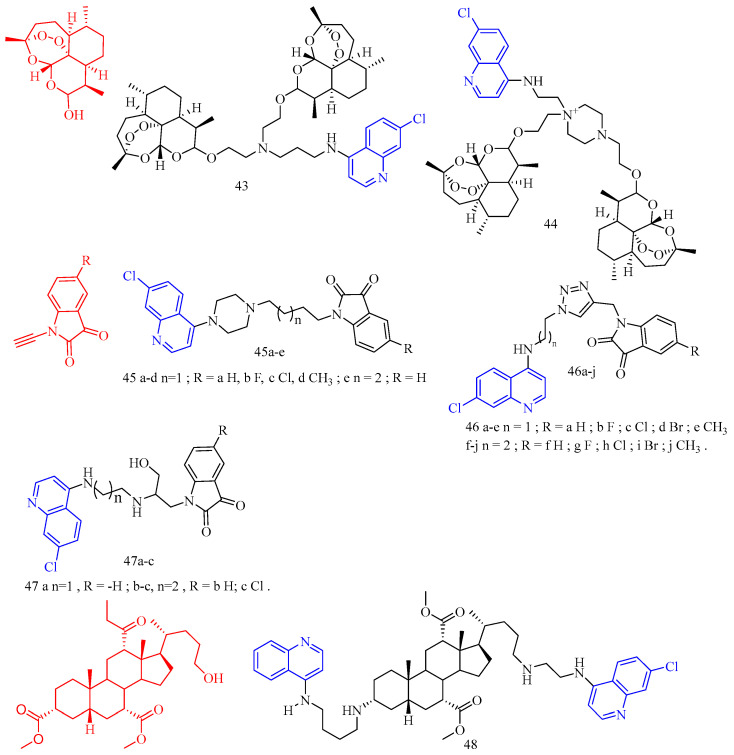
The chemical structures of antimalarial and anticancer compounds **43**–**48**.

Artemisinin (CF: C_15_H_22_O_5_, MW: 282.34, (3*R*,5a*S*,6*R*,8a*S*,9*R*,10*S*,12*R*,12a*R*)-Decahydro-3,6,9-trimethyl-3,12-oxo-12H-pyrano[4,3-j]-1,2-Benzodixepin-10-one) is mostly derived by direct extraction from Artemisia annua or by extracting artemisinic acid from Artemisia annua and then semi-synthetically obtaining it. Lombard and colleagues [39] synthesized two quinoline–artemisinin derivatives **43** and **44** (Figure 11) (Table 8) and evaluated their antimalarial activity. The in vivo anti-plasma parasite activity of hybrid dimers **43** and **44** was stronger than the positive control, with in vitro IC_50_s of 8.7 and 29.5 μM against the 3D7 strain, respectively.

Raj and colleagues [40] synthesized a series of piperazine-coupled 7-chloroquinoline–isatin derivatives **45a**–**e** (Figure 11) (Table 8), and their antimalarial and anti-chloroquine activities against Plasmodium falciparum were evaluated. However, compounds **45a**–**e** have shown strong antimalarial activity with IC_50_s of 1.12, 1.17, 0.27, 0.81, and 0.36 μM, respectively. Unfortunately, all of the compounds had lower activities than the positive control.

Raj and colleagues [41] performed the synthesis and antimalarial activity of a 1H-1,2,3-triazole-tethered 7-chloroquinoline–isatin derivatives **46a**–**j** (Figure 11) (Table 8). Compounds **46a**–**j** have longer alkyl chains between the 7-chloroquinoline and isatin groups and have better anti-plasma action. Unfortunately, the IC_50_s of the tested compounds **46a**–**j** were all greater than 1 μM, which was less effective than the positive control or artemisinin.

Nisha and colleagues [42] synthesized a series of β-amino alcohol-linked 4-aminoquinoline–indigo derivatives **47a**–**c** (Figure 11) (Table 8) and evaluated their activity against CQ-resistant *P. falciparum* W2. The most active compounds were **47b**–**c**, with IC_50_s of 11.8 and 13.5 μM, respectively.

Videnović and colleagues [43] synthesized 4-amino-7-chloroquinoline (4,7-ACQ) bile salt derivative **48** (Figure 11) (Table 8) and determined its antimalarial activity. Compound **48** demonstrated the highest antimalarial activity, with a 95% inhibition rate and a Ki of 0.103 μM.

**Figure 12:**
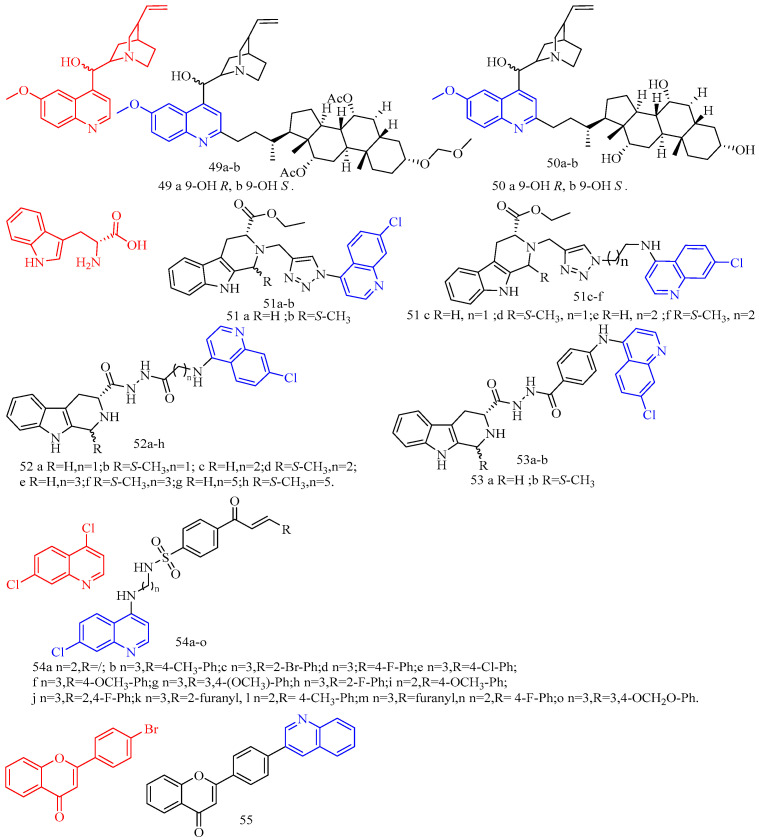
The chemical structures of antimalarial compounds **49**–**55**.

Leverrier and colleagues [44] synthesized cinchona–alkaloid derivatives **49**–**50** (Figure 12) (Table 8) and determined their in vitro activity against *Trypanosoma brucei*. Compounds **49a**–**b** and **50a**–**b** emerged as the most promising anti-*Trypanosoma brucei* due to their low cytotoxicity and IC_50_s of 1.47, 0.64, 0.69, and 0.61 μM, respectively.

1,2,3,4-Tetrahydro-β-carbolines (THβCs) (CF: C_11_H_12_N_2_, MW: 172.226, 1,2,3,4-tetrahydro-9h-pyrido[3,4-b]indole) represent a class of privileged structural motifs found in many pharmacologically active natural compounds possessing potential anticancer and antimalarial activities. Sharma and colleagues [45] synthesized 1*H*-1,2,3-triazole/hydrazole-integrated tetrahydro-β-carboline-4-aminoquinoline compounds **51a**–**f**, **52a**–**h**, and **53a**–**b** (Figure 12) (Table 8) and evaluated their activity against the chloroquine-resistant (CQ) strain of Plasmodium falciparum W2. Although all compounds were not as active as the positive control medication, including the quinoline nucleus considerably increased the antimalarial efficacy of the THC nucleus. The activity of the synthesized conjugates depends on the nature of the substitution at the C-1 position of the THC nucleus, the type of function introduced as a linker, and the length of the alkyl chain, according to the structure–activity relationship (SAR). An analysis of the SAR of 1*H*-1,2,3-triazole tethered THC-4-aminoquinoline conjugates revealed an increase in the antimalarial activity with the introduction of flexible alkyl chains on the aminoquinoline core, as evidenced by the scaffolds **51c**–**f** being more active than compounds **51a**–**b** (The IC_50_s of compounds **51a**–**b** are 4.02 μM, 9.28 μM). The antimalarial activity of the aliphatic hydrazide-linked conjugates **52a**–**h** increased with increasing alkyl chain length, although the type of the substituent at the C-1 position of THC did not appear to alter the activity profile. Interestingly, replacing the alkyl chain with an aryl core enhanced antimalarial activity, as seen by compound **53a**, which has an IC_50_ of 0.61 μM. The antimalarial activity of the -carboline and quinolinyl precursors used in this synthesis was also investigated. Compounds **53a**–**b** (acyl hydrazide precursors) and **51c**, **51e** (1*H*-12,3-triazole-like precursors) were tested on mammalian Vero cells to determine if the observed activity was due to inherent antimalarial activity or cytotoxicity. The 1*H*-1,2,3-triazole-linked conjugates **51c** and **51e** were clearly non-cytotoxic, with SI > 300, whereas the acyl hydrazide-linked compounds **53a**–**b** were somewhat cytotoxic to mammalian Vero cells. The most potent and non-cytotoxic compound **51e** exhibited the best properties, including a C-1 unsubstituted THC core, a 1H-1,2,3-triazole core, and propyl as a flexible linker, with an IC_50_ of 0.50 μM and a selectivity index of 495.76.

Vinindwa and colleagues [46] synthesized chalcone–quinoline derivatives **54a**–**o** (Figure 12) (Table 8) and evaluated their antimalarial activity. With IC_50_s ranging from 0.10 to 4.45 μM, all compounds showed potent activity against the *Plasmodium falciparum* NF54 sensitive strain. The fluorine-substituted molecular derivatives **54d**, **54h**, and **54n** displayed higher activity than the unsubstituted molecular derivative **54a** (IC_50_ = 1.67 μM), suggesting the relevance of the electronic effect imparted by the more electronegative fluorine atoms. Other halogens with similar trends included bromine **54c** (IC_50_ = 0.10 μM), chlorine **54e** (IC_50_ = 0.10 μM), and methoxy **54f** (IC_50_ = 0.11 μM). The most active compounds were **54c**, **54e**, and **54f**, with IC_50_s of 0.10, 0.10, and 0.11 μM, respectively. The inhibitory properties of the compounds against the multidrug-resistant K1 strain of *Plasmodium falciparum* were investigated further. Compound **54f**’s resistivity index RI = 5.36 (IC_50_ = 0.59 μM) was roughly double that of the positive control, which was the leading compound in this study. Compounds **54c** and **54e**, on the other hand, displayed poor activity against the K1 strain, with IC_50_s of 2.97 and 6 μM, respectively. Compounds with three methylene (n = 3) as linkers had higher efficacy against *Plasmodium falciparum* than compounds with two methylene (n = 2). Compared to compound **54i** (n = 2), the addition of an extra CH_2_ group (n = 3) in compound 54f enhanced its activity by nearly five times, while compound **54d** (n = 2 IC_50_ = 0.28 μM) increased by two times. Furthermore, practically all molecular derivatives **54b**–**f** and **54g**–**j** with three methylene linkers are the most active in the series, with IC_50_s ranging from 0.10 to 0.86 μM. Compounds **54b** and **54l**, which have methyl groups at the para position and have the same activity (The IC_50_s were 0.37 and 0.39 μM, respectively), show no effect on activity with smaller polarity. Compounds **54k** and **54m** have poor activity and contain 2-furanyl and ferrocenyl aromatic rings, demonstrating the relevance of chalcone units in the overall activity of the molecule. Most compounds were insoluble except for compounds **54b**, **54i**, **54j**, **54l**, and **54o**, with IC_50_ values less than 5 μM.

Flavonoids are secondary plant metabolites that are frequently found in nature. Flavonoids are a yellow pigment formed from a core of flavonoids (2-phenylchromones), which includes flavonoid isomers and their hydrogenation and reduction products, such as C6-C3-C6. Rodrigues and colleagues [47] synthesized the flavonoid quinoline derivative **55** (Figure 12) (Table 8) and determined its antimalarial activity. Compound **55** was tested in vitro for antimalarial activity against the chloroquine-resistant *Plasmodium falciparum* W2 strain. Compound **55** had moderate antimalarial activity, with an IC_50_ of 5.17 μM.

**Table 8:** Quinoline derivatives with antimalarial activity.

Compd.	Activity	Origin	Ref
**43**	Anti-plasma parasite IC_50_ = 8.7 μM	synthetic	[39]
**44**	Anti-plasma parasite IC_50_ = 29.5 μM	synthetic	[39]
**45a**	Antimalarial activity IC_50_ = 1.12 μM	synthetic	[40]
**45b**	Antimalarial activity IC_50_ = 1.17 μM	synthetic	[40]
**45c**	Antimalarial activity IC_50_ = 0.27 μM	synthetic	[40]
**45d**	Antimalarial activity IC_50_ = 0.81 μM	synthetic	[40]
**45e**	Antimalarial activity IC_50_ = 0.36 μM	synthetic	[40]
**46a**	Antimalarial activity IC_50_ > 5 μM	synthetic	[41]
**46b**	Antimalarial activity IC_50_ > 5 μM	synthetic	[41]
**46c**	Antimalarial activity IC_50_ > 5 μM	synthetic	[41]
**46d**	Antimalarial activity IC_50_ > 5 μM	synthetic	[41]
**46e**	Antimalarial activity IC_50_ > 5 μM	synthetic	[41]
**46f**	Antimalarial activity IC_50_ = 3.07 μM	synthetic	[41]
**46g**	Antimalarial activity IC_50_ = 2.30 μM	synthetic	[41]
**46h**	Antimalarial activity IC_50_ = 1.37 μM	synthetic	[41]
**46i**	Antimalarial activity IC_50_ = 1.73 μM	synthetic	[41]
**46j**	Antimalarial activity IC_50_ = 1.63 μM	synthetic	[41]
**47a**	Antimalarial activity IC_50_ = 24.9 nM	synthetic	[42]
**47b**	Antimalarial activity IC_50_ = 11.8 nM	synthetic	[42]
**47c**	Antimalarial activity IC_50_ = 13.5 nM	synthetic	[42]
**48**	Antimalarial activity Ki = 0.103 μM	synthetic	[43]
**49a**	Anti-*Trypanosoma brucei* IC_50_ = 1.47 μM	synthetic	[44]
**49b**	Anti-*Trypanosoma brucei* IC_50_ = 0.64 μM	synthetic	[44]
**50a**	Anti-*Trypanosoma brucei* IC_50_ = 0.69 μM	synthetic	[44]
**50b**	Anti-*Trypanosoma brucei* IC_50_ = 0.61 μM	synthetic	[44]
**51a**	Antimalarial activity IC_50_ = 9.28 μM	synthetic	[45]
**51b**	Antimalarial activity IC_50_ = 4.02 μM	synthetic	[45]
**51c**	Antimalarial activity IC_50_ = 0.86 μM	synthetic	[45]
**51d**	Antimalarial activity IC_50_ = 0.93 μM	synthetic	[45]
**51e**	Antimalarial activity IC_50_ = 0.49 μM	synthetic	[45]
**51f**	Antimalarial activity IC_50_ = 1.37 μM	synthetic	[45]
**52a**	Antimalarial activity IC_50_ = 4.4 μM	synthetic	[45]
**52b**	Antimalarial activity IC_50_ = 1.73 μM	synthetic	[45]
**52c**	Antimalarial activity IC_50_ = 5.0 μM	synthetic	[45]
**52d**	Antimalarial activity IC_50_ = 3.1 μM	synthetic	[45]
**52e**	Antimalarial activity IC_50_ = 2.0 μM	synthetic	[45]
**52f**	Antimalarial activity IC_50_ = 3.1 μM	synthetic	[45]
**52g**	Antimalarial activity IC_50_ = 3.4 μM	synthetic	[45]
**52h**	Antimalarial activity IC_50_ = 2.2 μM	synthetic	[45]
**53a**	Antimalarial activity IC_50_ = 1.8 μM	synthetic	[45]
**53b**	Antimalarial activity IC_50_ = 0.61 μM	synthetic	[45]
**54a**	Antimalarial activity IC_50_ = 0.45 μM	synthetic	[46]
**54b**	Antimalarial activity IC_50_ = 0.37 μM	synthetic	[46]
**54c**	Antimalarial activity IC_50_ = 0.10 μM	synthetic	[46]
**54d**	Antimalarial activity IC_50_ = 0.28 μM	synthetic	[46]
**54e**	Antimalarial activity IC_50_ = 0.10 μM	synthetic	[46]
**54f**	Antimalarial activity IC_50_ = 0.11 μM	synthetic	[46]
**54g**	Antimalarial activity IC_50_ = 0.32 μM	synthetic	[46]
**54h**	Antimalarial activity IC_50_ = 0.86 μM	synthetic	[46]
**54i**	Antimalarial activity IC_50_ = 0.49 μM	synthetic	[46]
**54j**	Antimalarial activity IC_50_ = 0.50 μM	synthetic	[46]
**54k**	Antimalarial activity IC_50_ = 4.45 μM	synthetic	[46]
**54l**	Antimalarial activity IC_50_ = 0.39 μM	synthetic	[46]
**54m**	Antimalarial activity IC_50_ = 1.53 μM	synthetic	[46]
**54n**	Antimalarial activity IC_50_ = 0.57 μM	synthetic	[46]
**54o**	Antimalarial activity IC_50_ = 0.69 μM	synthetic	[46]
**55**	Antimalarial activity IC_50_ = 5.17 μM	synthetic	[47]

Summary: Quinoline has always been a key scaffold for antimalarial research. Many antimalarial drugs, such as chloroquine and primaquine, contain a quinoline structure. With the increasing resistance of Plasmodium falciparum, there is an urgent need to develop new strategies and new antimalarial compounds to overcome the growing resistance. These include drug combination, drug reuse, and the use of chemical sensitizers (resistance reversal agents) and the development of new analogues, both of which involve the synthesis of new quinoline analogues.

7-chloroquinoline–isatin derivatives **45c** showed strong antimalarial activity with IC_50_ of 0.27 μM. Compounds **54c** and **54e** also showed significant antimalarial activity with IC_50_ of 0.10 μM.

The introduction of quinoline by isatin and chalcone can significantly increase antimalarial activity. Unfortunately, the antimalarial mechanism of these compounds has not been studied.

### 3.9. Antibacterial Activity

**Figure 13:**
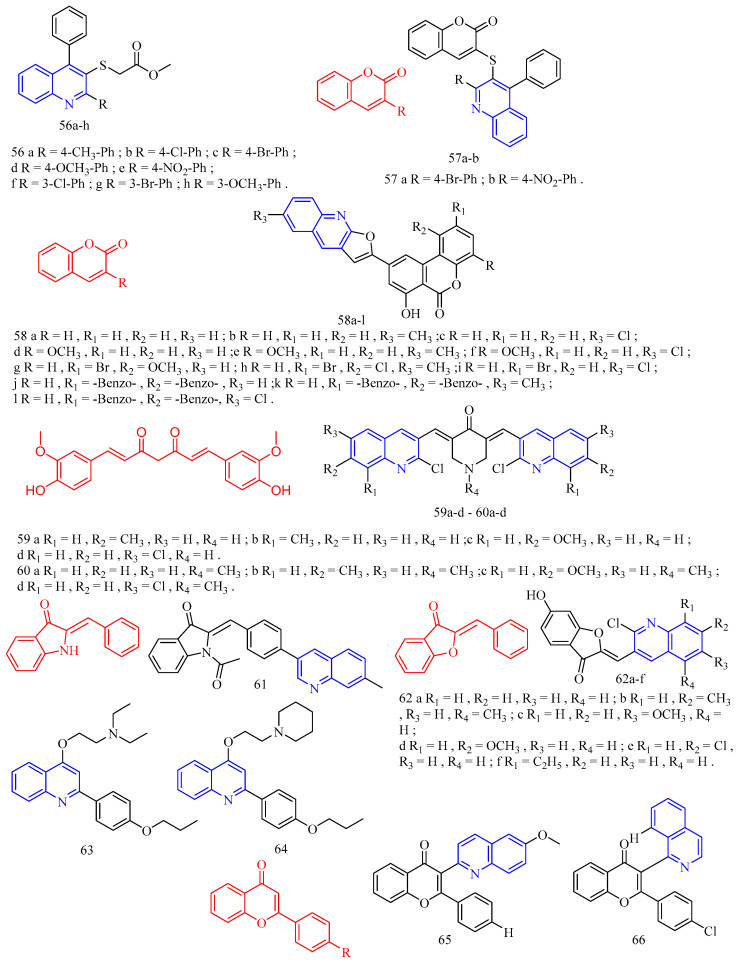
The chemical structures of antibacterial compounds **56**–**66**.

Paul and colleagues [48] synthesized a sulfur-linked quinoline derivative **56**–**57** (Figure 13) (Table 9) and preliminarily evaluated its in vitro antibacterial activity against Gram-positive Staphylococcus aureus (ATCC 11632) and Gram-negative *Escherichia coli* (ATCC 25922), and antifungal activity against *Candida albicans* (ATCC 90028). The results showed that most compounds had moderate to good antibacterial and antifungal activities (6.25–50.0 μg/mL). Compounds **56b**–**h** and **57a**–**b** have moderate to good activity, and the inhibition zone is equivalent to that of the positive control against *Escherichia coli*. Compared with the positive control, compounds **56a**, **56c**, **56d**, **56e**, **56f**, **56g**, **56h**, and **57a** showed moderate activity against S.aureus. In the antifungal activity evaluation, compared with the positive control, compounds **56a**, **56c**, **56e**, **56f**, **56g**, **56h**, **57a**, and **57b** showed moderate activity against *Candida albicans*. Nitro, brominated, or chlorinated compounds have comparable activity to standard drugs.

Patel and colleagues [49] synthesized a series of novel 7-hydroxy-9- (furo [2,3-b] quinoline-2-yl) 6H-benzo [*c*] coumarin derivatives **58a**–**l** (Figure 13) (Table 9) against two Gram-positive bacteria *Staphylococcus aureus* (MTCC 96) and *Bacillus subtilis* (MTCC 441) and two Gram-negative bacteria *Escherichia coli* (MTCC 443) and *Salmonella* (MTCC 98) in vitro antibacterial activity. The antifungal activities of *Candida albicans* (MTCC 227) and *Aspergillus niger* (MTCC 282) were also evaluated in vitro. Ampicillin, chloramphenicol, and norfloxacin were used as standard antimicrobial agents. Glibenclamide and nystatin were used as standard antifungal agents. Compared with standard antimicrobial agents, all compounds are active against Gram-negative bacteria and fungi. Upon evaluating the antimicrobial activity data, it was observed that compounds **58c**, **58h**, and **58k** (MIC = 200 μg/mL) showed good activity compared to ampicillin (MIC = 250 μg/mL) against Gram-positive bacteria *B. subtilis*. The compounds **58b**, **58d**, **58e**, **58f**, **58g**, and **58l** (MIC = 250 μg/mL) exerted equipotent activity against Gram-positive bacteria *B. subtilis*. against *S. aureus*, Compounds **58j** and **58l** (MIC = 100 μg/mL) and **58d**, **58e**, and **58k** (MIC = 125 μg/mL) exhibited moderate activity compared to ampicillin (MIC = 250 μg/mL) against Gram-positive bacteria *S. aureus*. Compounds **58b** and **58g** (MIC = 200 μg/mL) showed better activity compared to ampicillin (MIC = 250 μg/mL) against Gram-positive bacteria *S. aureus.* Compounds **58a**, **58c**, **58h**, and **58i** (MIC = 250 μg/mL) were found equipotent to ampicillin (MIC = 250 μg/mL) against Gram-positive bacteria *S. aureus.* Compounds **58c**, **58d**, **58f**, and **58j** (MIC = 62.5 μg/mL) exhibited outstanding activity compared to ampicillin (MIC = 100 μg/mL) against Gram-negative bacteria *E. coli* and *S. typhi*, respectively. The compounds **58a**, **58f**, **58g**, **58h**, and **58k** (MIC = 100 μg/mL) and compounds **58a**, **58c**, **58d**, and **58j** (MIC = 100 μg/mL) were found equipotent compared to ampicillin (MIC = 100 μg/mL) against *E. coli* and *S. typhi,* respectively. Compound **58i** and **58g** (MIC = 200 μg/mL) and compounds **58c**, **58f**, and **58h** (MIC = 250 μg/mL) were found to be more active against *C. albicans* compared to griseofulvin (MIC = 500 μg/mL). Compounds **58a** and **58b** (MIC = 500 μg/mL) were found equipotent to griseofulvin (MIC = 500 μg/mL) against *C. albicans*. It is perceived from the antimicrobial data that almost all the tested derivatives **58a**–**l** was found to be potent against the Gram-positive bacterial strains. Among all the tested compounds, the compounds **58c**, **58d**, **58f**, and **58j** were found to be more efficient members of the series. Most synthesized compounds were active against Gram-positive bacteria viz. *Bacillus subtilis* (MTCC 441) and *Staphylococcus aureus* (MTCC 96), Gram-negative bacteria viz. *Escherichia coli* (MTCC 443) and *Salmonella typhi* (MTCC 98). Some of the synthesized compounds were found sufficiently potent to inhibit fungal pathogen viz. *Candida albicans* (MTCC 227).

Curcumin (CF: C_21_H_20_O_6_, MW: 368.39, (1*E*,6*E*)-1,7-bis(4-hydroxy-3-methoxyphenyl)hepta-1,6-diene-3,5-dione) is a diketone compound extracted from the rhizomes of some plants in the *Zingiberaceae* and *Araceae*, and is a very rare pigment with diketone structure in the plant kingdom. Subhedar and colleagues [50] synthesized quinoline–curcumin derivatives **59**–**60** (Figure 13) (Table 9), and evaluated their anti-tuberculosis activity against MTB and *M. bovis* BCG in vitro. Rifampicin as a positive control. Overall, the synthesized compounds showed excellent selectivity against *M. bovis* BCG compared to the MTB strain. Among the synthesized quinolinyl monocarbonyl curcumin analogues, compounds **59a**, **59c**, and **60a**–**c** with MIC^90^ range of 7.8–27.9 μg/mL were active against dormant MTB strains, while compounds **59b**–**d**, **60a**–**c**, and **60d** with MIC_90_ range of 2.7–27.2 μg/mL were active against dormant *M. bovis* BCG strains were active. The biological evaluation results reveal that, the activity was considerably affected by introducing various substituents on the quinoline ring and N-methylation of piperidinone scaffold. From the compounds **59a**–**d**, compound **59a** and **59c** showed moderate antitubercular activity with MIC90 value 25.5 and 27.9 µg/mL, respectively, against dormant MTB strain. The remaining analogues from the series does not display significant antitubercular activity against dormant MTB strain with MIC_90_ values > 30 µg/mL. *N*-Methylpiperidinone-based analogues **60a**–**d**, particularly, compounds **60a** (MIC_90_ value 26.5 µg/mL) and **60b** (MIC_90_ value 20.0 µg/mL), showed good to moderate antitubercular activity against dormant MTB strain. In particular, compound **60c** showed excellent antitubercular activity against dormant MTB strain with MIC90 value 7.8 µg/mL. Hence, among all the synthesized analogues, the only compounds **59a**, **59c**, and **60a**–**c** showed moderate to excellent antitubercular activity against dormant MTB strain. From the analogues **59a**–**d**, compound **59b** showed excellent antitubercular activity with MIC_90_ value 2.7 µg/mL against dormant *M. bovis* BCG strain. Compound **59c** showed moderate antitubercular activity with MIC90 value 27.2 µg/mL against dormant *M. bovis* BCG strain. Compound **59d** showed promising antitubercular activity against dormant *M. bovis* BCG strain with MIC_90_ value 9.2 µg/mL. From the series **60a**–**d**, compound **60a** showed excellent antitubercular activity with a MIC90 value 7.3 µg/mL against dormant *M. bovis* BCG strain. Compound **60b** and **60d** showed good to moderate antitubercular activity with MIC90 value 15.4 and 21.5 µg/mL, respectively, against dormant *M. bovis* BCG strain. Compound **60c** showed promising antitubercular activity against dormant *M. bovis* BCG strain with MIC_90_ value 9.4 µg/mL. In short, quinoline-based monocarbonyl curcumin analogues **59b**–**d**, **60a**–**d** showed good to excellent antitubercular activity against dormant *M. bovis* BCG strain.

Aurone (CF: C_15_H_10_O_2_, MW: 222.24, 2-Benzylidene-3(2*H*)-benzofuranone) is a heterocyclic compound of the flavonoid family with a benzofuran moiety linked to a benzylidene group at position C-2. Campaniço and colleagues [51] synthesized azaaurones derivative **61** (Figure 13) (Table 9) and evaluated its anti-mycobacterial MDR- and XDR-TB activity. Compound **61** showed excellent activity against clinically isolated MDR and XDR-TB with MIC_99_ values of 0.649 and 0.736 μM, respectively.

Kumar and colleagues [52] synthesized aurones quinoline derivatives **62a**–**f** (Figure 13) (Table 9) and evaluated their antibacterial and antifungal activities against *Bacillus subtilis*, *Staphylococcus aureus*, *Klebsiella pneumoniae*, *Aspergillus fumigatus*, *Candida albicans*, and *Fusarium oxysporum*. All test compounds showed antibacterial activity against Gram-positive test strains; however, only compounds **62c** and **62e** showed antibacterial activity against Gram-negative test strains (*Klebsiella pneumoniae*); both MIC values are 0.625 mg/mL. In addition, only compounds **62a**–**b**, **62d**, and **62f** showed activity against the acid-fast microbial strain (MIC range of 0.625–0.078 mg/mL). This different antimicrobial behavior may be due to the differences in their composition and cell membrane structure in different microbial strains since the peptidoglycan layer of Gram-positive bacteria and the phospholipid membrane of Gram-negative bacteria interact differently with different antimicrobial agent molecules.

Sabatini and colleagues [53] synthesized the quinoline derivatives **63**–**64** (Figure 13) (Table 9) from flavonoids and evaluated the function of EPI. The results showed that compounds **63** and **64** exhibited good antibacterial activity (SA-1199B inhibited EtBr efflux > 65% at 50 μM concentration).

Wang and colleagues [54] synthesized 3-(iso)quinolinyl-4-chromenone derivatives **65**–**66** (Figure 13) (Table 9) and evaluated their antifungal activity. Bioassay data showed that 3-quinolinyl-4-chromenone **65** showed significant in vitro activity against *Sclerotinia sclerotiorum*, *Vibrio marcescens*, and grey mould with EC_50_ values of 3.65, 2.61, and 2.32 mg/L, respectively. 3-isoquinolinyl-4-chrome none **66** showed excellent in vitro activity against *Sclerotinia sclerotiorum* with an EC_50_ value of 1.94 mg/L, which was close to the commercial fungicide chlorothalonil (EC_50_ = 1.57 mg/L), but lower than Boscard (EC_50_ = 0.67 mg/L). For *V. mali* and *B. cinerea*, the activity of 3-isoquinolinyl-4-chrome none **66** (EC_50_ = 1.56, 1.54 mg/L) was significantly higher than that of chlorothalonil (EC_50_ = 11.24, 2.92 mg/L). In addition, in vivo experiments showed that compounds **65** and **66** showed 88.24% and 94.12% inhibition against grey mould at 50 mg/L, compared to 76.47% and 97.06% inhibition by the positive controls chlorothalonil and boscalid, respectively. Physiological and biochemical studies suggest that the main mechanism of action of compounds **65** and **66** on *S. sclerotiorum* and *B. cinerea* may involve altering the morphology and increasing the permeability of cell membranes of *A. aurantium*.

**Figure 14:**
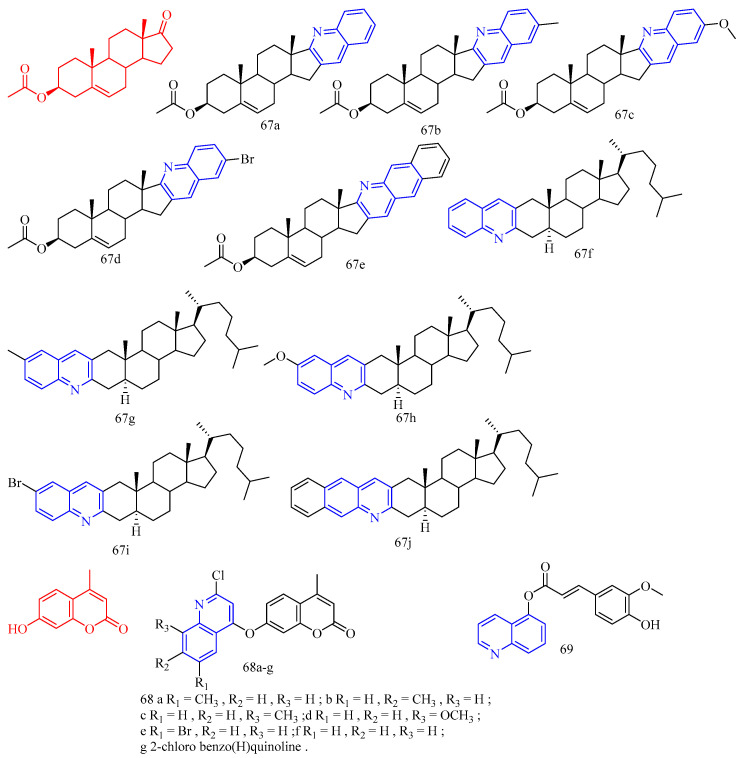
The chemical structures of antibacterial compounds **67**–**69**.

Gogoi and colleagues [55] synthesized the A- and D-ring-fused steroid quinolines derivatives **67a**–**j** (Figure 14) (Table 9) and evaluated their antibacterial and antifungal activities. Cystine was used as a standard drug against fungi, and gentamicin sulfate was used against bacteria. The results showed that compound **67a** did not exert potent inhibitory activity against all bacterial strains tested. Methoxy derivative **67c** showed an inhibitory effect against bacterial strains *Pseudomonas aeruginosa*, while compounds **67e**, **67g**–**h**, and **67j** showed significant inhibition against bacterial strains *Staphylococcus aureus* and *Bacillus subtilis*. These results suggest that the substituents in the quinoline molecules of compounds **67a** and **67f**, as well as the backbone structure, play an important role in the inhibitory activity of bacterial strains. For the fungal strain *A. niger*, only the tested compound **67a** showed a strong inhibition (MIC = 22 μg/mL), while compounds **67e** and **67g**–**h** showed only moderate inhibition. In contrast, most of the tested compounds **67e**–**h** and **67j** showed a strong inhibition of the growth of the fungal strain *C. albicans* (MIC values arranged 13–19 μg/mL). Compounds **67b**, **67d**, and **67i** were not effective against any of the tested strains. Furthermore, the determination of MICs and MFCs of the active compounds showed that compound **67e** showed maximum activity against most of the tested strains with 21 μg/mL for *S. aureus*, 19 μg/mL for *B. subtilis,* 23 μg/mL for *C. albicans* and 28 μg/mL for *C. niger*. It is evident from the data that compounds **67c** and **67f** against *Pseudomonas aeruginosa* showed very good antibacterial activity, almost similar to the standard drug gentamicin sulfate. Similarly, **67e**, **67g**–**h**, and **67j** showed inhibitory activity against the bacterial strains *Bacillus subtilis* (MIC values arranged 12–22 μg/mL) and *Staphylococcus aureus* (MIC values arranged 10–23 μg/mL), indicating that these compounds are promising antimicrobial compounds for further investigation.

Balaji and colleagues [56] synthesized a series of quinoline–coumarin derivatives **68a**–**g** (Figure 14) (Table 9) and evaluated the in vitro antibacterial activity against Gram (+) and Gram (−) bacteria, such as *Escherichia coli*, *Pseudomonas aeruginosa*, *Staphylococcus aureus*, *Meloidogyne litoralis*, and *Bacillus subtilis*. Compounds possessing methyl, methoxy, and fused aryl rings, such as **68c**, **68d**, and **68g**, at C-8 of quinoline ring showed better activity than their standard drug streptomycin against *Escherichia coli* (all MIC values are 6.25 μg/mL). Similarly, compounds **68d** and **68g** showed better activity against *P. aeruginosa* (both MIC values are 6.25 μg/mL). Compound **68f** with bromine at C-6 showed better activity against *M. litoralis* (MIC = 6.25 μg/mL), whereas **68a** with methyl at C-6 showed better activity against *S. aureus* (MIC = 12.5 μg/mL). No compound has good activity against *B. subtilis* with the standard drug streptomycin, respectively. DPPH (1,1-diphenyl-2-picryl-hydrazil) radical scavenging method has been chosen to evaluate the antioxidant potential of the compounds **68a**–**g** compared with that of commercial antioxidant butylated hydroxytoluene (BHT). The results in percentage are expressed as of absorbance decrease at 517 nm, and the absorbance of DPPH solution in the absence of compounds. The values revealed that the radical scavenging activity of 7-(2-chloroquinolin-4-yloxy)-4-methyl-2*H*-hromen-2-one on DPPH radicals increases with the increase in concentration. Compounds possessing chloro, bromo substituents at C-6 (**68e**,**f**), showed maximum activity at a concentration of 1000 μg/mL. The radical scavenging activity of compounds possessing methyl at C-7 (**68b**) exhibited less potent than the standard. In summary, these compounds have been subjected to antimicrobial screening against a panel of human pathogens, and most of them are found to be more active than the standard drugs. In addition, antioxidant activity for compound **68e** shows a moderate 78% of inhibition. The binding energy value of synthesized compounds is less than the standard antimalarial drugs like chloroquine and amodiaquine.

Khatkar and colleagues [57] synthesized a quinoline ferulic acid derivative **69** (Figure 14) (Table 9). The synthesized compound was evaluated in vitro for its antibacterial activity against various Gram-positive and Gram-negative bacterial and fungal strains. As a result, compound **69** was found to be most effective against *B. subtilis* with a pMICbs value of 2.01.

**Figure 15:**
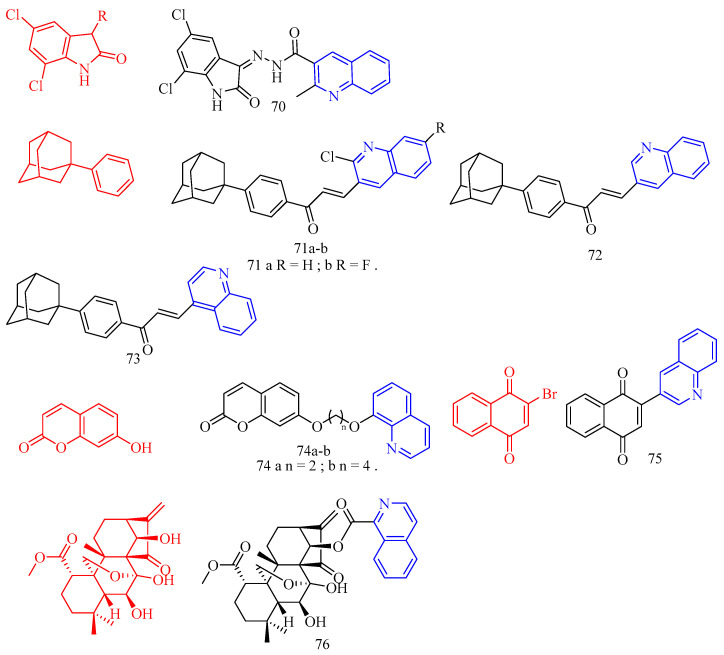
The chemical structures of antibacterial compound **70**–**76**.

Maddela and colleagues [58] designed and synthesized the isatin–quinoline derivative compound **70** (Figure 15) (Table 9) and evaluated its antitubercular activity against *Mycobacterium tuberculosis*. Compound **70** had a MIC of 0.09 mg/L and showed good inhibitory activity compared to the standard drug isoniazid.

Tabbi and colleagues [59] synthesized new adamantane-containing chalcones derivatives **71**–**73** (Figure 15) (Table 9) and evaluated their resistance against *Enterococcus faecalis* 29212, *Pseudomonas aeruginosa* ATCC 27853, strong antibacterial activity against Escherichia coli and interesting antifungal activity against *Candida albicans* ATCC 90030. Compounds **71**–**73** were also tested for anti-*Candida* activity. All compounds were found to have the same activity as ketoconazole against *C. glabrata* ATCC 90030 (MIC = 200 μg/mL). However, the compounds had no significant anti-*Candida* activity against *C. krusei* ATCC 6258 (MIC = 100 μg/mL).

Pan and colleagues [60] synthesized hydroxycoumarin quinoline derivatives **74a**–**b** (Figure 15) (Table 9) to evaluate their antifungal activity. Compound **74b** exhibited potent growth inhibition against all tested fungi. (The inhibition rates of compound **74b** against *A. alternate*, *A. solani*, *B. cinerea*, and *F.oxysporum* were 57.6%, 79.0%, 72.9%, and 89.6%, respectively). Compound **74a** exhibited moderate activity. The inhibition rates of compound **74a** against *A. alternate*, *A. solani*, *B. cinerea*, and *F. oxysporum* were 49.3%, 61.2%, 63.3%, and 56.4%, respectively. In contrast, compound **74b** exhibited selective antifungal activity against *A. alternata* and *A. solani*, which were alternatively present.

Naphthoquinone (CF: C_10_H_6_O_2_, MW: 158.15, Naphthalene-1,4-dione) is an organic substance, and theoretically, there are 6 kinds of naphthoquinone, of which only 1,4-, 1,2-, and 2,6- can be obtained stably. 1,4-Naphthoquinone, also known as α-naphthoquinone, is used to make dyes, medicines, and fungicides. Kalt and colleagues [61] synthesized 1,4-naphthoquinonequinoline derivative **75** (Figure 15) (Table 9) and tested its anti-mycobacterial activity. The results showed that compound **75** had a slight inhibitory effect against *mycobacteria* with a MIC of 8 μg/mL.

Lasiokaurin (molecular formula: C_22_H_30_O_7_, MW: 406.47, (1α,6β,7α,14*R*)-1-(Acetyloxy)-7,20-epoxy-6,7,14-trihydroxykaur-16-en-15-one) is a diterpene compound present in the leaves of the Lamiaceae plant, Isodon trichocarpus Kudo, and the leaves of *Rabdosia japonica* (Burm.f.) Hara. Li and colleagues [62] synthesized quinoline-based lasiokaurin derivative **76** (Figure 15) (Table 9) and performed antibacterial tests. The results showed that compound **76** exhibited the most promising antibacterial activity with MICs of 2.0 and 1.0 μg/mL against the Gram-positive bacteria *Staphylococcus aureus* and *Bacillus subtilis*, respectively.

**Table 9:** Quinoline derivatives with antibacterial activity.

Compd.	Activity	Origin	Ref
**56a**	*E. coli* MIC = 25.0 μg/mL *S. aureus* MIC = 12.5 μg/mL *C. albicans* MIC = 12.5 μg/mL	synthetic	[48]
**56b**	*E. coli* MIC = 12.5 μg/mL *S. aureus* MIC = 25.0 μg/mL *C. albicans* MIC = 25.0 μg/mL	synthetic	[48]
**56c**	*E. coli* MIC = 6.25 μg/mL *S. aureus* MIC = 12.5 μg/mL *C. albicans* MIC = 12.5 μg/mL	synthetic	[48]
**56d**	*E. coli* MIC = 12.5 μg/mL *S. aureus* MIC = 12.5 μg/mL *C. albicans* MIC = 25.0 μg/mL	synthetic	[48]
**56e**	*E. coli* MIC = 6.25 μg/mL *S. aureus* MIC = 12.5 μg/mL *C. albicans* MIC = 12.5 μg/mL	synthetic	[48]
**56f**	*E. coli* MIC = 12.5 μg/mL *S. aureus* MIC = 12.5 μg/mL *C. albicans* MIC = 12.5 μg/mL	synthetic	[48]
**56g**	*E. coli* MIC = 12.5 μg/mL *S. aureus* MIC = 12.5 μg/mL *C. albicans* MIC = 12.5 μg/mL	synthetic	[48]
**56h**	*E. coli* MIC = 12.5 μg/mL *S. aureus* MIC = 25.0 μg/mL *C. albicans* MIC = 25.0 μg/mL	synthetic	[48]
**57a**	*E. coli* MIC = 12.5 μg/mL *S. aureus* MIC = 12.5 μg/mL *C. albicans* MIC = 12.5 μg/mL	synthetic	[48]
**57b**	*E. coli* MIC = 12.5 μg/mL *S. aureus* MIC = 25.0 μg/mL *C. albicans* MIC = 12.5 μg/mL	synthetic	[48]
**58** **a**	*B.s.* MIC = 500 μg/mL *S.a.* MIC = 250 μg/mL *E.c.* MIC = 100 μg/mL *S.t.* MIC = 100 μg/mL *A.n.* MIC = 500 μg/mL *C.a.* MIC = 500 μg/mL	synthetic	[49]
**58b**	*B.s.* MIC = 250 μg/mL *S.a.* MIC = 200 μg/mL *E.c.* MIC = 500 μg/mL *S.t.* MIC = 250 μg/mL *A.n.* MIC = 200 μg/mL *C.a.* MIC = 500 μg/mL	synthetic	[49]
**58c**	*B.s.* MIC = 200 μg/mL *S.a.* MIC = 250 μg/mL *E.c.* MIC = 62.5 μg/mL *S.t.* MIC = 100 μg/mL *A.n.* MIC = 250 μg/mL *C.a.* MIC = 250 μg/mL	synthetic	[49]
**58d**	*B.s.* MIC = 250 μg/mL *S.a.* MIC = 125 μg/mL *E.c.* MIC = 62.5 μg/mL *S.t.* MIC = 100 μg/mL *A.n.* MIC = 500 μg/mL *C.a.* MIC = 1000 μg/mL	synthetic	[49]
**58e**	*B.s.* MIC = 250 μg/mL *S.a.* MIC = 125 μg/mL *E.c.* MIC = 200 μg/mL *S.t.* MIC = 250 μg/mL *A.n.* MIC = 500 μg/mL *C.a.* MIC = 1000 μg/mL	synthetic	[49]
**58f**	*B.s.* MIC = 250 μg/mL *S.a.* MIC = 500 μg/mL *E.c.* MIC = 100 μg/mL *S.t.* MIC = 62.5 μg/mL *A.n.* MIC = 1000 μg/mL *C.a.* MIC = 250 μg/mL	synthetic	[49]
**58g**	*B.s.* MIC = 250 μg/mL *S.a.* MIC = 200 μg/mL *E.c.* MIC = 100 μg/mL *S.t.* MIC = 200 μg/mL *A.n.* MIC = 1000 μg/mL *C.a.* MIC = 200 μg/mL	synthetic	[49]
**58h**	*B.s.* MIC = 200 μg/mL *S.a.* MIC = 250 μg/mL *E.c.* MIC = 100 μg/mL *S.t.* MIC = 200 μg/mL *A.n.* MIC = 500 μg/mL *C.a.* MIC = 250 μg/mL	synthetic	[49]
**58i**	*B.s.* MIC = 500 μg/mL *S.a.* MIC = 250 μg/mL *E.c.* MIC = 250 μg/mL *S.t.* MIC = 250 μg/mL *A.n.* MIC = 1000 μg/mL *C.a.* MIC = 200 μg/mL	synthetic	[49]
**58j**	*B.s.* MIC = 500 μg/mL *S.a.* MIC = 100 μg/mL *E.c.* MIC = 62.5 μg/mL *S.t.* MIC = 100 μg/mL *A.n.* MIC = 1000 μg/mL *C.a.* MIC > 1000 μg/mL	synthetic	[49]
**58k**	*B.s.* MIC = 200 μg/mL *S.a.* MIC = 125 μg/mL *E.c.* MIC = 100 μg/mL *S.t.* MIC = 200 μg/mL *A.n.* MIC = 1000 μg/mL *C.a.* MIC = 1000 μg/mL	synthetic	[49]
**58l**	*B.s.* MIC = 250 μg/mL *S.a.* MIC = 100 μg/mL *E.c.* MIC = 200 μg/mL *S.t.* MIC = 250 μg/mL *A.n.* MIC > 1000 μg/mL *C.a.* MIC = 1000 μg/mL	synthetic	[49]
**59a**	MTB MIC_50_ = 8.7 μg/mL MIC_90_ = 25.5 μg/mL *M. Bovis* BCG MIC_50_ = 12.9 μg/mL MIC_90_ > 30 μg/mL	synthetic	[50]
**59b**	MTB MIC_50_ > 30 μg/mL MIC_90_ > 30 μg/mL *M. Bovis* BCG MIC_50_ = 2.5 μg/mL MIC_90_ > 30 μg/mL	synthetic	[50]
**59c**	MTB MIC_50_ = 2.8 μg/mL MIC_90_ > 30 μg/mL *M. Bovis* BCG MIC_50_ = 1.4 μg/mL MIC_90_ = 2.7 μg/mL	synthetic	[50]
**59d**	MTB MIC_50_ = 27.8 μg/mL MIC_90_ > 30 μg/mL *M. Bovis* BCG MIC_50_ = 19.7 μg/mL MIC_90_ > 30 μg/mL	synthetic	[50]
**60a**	MTB MIC_50_ = 6.7 μg/mL MIC_90_ = 26.5 μg/mL *M. Bovis* BCG MIC_50_ = 6.0 μg/mL MIC_90_ = 7.3 μg/mL	synthetic	[50]
**60b**	MTB MIC_50_ = 7.7 μg/mL MIC_90_ = 20.0 μg/mL *M. Bovis* BCG MIC_50_ = 5.8 μg/mL MIC_90_ = 15.4 μg/mL	synthetic	[50]
**60c**	MTB MIC_50_ = 2.3 μg/mL MIC_90_ = 7.8 μg/mL *M. Bovis* BCG MIC_50_ = 5.8 μg/mL MIC_90_ = 9.4 μg/mL	synthetic	[50]
**60d**	MTB MIC_50_ > 30 μg/mL MIC_90_ > 30 μg/mL *M. Bovis* BCG MIC_50_ = 3.3 μg/mL MIC_90_ = 21.5 μg/mL	synthetic	[50]
**61**	MDR MIC_99_ = 0.649 μM XDR-TB MIC_99_ = 0.736 μM	synthetic	[51]
**62a**	*B. subtilis* MIC = 0.020 mg/mL *S. aureus* MIC = 1.25 mg/mL *M. smegmatis* MIC = 0.625 mg/mL *F. oxysporum* MIC = 0.625 mg/mL	synthetic	[52]
**62b**	*B. subtilis* MIC = 1.25 mg/mL *S. aureus* MIC = 2.5 mg/mL	synthetic	[52]
**62c**	*B. subtilis* MIC = 1.25 mg/mL *S. aureus* MIC = 1.25 mg/mL *K. pneumoniae* MIC = 0.625 mg/mL	synthetic	[52]
**62d**	*B. subtilis* MIC = 1.25 mg/mL *S. aureus* MIC = 1.25 mg/mL *M. smegmatis* MIC = 0.625 mg/mL	synthetic	[52]
**62e**	*B. subtilis* MIC = 1.25 mg/mL *S. aureus* MIC = 1.25 mg/mL *M. smegmatis* MIC = 0.625 mg/mL *C. albicans* MIC = 0.156 mg/mL *Klebsiella pneumoniae* MIC = 0.625 mg/mL	synthetic	[52]
**62f**	*B. subtilis* MIC = 1.25 mg/mL *S. aureus* MIC = 1.25 mg/mL *M. smegmatis* MIC = 0.625 mg/mL	synthetic	[52]
**63**	inhibited EtBr efflux > 65% at 50 μM	synthetic	[53]
**64**	inhibited EtBr efflux > 65% at 50 μM	synthetic	[53]
**65**	*Sclerotinia sclerotiorum* EC_50_ = 1.57 mg/L	synthetic	[54]
**66**	*V. mali* EC_50_ = 1.56 mg/L *B. cinerea* EC_50_ = 1.54 mg/L	synthetic	[54]
**67a**	-	synthetic	[55]
**67b**	-	synthetic	[55]
**67c**	*Pseudomonas aeruginosa* MIC = 18 μg/mL	synthetic	[55]
**67d**	-	synthetic	[55]
**67e**	*Staphylococcus aureus* MIC = 21 μg/mL *Bacillus subtilis* MIC = 19 μg/mL	synthetic	[55]
**67f**	*Pseudomonas aeruginosa* MIC = 19 μg/mL	synthetic	[55]
**67g**	*Staphylococcus aureus* MIC = 28 μg/mL *Bacillus subtilis* MIC = 26 μg/mL	synthetic	[55]
**67h**	*Staphylococcus aureus* MIC = 40 μg/mL *Bacillus subtilis* MIC = 24 μg/mL	synthetic	[55]
**67i**	-	synthetic	[55]
**67j**	*StaphylococcusAureus* MIC = 24 μg/mL *Bacillus subtilis* MIC = 40 μg/mL	synthetic	[55]
**68a**	*Escherichia coli* MIC = 12.5 μg/mL *Pseudomonas aeruginosa* MIC = 100 μg/mL *Staphylococcus aureus* MIC = 12.5 μg/mL	synthetic	[56]
**68b**	*Pseudomonas aeruginosa* MIC = 12.5 μg/mL	synthetic	[56]
**68c**	*Escherichia coli* MIC = 6.25 μg/mL *Meloidogyne litoralis* MIC = 100 μg/mL	synthetic	[56]
**68d**	*Escherichia coli* MIC = 6.25 μg/mL *Pseudomonas aeruginosa* MIC = 6.25 μg/mL *Bacillus subtilis* MIC = 100 μg/mL	synthetic	[56]
**68e**	*Escherichia coli* MIC = 100 μg/mL *Pseudomonas aeruginosa* MIC = 12.5 μg/mL *Meloidogyne litoralis* MIC = 100 μg/mL *Staphylococcus aureus* MIC = 50 μg/mL	synthetic	[56]
**68f**	*Pseudomonas aeruginosa* MIC = 50 μg/mL *Meloidogyne litoralis* MIC = 6.25 μg/mL	synthetic	[56]
**68g**	*Escherichia coli* MIC = 6.25 μg/mL *Pseudomonas aeruginosa* MIC = 6.25 μg/mL *Staphylococcus aureus* MIC = 50 μg/mL	synthetic	[56]
**69**	*B. subtilis* pMICbs = 2.01	synthetic	[57]
**70**	*Mycobacterium tuberculosis* MIC = 0.09 mg/L	synthetic	[58]
**71a**	*C. glabrata* ATCC 90030 MIC = 200 μg/mL	synthetic	[59]
**71b**	*C. glabrata* ATCC 90030 MIC = 200 μg/mL	synthetic	[59]
**72**	*C. glabrata* ATCC 90030 MIC = 200 μg/mL	synthetic	[59]
**73**	*C. glabrata* ATCC 90030 MIC = 200 μg/mL	synthetic	[59]
**74a**	*A. alternate* inhibition rate = 49.3% *A. solani* inhibition rate = 61.2% *B. cinerea* inhibition rate = 63.3% *F. oxysporum* inhibition rate = 56.4%	synthetic	[60]
**74b**	*A. alternate* inhibition rate = 57.6% *A. solani* inhibition rate = 79.0% *B. cinerea* inhibition rate = 72.9% *F. oxysporum* inhibition rate = 89.6%	synthetic	[60]
**75**	*Mycobacteria* MIC = 8 μg/mL	synthetic	[61]
**76**	*Staphylococcus aureus* MIC = 2.0 μg/mL *Bacillus subtilis* MIC = 1.0 μg/mL	synthetic	[62]

Summary: Quinoline-based molecules have been found to be very effective in inhibiting microbial pathogens. Among the drugs of quinoline scaffolds, fluoroquinolone antibiotics, represented by ciprofloxacin, are a large class of antibiotics. In addition, bedaquiline is a diarylquinoline-based drug that has been used to treat multidrug-resistant tuberculosis (MDR-TB). In order to adapt to environmental changes, especially the use of antibiotics, bacteria have developed a variety of mechanisms to resist various adverse conditions. Therefore, bacterial infection has once again evolved into a serious threat worldwide. The increasing number of multidrug-resistant microbial strains and new advances in untreatable infections make the treatment of bacterial infections difficult.

Compound **62e** showed excellent antitubercular activity with MIC value 0.156 µg/mL against dormant *C. albicans strain.* Compound **70** against *Mycobacterium tuberculosis* showed the strongest inhibitory activity with a MIC value of 0.09 μg/mL. Compound **76** exhibited the most promising antibacterial activity with MICs of 2.0 and 1.0 μg/mL against the Gram-positive *Bacteria Staphylococcus aureus* and *Bacillus subtilis*, respectively.

These results suggest that the introduction of quinoline on the basis of aurone, isatin, and lasiokaurin can enhance antibacterial activity. However, there is no obvious rule between the introduced sites and the substituents on quinoline; moreover, the antibacterial mechanism of these compounds has not been studied.

### 3.10. Anticancer Activity

Lombard and colleagues [39] synthesized two quinoline–coumarin derivatives **43**–**44** (Figure 11) (Table 10) and evaluated their anticancer activity against kidney cancer (TK10), melanoma (UACC62), and breast cancer (MCF7) cell lines, etoposide was used as a positive control. The results of the five-dose cancer screening showed that mixed dimer **44** was less active, and its anticancer activity was classified as against renal (TGI = 18.5 μM) and melanoma (TGI = 17.43 μM) cell linesmoderate. The breast (MCF7) cell line showed higher sensitivity to dimer **44** with a TGI of 2.92 μM, so the activity of dimer **44** could be classified as potent for this cell line. Dimer **44** was 2-fold (TGI, 18.5 vs. 43.33 μM), 15-fold (TGI, 2.92 vs. 43.52 μM), and 5.7-fold (TGI, 17.43 vs. >100 μM) activity than etoposide against TK10, UACC62, and MCF7, respectively. The synthetic dimer exhibited moderate to potent anticancer activity against the cell lines studied and inhibited the growth of all three cell lines at very low concentrations (GI_50_ values in the range of 0.03–0.08 μM). Compound **43** was able to inhibit the growth of all three cell lines at 10 μM, while compound **44** could inhibit the growth of the UACC62 cell line and the other two cell lines at 100 μM only at 10 μM. Very low LC_50_ and LC_100_ values were obtained for both compounds; moreover, these two compounds have very low toxicity to normal cells.

Tabbi and colleagues [59] synthesized the adamantane chalcone–quinoline derivatives **71a**–**b** (Figure 15) (Table 10) and evaluated their in vitro anticancer activity against human pancreatic cancer cells Mia Paka 2. The growth inhibitory activities of compounds **71a** and **71b** were 85% and 77%. Thus, compounds **71a**–**b** possess some anticancer activity.

**Figure 16:**
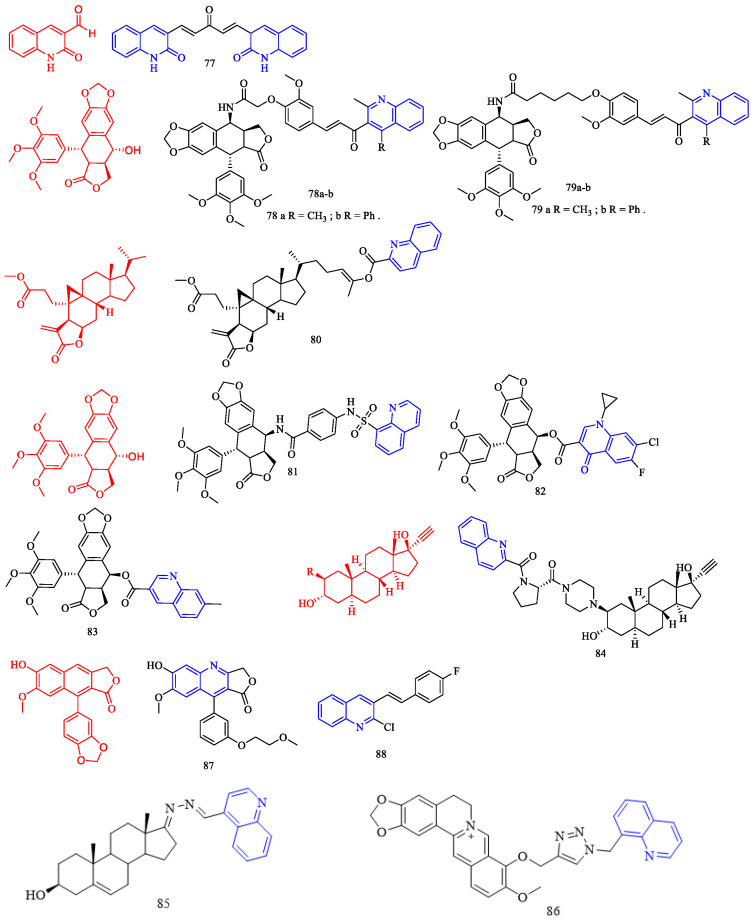
The chemical structures of anticancer compounds **77**–**88**.

Abonia and colleagues [63] synthesized novel quinoline-2-ketochalcone derivative **77** (Figure 16) (Table 10). In vitro antitumor assays showed that compound **77** exhibited high activity in the samples selected and evaluated by NCI. In particular, compound **77** showed the most significant activity against 50 human tumor cell lines with GI_50_ values of 1.0 μM, with HCT-116 (colon, GI_50_ = 0.131 μM) and LOX IMVI (melanoma, GI_50_ = 0.134 μM) being the most susceptible strains.

Podophyllotoxin (CF: C_22_H_22_O_8_, MW: 414.41, 1,3,3a,4,9,9a-hexahydro-9-hydroxy-6,7-(methylenedioxy)-4-(3′,4′,5′-trimethoxyphenyl)benz[f]isobenzofuran-3-one), also known as podafilol, is a non-alkaloid lignin-like toxin. Kamal and colleagues [64] synthesized the onychotoxin–quinoline derivatives **78a**–**b** and **79a**–**b** (Figure 16) (Table 10) and evaluated their higher activity against A549, A375, MCF-7, HT-29, and ACHN, and the positive control drugs etoposide and doxorubicin than they themselves. The IC_50_ values of compound **78a** against A549, A375, MCF-7, HT-29, and ACHN were 15.4 μM, 14.5 μM, 13.8 μM, 12.3 μM, and 10.8 μM, respectively. The IC_50_ values of compound **78b** against A549, A375, MCF-7, HT-29, and ACHN were 13.4 μM, 7.7 μM, 11.2 μM, 7.75 μM, and 15.7 μM, respectively. The IC_50_ values of compound **79a** against A549, A375, MCF-7, HT-29, and ACHN were 10.6 μM, 10.3 μM, 8.6 μM, 11.8 μM, and 10.7 μM, respectively. The IC_50_ values of compound **79b** against A549, A375, MCF-7, HT-29, and ACHN were 7.7 μM, 6.8 μM, 2.2 μM, 8.9 μM, and 9.46 μM, respectively.

Ring-A 3,4-seco-cycloartane-type triterpenes (CF: C_30_H_44_O_2_, MW: 468.67, (3*R*,3a*R*,5a*S*,6a*R*,6b*R*,9a*R*,10a*S*,10b*S*)-3-[(1*R*)-1,5-Dimethyl-4-hexen-1yl]tetradecahydro-3a) mainly exist in the gardenia plants of *Rubi ceae*. Pudhom and colleagues [65] synthesized 3,4-eco-cycloartane-type triterpene quinoline derivative **80** (Figure 16) (Table 10) and evaluated the effect on angiogenesis. The inhibition rate of compound **80** ranged between 50 and 60%.

Kamal and colleagues [66] synthesized 4b-sulfonamide and 4b-[(40-sulfonamide)benzamide] conjugates of podophyllotoxin **81** (Figure 16) (Table 10) and evaluated its effect on anticancer activity. The results showed that the inhibition A549 activity of compound **81** was more potent than that of the positive control drugs doxorubicin and etoposide (the GI_50_ of compound **81** was 2.51 μM).

Zhao and colleagues [67] synthesized 4′-demethylghostatin (DMEP) quinoline derivatives **82**–**83** (Figure 16) (Table 10) and evaluated their anticancer activity. The inhibitory activity of compound **82** on HepG2, HeLa, A549, and BGC-823 was stronger than that of itself and the positive control etoposide. The IC_50_ values of compound **82** against HepG2, HeLa, A549, BGC-823, and HL-7702 were 16.03 μM, 0.60 μM, 10.05 μM, 17.41 μM, and 41.77 μM, respectively. The inhibitory activity of compound **83** on HepG2 and A549 is stronger than that of itself and the positive control. The inhibitory activity of compound **83** on HeLa and BGC-823 is not as good as its own. The IC_50_ values of compound 90 against HepG2, HeLa, A549, BGC-823, and HL-7702 were 9.18 μM, 20.53 μM, 19.20 μM, 28.81 μM, and 20.09 μM, respectively. Compounds **82**–**83** are both on HL-7702 was more toxic than itself.

Ayan and colleagues [68] synthesized a derivative of the aminosteroidal E-37P-quinoline derivative **84** (Figure 16) (Table 10) and evaluated their anticancer activity. The results showed that compound **84**, a 5a-androstane-3a,17b-diol derivative with a quinoline nucleus at the end of the piperazine-proline side-chain at position 2b and an ethinyl at position 17a, showed very good antiproliferative activity among the five cancer cell lines studied. The IC_50_ values of compound **84** for HL-60, MCF-7, T-47D, LNCaP, and WEHI-3 were 0.1, 0.1, 0.1, 2.0, and 1.1 μM, respectively. Furthermore, compound **84** weakly inhibited the two representative liver enzymes, CYP3A4 and CYP2D6, indicating a low risk of drug-drug interactions.

Cui and colleagues [69] synthesized quinoline-like estrone-17-hydrazone **85** (Figure 16) (Table 10) and assayed its activities against the proliferation of HeLa, HT-29, Bel 7404, and SGC 7901, respectively. The results showed that compound **85** had a quinoline structure on the side chain of 17 and showed a better effect. The antiproliferative activity against test cells in vitro was higher than that of the positive control drug cisplatin. In particular, compound **85** showed excellent antiproliferative activity against SGC 7901 in vitro with an IC_50_ value of 1 μM.

Berberine (CF: C_20_H_18_NO_4_^+^, MW: 336.36, 16,17-dimethoxy-5,7-dioxa-13-azoniapentacyclo [11.8.0.0^2,10^.0^4,8^.0^15,20^] henicosa-1(13),2,4(8),9,14,16,18,20-octaene), a quaternary alkaloid isolated from the traditional Chinese medicine Coptis chinensis, is the main active ingredient in the antibacterial activity of Coptis. Jin and colleagues [70] synthesized quinoline berberine derivative **86** (Figure 16) (Table 10) and determined its antiproliferative activity against MCF-7, MCF-7/ADR, SW-1990 and SMMC-7721, and non-cancerous HUVEC cells. The results showed that the antiproliferative activity of compound **86** against four human cancer cell lines was slightly weaker (The IC_50_ values of compound **86** for MCF-7, MCF-7/ADR, SW1990, and SMMC-7721 were 181.478 μM, 96.523 μM, 111.837 μM, and 75.546 μM, respectively), only inhibited MCF-7 and SMMC-7721 better than itself.

Hayat and colleagues [71] synthesized 4-azanonaphtholide quinoline derivative **87** (Figure 16) (Table 10) and evaluated its antiproliferative activity against five representative cancer cell lines HepG2, A431, A549, MCF 7, and HCT 116. Daurinol served as a positive control. Compound **87** showed almost equivalent activity to daurinol at 10 μM. The IC_50_ values of compound **87** for HepG2, A431, A549, MCF 7, and HCT 116 were 8.40 μM, 11.56 μM, 4.33 μM, 5.99 μM, and 3.48 μM, respectively, exhibited a slightly stronger activity.

Srivastava and colleagues [72] synthesized quinoline–stilbene derivative **88** (Figure 16) (Table 10) and studied it for antiproliferative activity. Compound **88** showed surprisingly strong activity against MDA-MB 468 breast cancer cells (IC_50_ = 0.12 μM).

**Figure 17:**
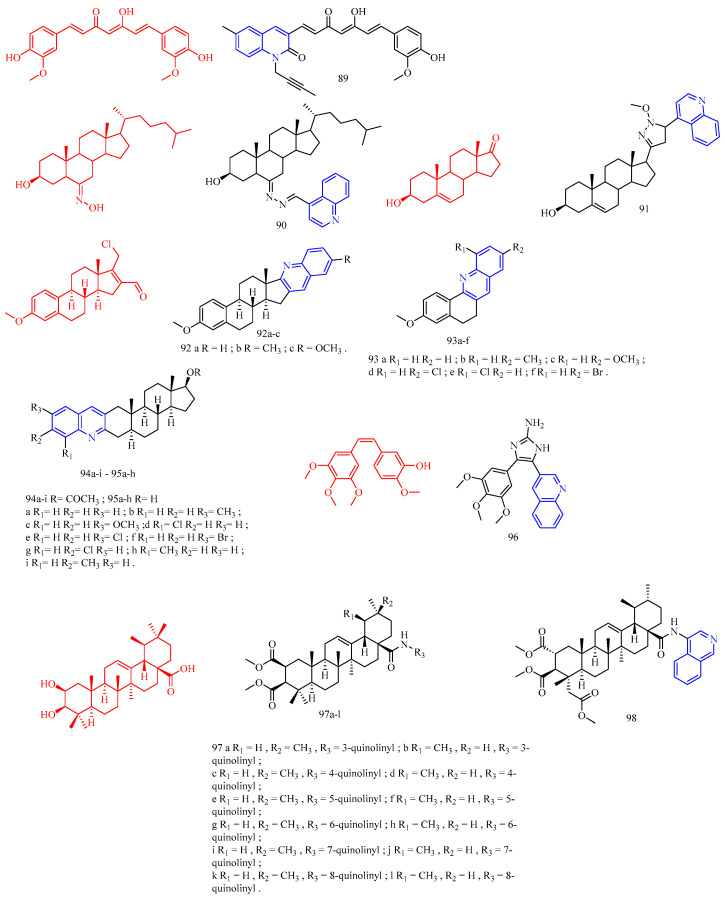
The chemical structures of anticancer compounds **89**–**98**.

Raghavan and colleagues [73] synthesized curcumin–quinolone derivative **89** (Figure 17) and performed in vitro cytotoxicity assays against A549, MCF7, SKOV3, and H460. Compound 115 showed the greatest activity against SKOV3 cells with a minimum IC_50_ value of 12.8 μM observed and was therefore used for further biological experiments. At the IC_50_ concentration, the compound was not toxic to the normal fibroblast cell line NIH3T3, with a cell survival rate of 74.5%.

Cui and colleagues [74] synthesized a steroidal quinoline derivative **90** (Figure 17) (Table 10) with a cholestane type 17-branched structure and determined its inhibitory effect in human hepatoma cells (Bel-7404) and gastric cancer cells (SGC-7901). Compound **90** showed significant growth and proliferation inhibition in both tumor cells, and both were stronger than the positive control cisplatin (IC_50_ values of **90** were 28.7, 17.9 µmol/L).

He and colleagues [75] synthesized quinoline pregnenolone derivative **91** (Figure 17) (Table 10) and the inhibitory activity against human colon cancer cells (HT-29), human cervical cancer cells (HeLa) and human gastric cancer cells (SGC-7901), using cisplatin as a positive control, showed that compound **91** was superior to the inhibitory activity of the positive control substance cisplatin; the inhibitory activity against HeLa and SGC-7901 cells was less than 10 μmol/L (compound **91**’s IC_50_ values were 14.1, 9.1, 8.2 μmol/L).

Baji and colleagues [76] synthesized novel D- and A-ring-fused quinolines of the estrone and 5α-androstanes series **92**–**95** (Figure 17) (Table 10) and investigated their antiproliferative activity on human cervical cancer (C33A, HeLa, and SiHA) and breast cancer (MCF-7, MDA-MB-231, MDA-MB-361, and T47D) cell lines. The results indicated that ring D-fused quinolines **92a**–**c** exhibited weak or modest antiproliferative properties, typically eliciting 30–50% growth inhibition at 30 µM. The cytostatic activities of benz[c]acridine derivatives **93a**–**f** were even weaker, except for **93e** and **93f**, which blocked the proliferation of HeLa cells selectively with IC_50_ values comparable to that of the reference agent cisplatin. IC_50_ values of **93e**–**f** were 99.6 and 89.4 µM, respectively. Ring A-fused quinolines generally inhibited cellular growth more efficiently. Compounds with a 17-OH group (**95a**–**c** and **95e**–**g**) tended to display more pronounced action than the corresponding 17-OAc analogues (**94a**–**c** and **94e**–**g**). Since the efficacy of analogue **95a** containing an unsubstituted quinoline moiety was similar to those of **95b**–**h**, the character of the substituent on the quinoline does not seem to be crucial for the antiproliferative actions; however, substitution at position 6′ (**95c**, **95e**, and **95f**) appeared favorable. The efficacy of **95c** against T47D cells was comparable to that of the reference agent cisplatin. IC_50_ value of **95c** was 80.4 µM.

Combretastatin A-4 (CA-4, CF: C_18_H_20_O_5_, MW: 316.3484, (*Z*)-2-Methoxy-5-(3,4,5-trimethoxystyrene)phenol) is a novel vasopressor that targets tubulin in vivo, inhibiting its polymerization and further selectively destroying the vascular endothelium of tumor tissues, closing the vasculature of tumor tissues and rendering them hypoxic and nutritious, thus acting as an antitumor agent. Chaudhary and colleagues [77] synthesized combretastatin A-4 quinoline derivative **96** (Figure 17) (Table 10) and evaluated its antiproliferative activity. The results showed that compound **96** had higher antiproliferative activity than CA-4. In addition, compound **96** inhibited the migration of highly metastatic MDA-MB-231 more strongly than CA-4, indicating its potent anti-metastatic potential. Compound **96** inhibited the rate and extent of in vitro assembly of purified tubulin with an IC_50_ of 1.6 μM and a dissociation constant of 1.9 μM.

Maslinic acid (CF: C_30_H_48_O_4_, MW: 472.71, (2*α*,3*β*)-2,3-dihydroxyolean-12-en-28-acid) is a pentacyclic triterpene acid, found in hawthorn, red dates, loquat leaves, and olive oil. Madecassic acid (CF: C_30_H_48_O_6_, MW: 504.70, (2*α*,3*β*,4*α*,6*β*)-2,3,6,23-Tetrahydroxyurs-12-en-28-oic acid) is derived from the whole grass of Centella Asiatica of the Umbelliferae. Sommerwerk and colleagues [78] synthesized maslinic acid derivative quinoline derivative **97a**–**l** (Figure 17) (Table 10) and madecassic acid quinoline derivative **98** and evaluated their antitumor activity. Although the cytotoxicity of compounds **97a**–**d** and **97g**–**j** was similar to that of pyridyl-substituted amides, the 8-quinolinyl derivatives **97k** and **97l** exhibited selective cytotoxicity against different human tumor cell lines; however, their overall cytotoxicity was low, and their solubility in water was poor. However, the 5-quinolinyl-substituted compounds **97e** and **97f** performed better, as their EC_50_ values were quite low, and they were cytotoxic against human tumor cell lines but significantly less cytotoxic against non-malignant mouse fibroblasts. Compound **98** has an isoquinoline group present, which shows both low EC_50_ values (EC_50_ = 80 μM for A2780) and high tumor/non-malignant cell selectivity (EC_50_ = 3.23 μM for NIH 3T3, resulting in a selectivity index of 40).

**Figure 18:**
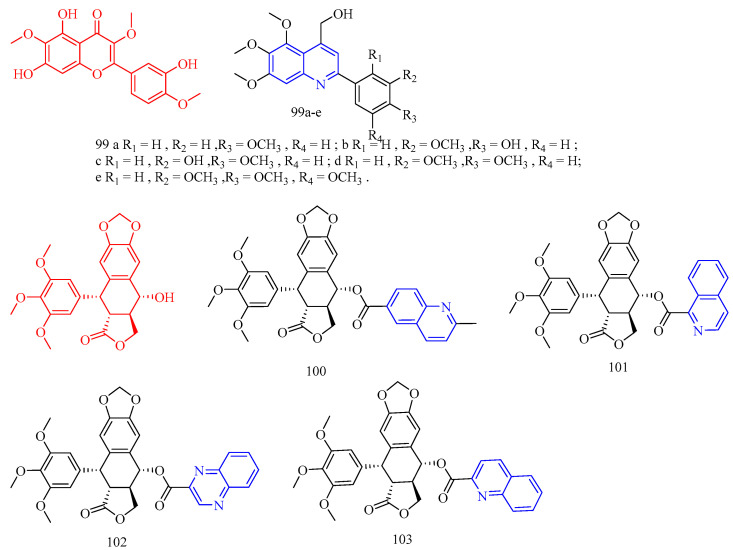
The chemical structures of anticancer compounds **99**–**103**.

Shobeiri and colleagues [79] synthesized 2-aryl-trimethoxyquinoline derivatives **99a**–**e** (Figure 18) (Table 10) and evaluated the cytotoxic activity of the synthesized compounds against four human cancer cell lines MCF-7, MCF-7/MX, A-2780, and A-2780/RCIS. The results showed that all the alcohol derivatives **99a**–**e** showed greater cytotoxicity against the A-2780 cell line compared to the other three cell lines IC_50_ ranging from 7.98 to 60 μM. Interestingly, drug-resistant human breast cancer cells (MCF-7/MX) were more sensitive to all alcohol derivatives except **99a** than the parental cells (MCF-7). In contrast, they induced more cytotoxicity in the A-2780 cell line compared to resistant human ovarian cancer (A-2780/RCIS), suggesting that compounds may exert their cytotoxic activity in different tumor cell types through different mechanisms. Among these quinolines, compound **99e**, which possesses a trimethoxyphenyl group at the second position of the quinoline ring, exhibited the strongest cytotoxicity against cancer cell lines, with the same effect on both parental and resistant cell lines.

Zhang and colleagues [80] synthesized the podophyllotoxin quinoline derivatives **100**–**102** (Figure 18) (Table 10) and evaluated their antiproliferative activity against human leukemia cells (K562 and K562/ADR). Etoposide and doxorubicin were used as positive compounds. Compounds **100**–**102** showed potent cytotoxicity comparable to or higher than etoposide and doxorubicin (IC_50_ values for the antiproliferative activity of compound **100** were 0.061 and 0.064 μM for K562 and K562/ADR cells, respectively. Compound **101** for K562 and K562/ADR cells. IC_50_ values of 0.177 and 0.064 μM for antiproliferative activity and 0.034 and 0.022 μM for compound **102** on K562 and K562/ADR cells, respectively. In general, the activity of the tested molecules was higher against K562 cells than against K562/ADR cells. Moreover, the IC_50_ value of compound **102** in K562/ADR cells was 0.034 μM; its activity was 65.029 and 552.323 times higher than that of etoposide and doxorubicin, respectively.

Li and colleagues [81] synthesized a podophyllotoxin-derived quinoline derivative **103** (Figure 18) (Table 10) and evaluated its antiproliferative activity against human promyelocytic leukemia cells HL60, human gastric cancer cells SGC-7901, human colon cancer cells MCF-7, human in vitro anticancer active breast cancer cells HCT116, and human non-small cell lung cancer cells A549. Unfortunately, the anticancer activity of compound **103** was inferior to that of the positive control drug etoposide (IC_50_ values arranged 8.09–73.40 μM).

**Figure 19:**
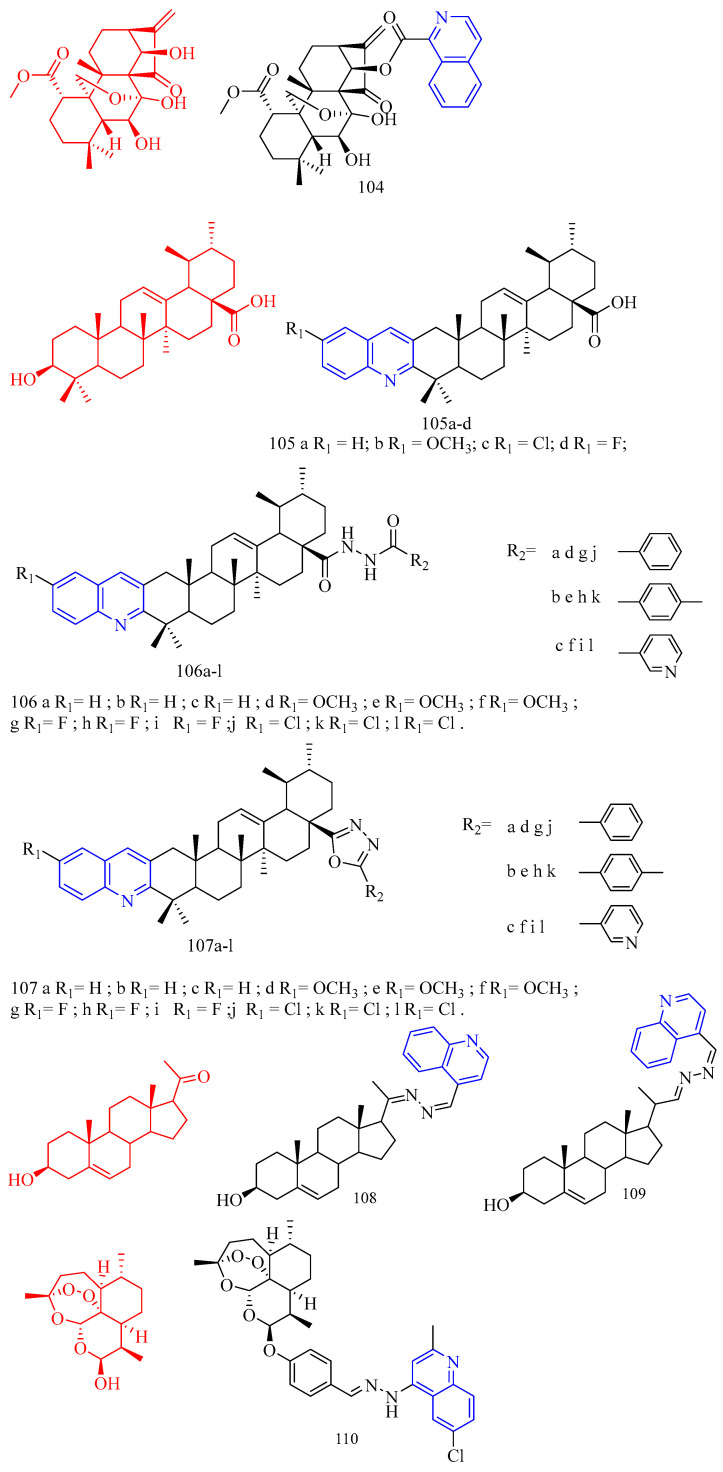
The chemical structures of anticancer compounds **104**–**110**.

Li and colleagues [62] synthesized a lasiokaurin quinoline derivative **104** (Figure 19) (Table 10) and evaluated the antiproliferative activity against human leukemia K562 cells, human gastric cancer MGC-803 cells, human esophageal cancer CaEs-17 cells, and human hepatocellular carcinoma Bel-7402 cells. The results showed that compound **104** inhibited Bel-7402 more strongly than the positive control drug paclitaxel (IC_50_ values for compound **104** were 1.89, 1.03, 1.74, and 0.96 μM, respectively).

Ursolic acid (CF: C_30_H_48_O_3_, MW: 456.700, (3*β*)-3-Hydroxyurs-12-en-28-oic acid) is a natural triterpenoid carboxylic acid compound present in the Labiatae plant *Prunella vulgaris* L. Gu and colleagues [82] synthesized a series of novel ursolic acid quinoline derivatives **105**–**107** (Figure 19) (Table 10) and evaluated their in vitro cytotoxicity against three human cancer cell lines (MDA-MB-231, Hela, and SMMC-7721). From the results, compounds **105a**–**d** exhibited significant antitumor activity against three cancer cell lines. Compounds **105a**–**d**, **106i**, and **111c** showed prominent cytotoxic activities against at least one cancer cell line (IC_50_ < 10 μM). Among them, compound **105b** exhibited the most potent cytotoxic activity against MDA-MB-231, HeLa, and SMMC-7721 cells with IC_50_ values of 0.61 ± 0.07, 0.36 ± 0.05, and 12.49 ± 0.08 μM, respectively, stronger than those of positive control. Compound **105a** also showed anticancer activity against the three cancer cells slightly weaker than compound **105b**. However, compound **105a** had a stronger anticancer activity than that of all other compounds. Compounds **105a**–**d**, **106i**, and **107c** did not show considerable cytotoxicity against normal hepatocyte cells QSG-7701 with IC_50_ > 40 μM. In addition, compounds **106a**, **106c**, **106d**, **106f**, **106g**, **106l**, **107a**, **107d**, **107f**, **107i**, and **107l** showed moderate inhibition to three cancer cell lines. Compounds **106b**, **106e**, **106h**, **106k**, **107b**, **107e**, and **107k** showed weak inhibitory activities against HeLa cells and were not cytotoxic to MDA-MB-231 and SMMC-7721 cells (IC_50_ > 40 μM), while compound **111h** was inactive to all tested cancer cells. Especially, compound **105b** was found to be the most potent derivative with IC_50_ values of 0.61, 0.36, and 12.49 μM against MDA-MB-231, HeLa, and SMMC-7721 cells, respectively, stronger than positive control etoposide.

Gan and colleagues [83] synthesized steroidal quinoline derivatives **108**–**109** (Figure 19) (Table 10) and evaluated their in vitro effects on human HeLa, HT-29, Bel 7404, and antiproliferative activity in SGC 7901 cells. The anticancer activities of compound **108** and cisplatin were comparable (IC_50_ values of 11.2, 21.3, 28.9, and 10.3 μM/L), while the anticancer activity of compound **109** was inferior to that of cisplatin.

Yao and colleagues [84] synthesized the dihydroartemisinin quinoline hydrazone derivative **110** (Figure 19) (Table 10). Using 5-fluorouracil or paclitaxel as positive controls, the results showed that compound **110** showed more pronounced antitumor activity against MCF-7 cells than that of the positive group. In addition, **110** showed low cytotoxicity against normal human cells.

**Figure 20:**
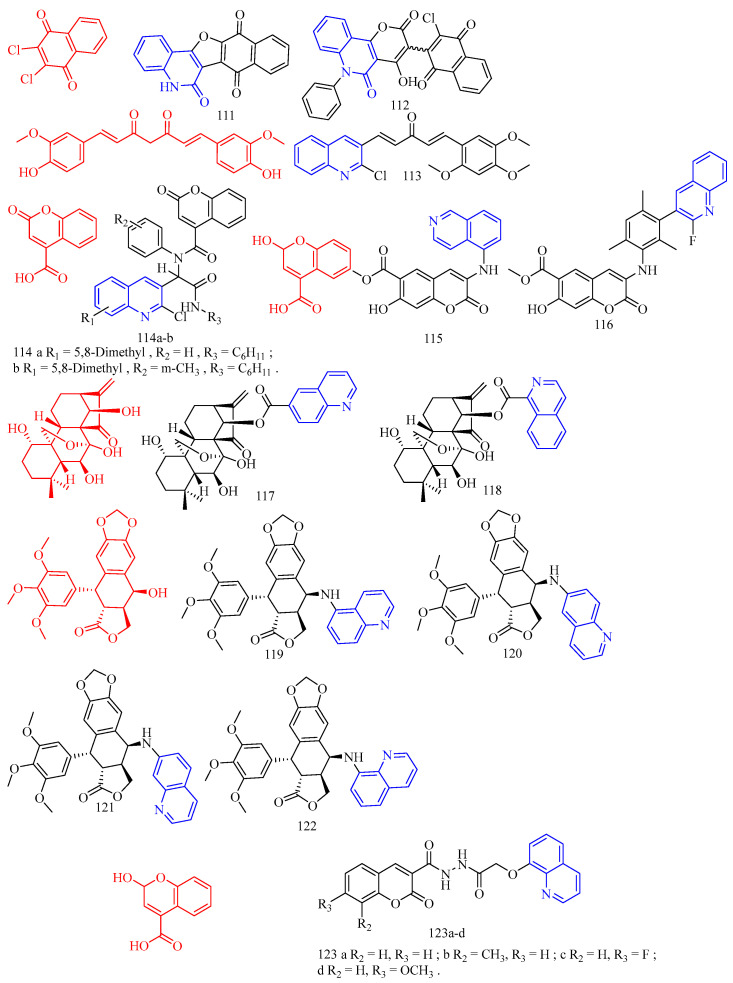
The chemical structures of anticancer compounds **111**–**123**.

Aly and colleagues [85] synthesized a new set of fused naphtho[3,2-c]quinoline-6,7,12-trione and naphtho[3,2-c]quinoline-6,7,8,13-tetrone compounds **111** and **112** (Figure 20) (Table 10) for in vitro anticancer screening. The results showed that compounds **111** and **112** had good potency against ERK, which makes it important to investigate the possible application of these inhibitors in RAF-mutant melanoma (IC_50_ of compounds **111** and **112** were 0.6 and 0.16 μM).

Sri and colleagues [86] synthesized a curcumin-receptor 2-chloro/phenoxy quinoline derivative **113** (Figure 20) (Table 10) and tested their antitumor activity against several cancer cell lines, such as HeLa, HGC-27, NCI-H460, DU-145, PC-3, and 4T1. The IC_50_ ranged from 1.81 to 12.4 μM. The IC_50_ of compound **113** against PC-3, DU-145, NCI-H460, and 4 T1 were 3.12, 3.99, 3.96, and 1.81 μM, respectively.

Taheri and colleagues [87] synthesized coumarin–quinoline derivatives **114a**–**b** (Figure 20) (Table 10) and determined their cytotoxic effects on A2780 human cancer cells using doxorubicin as a positive control. The results showed that the cytotoxicity of compounds **114a**–**b** was significantly higher than that of the other derivatives, with IC_50_ values of 25 and 62 μg/mL, respectively. Further examination revealed that compound **114a** increased ROS levels, decreased MMPs, and induced apoptosis in A2780 cells via the intrinsic mitochondrial pathway; thus, compound **114a** may be an appropriate agent for treating ovarian cancer.

Lipeeva and colleagues [88] synthesized amino coumarin–quinoline derivatives **115**–**116** (Figure 20) (Table 10) and evaluated their antiproliferative effects on leukemia CEM-13, MT-4, U-937, and melanoma MEL-8 cancer cells. Although compound **115** was more cytotoxic to cancer cells than the positive parent compound, they were lower than the positive control drug doxorubicin (IC_50_ values arrange 30.5–47.3 μM). In contrast, the cytotoxicity of compound **116** on MCF-7 cells was comparable to that of the positive control drug doxorubicin. The GI_50_ value of compound **116** was 10.5 μM.

Oridonin (CF: C_20_H_28_O_6_, MW: 364.43, is a biologically active kaurine-type tetracyclic diterpene isolated from the genus plants *Rabdosia* in the family Lamiaceae (Iabtea). Shen and colleagues [89] synthesized oridonin derivatives **117**–**118** (Figure 20) (Table 10) and evaluated their antitumor activity in vitro against three human cancer cell lines, HCT116, BEL7402, and MCF7. Compared with the lead compound and the positive control drug 5-fluorouracil (5-Fu), compounds **117** and **118** exhibited potent antiproliferative efficacy against HCT116, MCF-7, and BEL7402 cancer cell lines. IC_50_ values of 2.51, 0.41, and 2.54 μM, and IC_50_ values for compound **118** were 2.07, 0.89, and 2.30 μM, respectively.

Zhao and colleagues [90] synthesized podophyllotoxin quinoline derivatives **119**–**122** (Figure 20) (Table 10) and evaluated them for antitumor activity assays against the following four human tumor cell lines: hepatocellular carcinoma cells HepG2, cervical cancer cells HeLa, lung cancer cells A549, and breast cancer cells MCF7. Clinical microtubule polymerization inhibitor nocodazole (Ncz), podophyllotoxin clinical drug etoposide (VP-16), PTOX, and DMEP were used as positive controls. The results showed that most of the anticancer activities of compounds **119**–**122** were inferior to the positive control. IC_50_ values arranged 0.8–39.2 μM.

Prashanth and colleagues [91] synthesized coumarin quinoline derivative **123a**–**d** (Figure 20) (Table 10) and evaluated its cytotoxicity in vitro against ascites EAC and Dalton’s lymphoma ascites DLA cells. The results showed that compounds **123a**–**d** had low antitumor activity.

**Figure 21:**
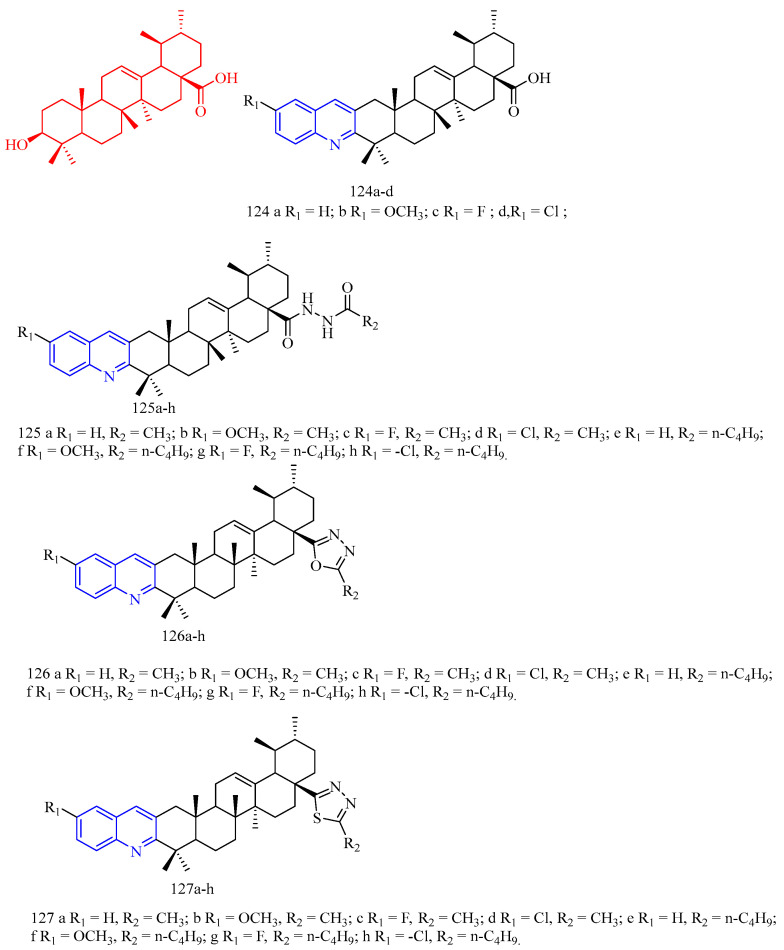
The chemical structures of anticancer compounds **124**–**127**.

Jin and colleagues [92] synthesized ursolic acid quinoline derivatives **124**–**127** (Figure 21) (Table 10) and evaluated their in vitro antiproliferative activity against three cancer cell lines, MDA-MB-231, HeLa, and SMMC 7721; etoposide was used as a positive control. Regarding the different derivatives, compounds **124a**–**d** with carboxyl groups exhibited potent cytotoxic activity against MDA-MB-231 and HeLa cells at low levels of 1 μM and moderate activity against SMMC-7721 cells. Among the acyl hydrazide derivatives **125a**–**h**, compounds **125a**–**d** also exhibited significant cytotoxic activity comparable to that of compounds **124a**–**d**. Compounds **125e**–**h** showed almost no activity against all three cancer cell lines (IC_50_ > 50 μM). Regarding the oxadiazole derivatives **125a**–**h** and thiadiazole derivatives **126a**–**h**, compounds **125a** and **125d** exhibited potent cytotoxicity (IC_50_ < 10 μM) against MDA-MB-231 and HeLa cells, respectively. Compounds **126b**–**c**, **127a**–**b**, and **127d** showed moderate activity against MDA-MB-231 and HeLa cells, while compounds **126e**–**h**, **127c**, and **127e**–**h** showed only slight or no cytotoxicity against the three cancer cell lines.

**Figure 22:**
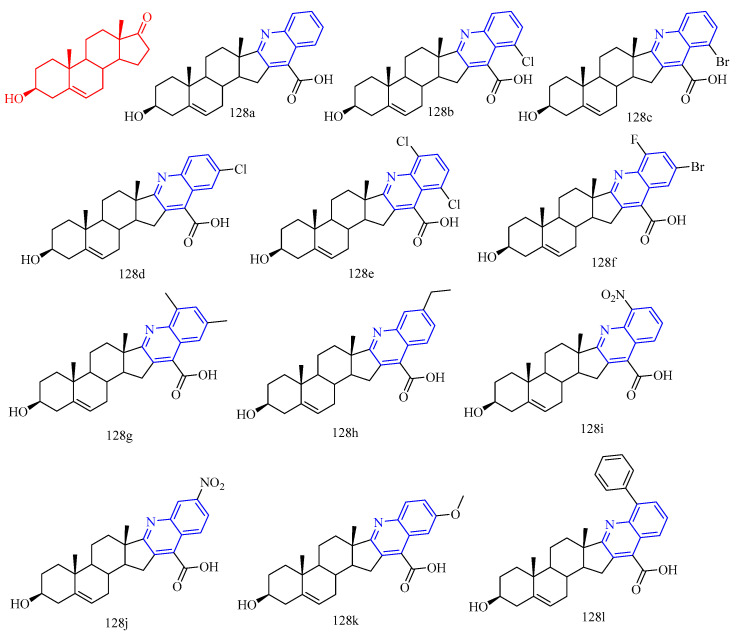
The chemical structures of anticancer compounds **128**.

Yang and colleagues [93] synthesized steroidal quinoline derivatives **128a**–**l** (Figure 22) (Table 10) and evaluated their in vitro antiproliferative activity against three human lung cancer cells, A549, A431, and H1975. Compounds **128a** mildly inhibited the growth of A549, A431, and H1975 with IC_50_ values of 15.21, 17.45, and 20.76 μM, respectively. Compounds 162b–d with halogen atoms were found to have comparable activity to **128a**, while compounds **128e**–**f** showed better antiproliferative activity against the tested lung cancer cells. In particular, compound **128f** showed the highest potency against A549, A431, and H1975 with IC_50_ values of 5.34, 6.21, and 7.25 μM, respectively. Compounds **128g**–**h** with alkyl groups showed reduced but moderate antiproliferative activity compared to **128f**; moreover, nitro-containing compounds **128i**–**j** also showed moderate inhibitory activity against the tested cancer cells. Apparently, compounds **128k**–**l** containing methoxy and phenyl, respectively, showed weaker growth inhibition against the tested cancer cells.

**Figure 23:**
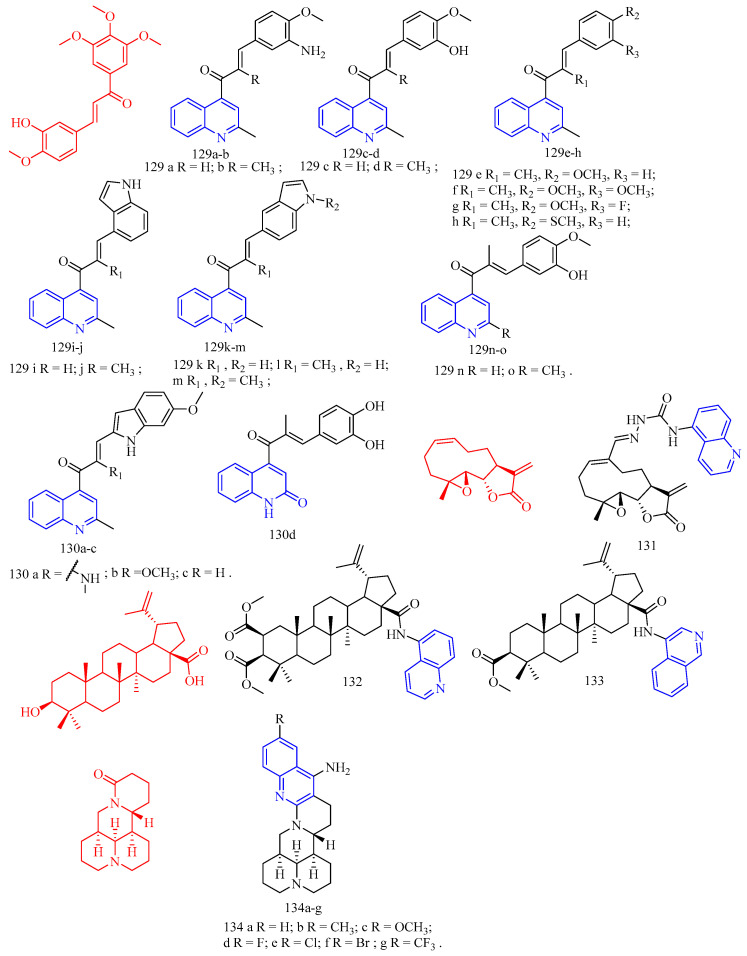
The chemical structures of anticancer compounds **129**–**134**.

Li and colleagues [94] synthesized chalcone–quinoline derivatives **129**–**130** (Figure 23) (Table 10) and evaluated their antiproliferative effects in vitro, and compared them with the reference compound CA-4. Human chronic myeloid leukemia cell K562 was used for the first time. The results showed that all the newly synthesized compounds exhibited good antiproliferative activities in the nanomolar range, except for compounds **129j**–**k** and **129n**–**o**, which have an indole moiety as the B ring. Among them, compounds **129b** and **129d** with 3-amino-4-methoxyphenyl or 3-hydroxy-4-methoxyphenyl moieties showed the most potent activities with IC_50_ values of 0.011 and 0.009 μM, respectively, which were comparable to CA-4 (IC_50_ = 0.011 μM) and approximately 6-fold stronger than the parent compound (IC_50_ = 0.060 μM). The methyl substituent at the α-position of the unsaturated carbonyl increased activity (**129a** vs. **129b**, **129c** vs. **129d**, and **129k** vs. **129l**), and in compounds **129i**–**o**, except for compounds **129l** and **129m**, the activity of most compounds in this series (IC_50_ > 1 μM) is lower than its phenyl counterpart whose unsaturated double bond is substituted at the C-5 position of the indole moiety. In addition, the methyl substituent at the N-1 position of indole (**129m**) exhibited approximately 5-fold increased activity compared to the unsubstituted counterpart **129l**. All compounds **130a**–**d** showed good activity except **130d**, which has a lactam instead of a quinoline ring. The steric hindrance of the C-2 group of the quinoline moiety appears to have a key effect on the activity, as compounds with smaller substitutions, such as CH_3_ (**129d** IC_50_ = 0.009 μM), NHCH_3_ (**130a** IC_50_ = 0.018 μM), OCH_3_ (**130b** IC_50_ = 0.030 μM), and H (**130c** IC_50_ = 0.015 μM), were more active than other compounds with larger groups. Interestingly, the CH_3_-substituted compound **129d** exhibits slightly stronger activity than the corresponding unsubstituted counterpart **130c**, despite the greater steric hindrance of methyl groups than hydrogen. The biological functions of more cancer cell lines were further evaluated. Four additional cancer cell lines, including human hepatocellular carcinoma (HepG2), nasopharyngeal epidermoid carcinoma (KB), human colon carcinoma cells (HCT-8), and human breast cancer cells (MDA-MB-231), were selected for further evaluation. K562 cells were the most sensitive of the five cancer cell lines tested, and the most active compound, **129d**, exhibited comparable activity to the reference compound CA-4, with IC_50_ values ranging from 0.009 to 0.016 μM. Notably, the activity of **129d** increased approximately 6-fold compared to the parent compound; therefore, **129d** was selected for further biological studies. In addition, the selectivity ratio of **129d** to normal human liver L-O2 cells was 65.8 times higher than that of CA-4, indicating that the toxicity of **129d** may be lower than that of CA-4.

Parthenolide (CF: C_15_H_20_O_3_, MW: 248.32, (1aR,4E,7a*S*,10a*S*,10b*R*)-2,3,6,7,7a,8,10a,10b-Octahydro-1a,5-dimethyl-8-methyleneoxireno[9,10]cyclodeca[1,2-b]furan-9(1a*H*)-one) is a natural sesquiterpene lactone product isolated from medicinal plants, such as Salvia miltiorrhiza and Salvia miltiorrhiza. Jia and colleagues [95] synthesized a parthenolide quinoline derivative **131** (Figure 23) (Table 10) and determined its cytotoxic activity in HCT116, U87-MG, HepG2, BGC823, and PC9. Both paclitaxel (paclitaxel) and PTL (1a) were used as positive controls. The IC_50_ values for compound **131** were 3.31, 1.47, 3.66, 1.77, and 3.12 μM.

Betulinic acid (CF: C_30_H_48_O_3_, MW: 456.70, (3*β*)-3-Hydroxylup-20(29)-en-28-oic acid) is a natural lupin-type pentacyclic triterpenoid present in the leaves of *Cyperus rotundus*, the bark of birch trees and date palm kernels extracted from them. Platanic acid (CF: C_29_H_46_O_4_, MW: 458.67, (3*β*)-3-Hydroxy-20-oxo-30-norlupan-28-oic acid) is a pentacyclic triterpenoid isolated from the leaves of *Syzygium claviflorum*. Hoenke and colleagues [96] prepared betulinic acid quinoline derivatives **132** (Figure 23) (Table 10) and platanic acid quinoline derivatives **133** and screened them for cytotoxicity. Compound **132** was the most cytotoxic in this study and also had the highest tumor or non-tumor cell selectivity, especially for A375 melanoma (S = 91.2), A2780 ovarian cancer (S = 61.6), and hypopharyngeal carcinoma FaDu (S = 59.0) cells. Compound **132** was slightly less selective than 167 (selectivity S was 41.4, 4.0, 19.2, 37.0, and 34.4, respectively). A375 melanoma cells were used in order to facilitate the understanding of their cytotoxicity pattern. The results showed that compound 166 also increased the number of apoptotic cells; however, more cells were in advanced stages. Similar behavior was observed when cells were treated with **133** (8.6% apoptotic and 9.6% late-stage apoptotic cells). In conclusion, compound **133**, a 4-isoquinolinamide of 3-O-acetyl-leucovorin acid, was the most cytotoxic compound with EC_50_ values as low as EC_50_ = 1.48 μM (A375 melanoma cells) and was also cytotoxic against non-malignant fibroblasts with an NIH 3T3 selectivity index > 91.2.

Xu and colleagues [97] designed and synthesized matrine quinoline derivatives **134a**–**g** (Figure 23) (Table 10), using cisplatin as positive drug control, and evaluated compounds for anticancer activity against HepG2, HeLa, and MDA-MB-231 cell lines. Compound **134a**–**g** showed good activity against HepG2, HeLa and MDA MB-231 cell lines with IC_50_ below 25 μM.

**Figure 24:**
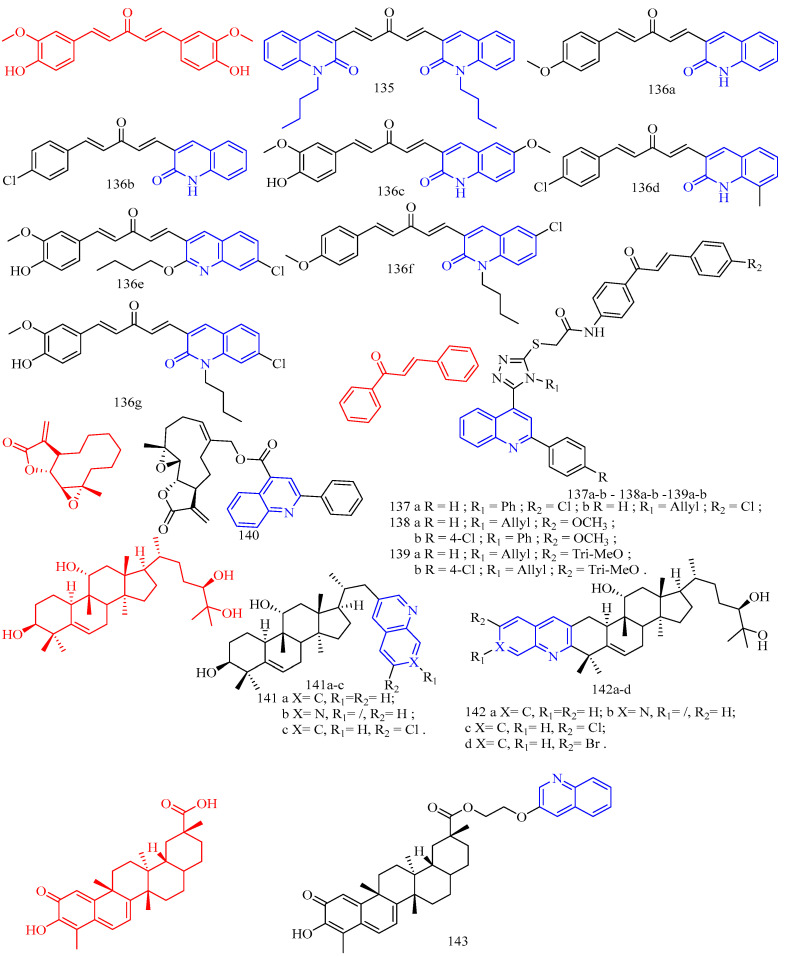
The chemical structures of anticancer compounds **135**–**143**.

Insuasty and colleagues [98] synthesized a series of quinoline-based symmetrical and asymmetrical bis-acetal compounds **135**–**136** (Figure 24) (Table 10). These compounds were evaluated for their in vitro cytotoxic activity against different human cancer cell lines. Compounds **135**, **136a**, **136d**, **136f**, and **136g** showed the highest activity, while compounds **136b**, **136c**, and **136e** showed moderate activity. Symmetrical N-butyl quinoline chalcone **135** and asymmetrical bis-chalcone **136g** exhibited the highest cytotoxicity with overall GI_50_ values ranging from 0.16 to 5.45 μM, with excessive activity of HCT-116 (GI_50_ = 0.16 μM) and HT29 (GI_50_ = 0.42 μM) (colon cancer). Notably, several GI_50_ values of these compounds were superior to the reference drugs doxorubicin and 5-FU.

Mohassab and colleagues [99] developed novel quinoline/chalcone derivatives **137**–**139** (Figure 24) (Table 10) and tested them in vitro against a panel of cancer cell lines and EGFR and BRAFV600E anticancer targets. The most active compounds **137a**–**b** and **138a**–**b** effectively inhibited cancer cell growth. After compound **137b**, the difference observed between compounds **139a**–**b** was almost comparable and extreme in terms of anticancer activity with GI_50_ cell lines of 3.625 μM and 4.550 μM, respectively. In contrast, compound **137b** showed the highest activity among all the new compounds with a GI_50_ of 3.325 μM to inhibit the growth of cancer cells.

Zeng and colleagues [100] synthesized parthenolide quinoline heterodimer **140** (Figure 24) (Table 10) and evaluated the compounds for in vitro antiproliferative activity in five human cancer cell lines, HCT116, U85MG, HepG2, HepG2, BGC823, and PC9. Paclitaxel (PTX) was used as an experimental control. PTL and MCL were used as positive controls for their inhibition of NF-κB and STAT3. The results showed that compound **140** exhibited higher cytotoxicity than PTL and MMB for all five cell lines. IC_50_ values range from 2.11 to 5.23 μM/L, respectively.

Mogrol (CF: C_30_H_52_O_4_, MW: 476.73, (3*β*,9*β*,10*α*,11*α*,24*R*)-9-Methyl-19-norlanost-5-ene-3,11,24,25-tetrol) is a polysaccharide of Luo Han Guo saponin, which is the main active component of Luo Han Guo. Song and his colleagues [101] synthesized mogrol quinoline derivatives **141**–**142** (Figure 24) (Table 10) and evaluated the in vitro cytotoxicity of the compounds against human lung cancer cell lines A549 and NCI-H460. A quinoline scaffold was introduced to generate **141a**–**c** and **142a**–**d**, and the cyclic A-fusion derivative **142a**–**d** exhibited higher activity than **141a**–**c** against the tested cell lines. Compounds **141a**–**b** and **142a**–**d** showed stronger inhibitory activity than mogrol against A549 with IC_50_ values ranging from 12.94 to 19.24 μM. The R_1_ and R_2_ substituents on the quinoline moiety significantly affected the activity, and the presence of the halogen atom resulted in a decrease in cytotoxic activity. All quinoline derivatives except compound **141c** were more active than mogrol against NCI-H460, while compound **142a** showed the highest activity with an IC_50_ value of 17.13 μM.

Celastrol (CF: C_29_H_38_O_4_, MW: 450.61, (9*β*,13*α*,14*β*,20*α*)-3-Hydroxy-9,13-dimethyl-2-oxo-24,25,26-trinoroleana-1(10),3,5,7-tetraen-29-oic acid) is a pentacyclic triterpene from Tripterygia Wilfordil Hook. f., a pentacyclic triterpene. Shang and colleagues [102] synthesized celastrol quinoline derivative **143** (Figure 24) (Table 10) and evaluated the toxicity of this compound on Hep3B cells, with celastrol being used as a positive control. Compound **143** with a 3-quinoxyl ethyl substituent had the strongest HIF-1a inhibitory activity in this study, with an IC_50_ value of only 0.05 μM, which was 5-fold higher than the activity of celastrol (IC_50_ = 0.25 μM). In addition, Western blot results showed that compound **143** could inhibit the expression of HIF-1a protein. Further experiments showed that **143** significantly inhibited the formation of Hep3B cell colonies, hindered cell migration, and induced apoptosis to some extent. Compound **143** (10 mg/kg) had good in vivo antitumor activity in a mouse tumor xenograft model with an inhibition rate of 74.03%, which was superior to the reference compound 5-FU inhibition rate (59.58%).

**Figure 25:**
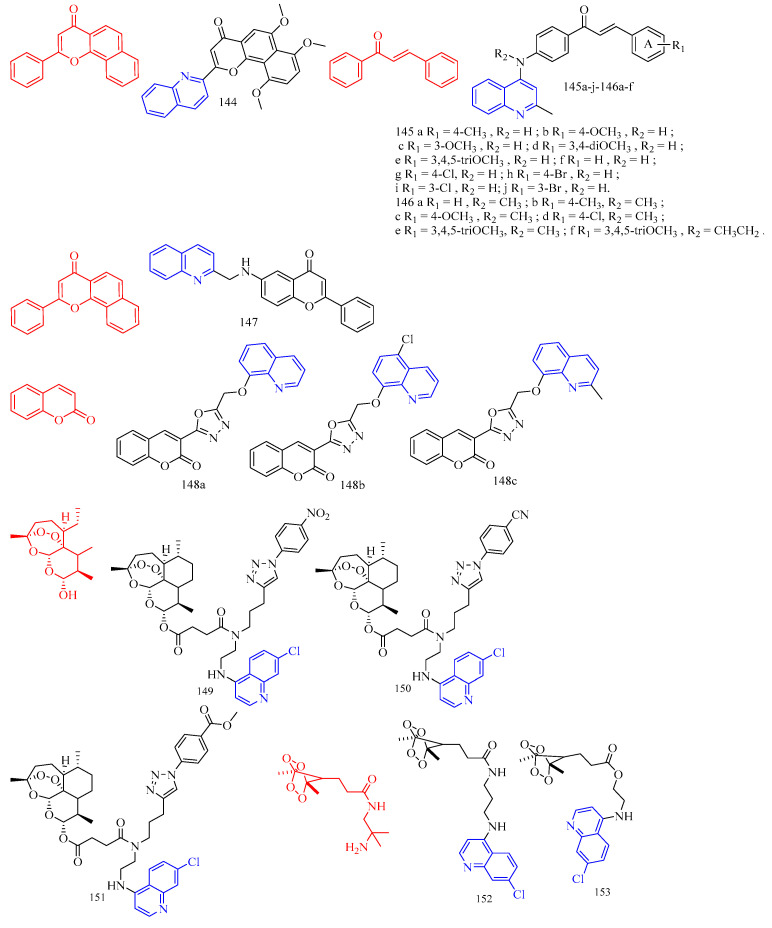
The chemical structures of anticancer compounds **144**–**153**.

Dong and colleagues [103] synthesized 6,7,10-trimethoxy-α-naphthoflavone-quinoline derivative **144** (Figure 25) (Table 10) and screened it against the CYP1 enzyme to assess whether larger substituents could enhance the inhibitory activity against CYP1B1. Compound **144** selectively inhibited CYP1B1. IC_50_ values of 117.6, 1.0 and >1000 μM for CYP1A1, CYP1B1, and CYP1A2, respectively.

Guan and colleagues [104] synthesized chalcone quinoline derivatives **145**–**146** (Figure 25) (Table 10) and evaluated the in vitro antiproliferative activity of the compounds against MGC-803, HCT-116, and MCF-7. The chemotherapy medication 5-Fluorouracil (5-Fu) was used as a positive control. Most quinoline–chalcone derivatives showed strong antiproliferative activity against MGC-803, HCT-116, and MCF-7 cells with IC_50_ values < 20 μM. Among them, compound **145e** showed the most remarkable inhibitory effect on MGC803, HCT-116, and MCF-7 cells with IC_50_ values of 1.3, 5.34, and 5.21 μM, respectively, which was much lower than that of 5-Fu (IC_50_ values = 6.22 μM, 0.4 μM, and 11.1 μM, respectively). Thus, the antiproliferative activity of compound **145e** on MGC803 cells, structure–activity relationships suggests that the type and position of the substituent (R1) on the chalcone moiety (A ring) have an important effect on its antiproliferative activity. Compared with **145f**, the activity of compounds **145a**–**e** with the electron-donating group of A ring is higher than that of unsubstituted A ring. However, compounds **145g** and **145i** and the compound with an electron-withdrawing group of A ring inhibit proliferation more actively than the compound **145f**. In addition, the position of the substituent (R) is also important. When the substituent (R) is located at the 3 position of the chalcone group (A ring), the inhibitory activity of the compound is lower than when the substituent (R_1_) is located at the 3 position of the A ring (compound **145b** vs. **145c**, **145g** vs. **145i**, and **145h** vs. **145i**). However, compound **145e** with the 3,4,5-triOCH substituent of the chalcone group (A ring) showed better activity. The relationship between the electron-donating group and an electron-withdrawing group of the chalcone group (A ring) and the inhibition of MGC-803 cells is 3,4,5-triOCH_3_ > 3,4-diOCH_3_ > 4-CH_3_ > 4-bromo > 4-OCH_3_ > 3-OCH_3_ > 3-Br > H > 4-Cl > 3-Cl. Next, the impact of R_2_ is further explored. The results showed that the inhibitory activity of compounds **146a**–**f** decreased when the H group was substituted with CH_3_ or CH_3_CH_2_ substituents (compounds **146a** vs. **145f**, **146b** vs. **145a**, **146c** vs. **145d**, **146d** vs. **145g**, **146e** vs. **145e**, and **146f** vs. **145e**), indicating that the R_2_ substituent does not increase the inhibitory potency. The in vitro antiproliferative activities of novel target compounds **145a**–**i** and **146a**–**f** were evaluated against the human cell lines MGC-803 (gastric cancer), HCT-116 (colon cancer), and MCF-7 (breast cancer), with 5-fluorouracil (5-Fu) as a positive control. The results showed that most quinoline–chalcone derivatives have strong antiproliferative activities against the MGC-803HCT-116 and MCF-7 cells, with IC_50_ values < 20uM. Among them, compound **145e** has the greatest inhibitory effect on MGC803, HCT-116, and MCF-7 cells, with IC_50_ values of 1.35.34 and 5.21 μM, respectively, which is much lower than that of 5-Fu (IC_50_ value = 6.22 μm) of 0.4 μM, 5.21 μM, and 11.1 μM, respectively, indicating that compound **145e** has an inhibitory effect on the activity of three tumor cells. In addition, the MGC-803 cells are more sensitive to most compounds than the HCT-116 and MCF-7 cells. Therefore, according to the structure–activity relationship of the antiproliferative activity of MGC803 cells, the type and position of the substituent (R_1_) on the chalcone group (A ring) were related to its antiproliferative activity.

Thorat and colleagues [105] synthesized the 6-amino flavonoid quinoline derivative **147** (Figure 25) (Table 10) and evaluated the in vitro antiproliferative efficacy of compound **147** against MCF-7 and human A-549. Doxorubicin was used as a positive control. Compound **147** showed satisfactory anticancer activity against MCF-7, with 44.76% inhibition against this cancer cell line. Compound **147** also showed acceptable antiproliferative activity against A-549, exhibiting the cell at 44.26% under a concentration of 10 μM.

Jyothi and colleagues [106] synthesized coumarin quinoline derivatives **148a**–**c** (Figure 25) (Table 10) and evaluated their activities against the human-derived cancer cells ACHN, A375, SIHA, Skov3, EAC, and NIH3T3. However, compounds **148a**–**c** did not demonstrate any anticancer activity.

Herrmann and colleagues [107] synthesized artemisinin-quinoline derivatives **149**–**153** (Figure 25) (Table 10) and analyzed their inhibitory activity in vitro against leukemia cell lines CCRF-CEM, RPMI-8226, K562, HL-60, and MOLT 4. The data showed that artemisinins **149**–**151** and synthetic peroxo-quinolines **152** and **153** were the most active compounds against the K562 leukemia cell line in vitro, with IC_50_ values of 37.3 and 83.0 μM, respectively.

**Table 10:** Quinoline derivatives with anticancer activity.

Compd.	Activity	Target	Origin	Ref
**43**	Anti- TK10 TGI = 18.5 μM Anti- UACC62 TGI = 17.43 μM Anti- MCF7 TGI = 2.92 μM	-	synthetic	[39]
**44**	-	-	synthetic	[39]
**71a**	Determine growth inhibitory activity = 85%	-	synthetic	[59]
**71b**	Determine growth inhibitory activity = 77%	-	synthetic	[59]
**77**	Anti-melanoma GI_50_ = 0.134 μM	-	synthetic	[63]
**78a**	Anti- Ae549 IC_50_ = 15.4 μM Anti- A375 IC_50_ = 14.5 μM Anti- MCF-7 IC_50_ = 13.8 μM Anti- HT-29 IC_50_ = 12.3 μM Anti- ACHN IC_50_ = 10.8 μM	DNA topoisomerase-IIa	synthetic	[64]
**78b**	Anti- Ae549 IC_50_ = 13.4 μM Anti- A375 IC_50_ = 7.7 μM Anti- MCF-7 IC_50_ = 11.2 μM Anti- HT-29 IC_50_ = 7.75 μM Anti- ACHN IC_50_ = 15.7 μM	DNA topoisomerase-IIa	synthetic	[64]
**79a**	Anti- Ae549 IC_50_ = 10.6 μM Anti- A375 IC_50_ = 10.3 μM Anti- MCF-7 IC_50_ = 8.6 μM Anti- HT-29 IC_50_ = 11.8 μM Anti- ACHN IC_50_ = 10.7 μM	DNA topoisomerase-IIa	synthetic	[64]
**79b**	Anti- Ae549 IC_50_ = 7.7 μM Anti- A375 IC_50_ = 6.8 μM Anti- MCF-7 IC_50_ = 2.2 μM Anti- HT-29 IC_50_ = 8.9 μM Anti- ACHN IC_50_ = 9.46 μM	DNA topoisomerase-IIa	synthetic	[64]
**80**	Anti- angiogenesis inhibition rate ranged between 50 and 60%	-	synthetic	[65]
**81**	Anti-A549 GI_50_ = 2.51 μM	Erk1/2 signaling pathway	synthetic	[66]
**82**	Anti-HepG2 IC_50_ = 16.03 μM Anti-HeLa IC_50_ = 0.60 μM Anti-A549 IC_50_ = 10.05 μM Anti-BGC-823 IC_50_ = 17.41 μM Anti-HL-7702 IC_50_ = 41.77 μM	Caspase-3	synthetic	[67]
**83**	Anti-HepG2 IC_50_ = 9.18 μM Anti-HeLa IC_50_ = 20.53 μM Anti-A549 IC_50_ = 19.20 μM Anti-BGC-823 IC_50_ = 28.81 μM Anti-HL-7702 IC_50_ = 20.09 μM	Caspase-3	synthetic	[67]
**84**	Anti-HL-60 IC_50_ = 0.1 μM Anti-MCF-7 IC_50_ = 0.1 μM Anti-T-47D IC_50_ = 0.1 μM Anti-LNCaP IC_50_ = 2.0 μM Anti-WEHI-3 IC_50_ = 1.1 μM	-	synthetic	[68]
**85**	Anti-SGC 7901 IC_50_ = 1 μM	Topoisomerase II	synthetic	[69]
**86**	Anti-MCF-7 IC_50_ = 181.478 μM Anti-MCF-7/ADR IC_50_ = 96.523 μM Anti-SW1990 IC_50_ = 111.837 μM Anti-SMMC-7721 IC_50_ = 75.546 μM	-	synthetic	[70]
**87**	Anti-HepG2 IC_50_ = 8.40 μM Anti-A431 IC_50_ = 11.56 μM Anti-A549 IC_50_ = 4.33 μM Anti-MCF 7 IC_50_ = 5.99 μM Anti-HCT 116 IC_50_ = 3.48 μM	-	synthetic	[71]
**88**	Anti-MDA-MB 468 IC_50_ = 0.12 μM	Microtubule	synthetic	[72]
**89**	Anti-SKOV3 IC_50_ = 12.8 μM	oxygen species (ROS)	synthetic	[73]
**90**	Anti-Bel-7404 IC_50_ = 28.7 µmol/L Anti-SGC-7901 IC_50_ = 17.9 µmol/L	-	synthetic	[74]
**91**	Anti-HT-29 IC_50_ = 14.1 μmol/L Anti-HeLa IC_50_ = 9.1 μmol/L Anti-SGC-7901 IC_50_ = 8.2 μmol/L	-	synthetic	[75]
**92a**	Anti-Hela 30 μM IC_50_ = 59.5 μM	Caspase-3	synthetic	[76]
**92b**	Anti-T47D 30 μM IC_50_ = 48.1 μM	Caspase-3	synthetic	[76]
**92c**	Anti-T47D 30 μM IC_50_ = 63.0 μM	Caspase-3	synthetic	[76]
**93a**	Anti- MCF7 30 μM IC_50_ = 31.5 μM	Caspase-3	synthetic	[76]
**93b**	Anti-SiHa 10 μM IC_50_ = 24.7 μM	Caspase-3	synthetic	[76]
**93c**	Anti-MCF7 30 μM IC_50_ = 35.6 μM	Caspase-3	synthetic	[76]
**93d**	Anti- SiHa 30 μM IC_50_ = 25.6 μM	Caspase-3	synthetic	[76]
**93e**	Anti-Hela 30 μM IC_50_ = 96.6 μM	Caspase-3	synthetic	[76]
**93f**	Anti- Hela 30 μM IC_50_ = 89.4 μM	Caspase-3	synthetic	[76]
**94a**	Anti-MCF7 30 μM IC_50_ = 38.6 μM	Caspase-3	synthetic	[76]
**94b**	Anti-C33A 30 μM IC_50_ = 61.0 μM	Caspase-3	synthetic	[76]
**94c**	Anti-MDA-MB-231 30 μM IC_50_ = 71.2 μM	Caspase-3	synthetic	[76]
**94d**	-	Caspase-3	synthetic	[76]
**94e**	Anti-MDA-MB-361 30 μM IC_50_ = 66.0 μM	Caspase-3	synthetic	[76]
**94f**	Anti-MDA-MB-361 30 μM IC_50_ = 68.2 μM	Caspase-3	synthetic	[76]
**94g**	Anti-MCF7 30 μM IC_50_ = 62.9 μM	Caspase-3	synthetic	[76]
**94h**	-	Caspase-3	synthetic	[76]
**94i**	-	Caspase-3	synthetic	[76]
**95a**	Anti-C33A 30 μM IC_50_ = 93.6 μM	Caspase-3	synthetic	[76]
**95b**	Anti- C33A 30 μM IC_50_ = 71.0 μM	Caspase-3	synthetic	[76]
**95c**	Anti-MDA-MB-361 30 μM IC_50_ = 80.4 μM	Caspase-3	synthetic	[76]
**95d**	Anti-C33A 30 μM IC_50_ = 73.1 μM	Caspase-3	synthetic	[76]
**95e**	Anti-C33A 30 μM IC_50_ = 86.4 μM	Caspase-3	synthetic	[76]
**95f**	Anti-C33A 30 μM IC_50_ = 86.9 μM	Caspase-3	synthetic	[76]
**95g**	Anti-C33A 30 μM IC_50_ = 74.7 μM	Caspase-3	synthetic	[76]
**95h**	Anti-MDA-MB-361 30 μM IC_50_ = 75.2 μM	Caspase-3	synthetic	[76]
**96**	Assembly of purified tubulin IC_50_ = 1.6 μM	Tubulin	synthetic	[77]
**97a**	Anti-NIH 3T3 EC_50_ = 0.9 μM	-	synthetic	[78]
**97b**	Anti-NIH 3T3 EC_50_ = 2.2 μM	-	synthetic	[78]
**97c**	Anti-A2780 EC_50_ = 2.0 μM	-	synthetic	[78]
**97d**	Anti-A2780 EC_50_ = 3.5 μM	-	synthetic	[78]
**97e**	Anti-A2780 EC_50_ = 0.7 μM	-	synthetic	[78]
**97f**	Anti-518A2 EC_50_ = 2.0 μM	-	synthetic	[78]
**97g**	Anti-NIH 3T3 EC_50_ = 0.6 μM	-	synthetic	[78]
**97h**	Anti-NIH 3T3 EC_50_ = 0.9 μM	-	synthetic	[78]
**97i**	Anti- NIH 3T3 EC_50_ = 0.7 μM	-	synthetic	[78]
**97j**	Anti- MCF7 EC_50_ = 1.6 μM	-	synthetic	[78]
**97k**	Anti-A2780 EC_50_ = 5.1 μM	-	synthetic	[78]
**97l**	Anti-A2780 EC_50_ = 6.7 μM	-	synthetic	[78]
**98**	Anti-A2780 EC_50_ = 1.2 μM	-	synthetic	[78]
**99a**	Anti-A2780 IC_50_ = 8.04 μM	Tubulin	synthetic	[79]
**99b**	Anti-MCF-7/MX IC_50_ = 21.48 μM	Tubulin	synthetic	[79]
**99c**	Anti-A2780 IC_50_ = 9.19 μM	Tubulin	synthetic	[79]
**99d**	Anti-A2780 IC_50_ = 7.98 μM	Tubulin	synthetic	[79]
**99e**	Anti- A2780RCIS IC_50_ = 8.15 μM	Tubulin	synthetic	[79]
**100**	Anti-K562/ADR IC_50_ = 0.061 μM Anti-K562 IC_50_ = 0.064 μM	MAPK	synthetic	[80]
**101**	Anti-K562/ADR IC_50_ = 0.177 μM Anti-K562 IC_50_ = 0.064 μM	MAPK	synthetic	[80]
**102**	Anti-K562/ADR IC_50_ = 0.034 μM Anti-K562 IC_50_ = 0.022 μM	MAPK	synthetic	[80]
**103**	Anti-HL60 IC_50_ = 8.09 μM Anti-SGC-7901 IC_50_ = 73.40 μM Anti-MCF-7 IC_50_ = 19.66 μM Anti-HCT116 IC_50_ = 14.79 μM Anti-A549 IC_50_ = 17.61 μM Anti- HaCat IC_50_ = 11.49 μM	-	synthetic	[81]
**104**	Anti-Bel-7402 IC_50_ = 0.96 μM Anti-K562 IC_50_ = 1.89 μM Anti-MGC-803 IC_50_ = 1.03 μM Anti-CaEs-17 IC_50_ = 1.74 μM	-	synthetic	[62]
**105a**	Anti-HeLa IC_50_ = 0.37 μM	-	synthetic	[82]
**105b**	Anti-HeLa IC_50_ = 0.36 μM	-	synthetic	[82]
**105c**	Anti-HeLa IC_50_ = 1.22 μM	-	synthetic	[82]
**105d**	Anti-MDA-MB-231 IC_50_ = 0.90 μM	-	synthetic	[82]
**106a**	Anti-HeLa IC_50_ = 19.03 μM	-	synthetic	[82]
**106b**	Anti-HeLa IC_50_ = 25.78 μM	-	synthetic	[82]
**106c**	Anti-MDA-MB-231 IC_50_ = 13.34 μM	-	synthetic	[82]
**106d**	Anti-MDA-MB-231 IC_50_ = 17.44 μM	-	synthetic	[82]
**106e**	Anti-HeLa IC_50_ = 21.88 μM	-	synthetic	[82]
**106f**	Anti-HeLa IC_50_ = 12.27 μM	-	synthetic	[82]
**106g**	Anti-HeLa IC_50_ = 13.13 μM	-	synthetic	[82]
**106h**	Anti-HeLa IC_50_ = 23.45 μM	-	synthetic	[82]
**106i**	Anti-HeLa IC_50_ = 7.16 μM	-	synthetic	[82]
**106j**	Anti-HeLa IC_50_ = 26.87 μM	-	synthetic	[82]
**106k**	Anti-HeLa IC_50_ = 30.25 μM	-	synthetic	[82]
**106l**	Anti-HeLa IC_50_ = 12.45 μM	-	synthetic	[82]
**107a**	Anti-MDA-MB-231 IC_50_ = 22.37 μM	-	synthetic	[82]
**107b**	Anti-HeLa IC_50_ = 32.13 μM	-	synthetic	[82]
**107c**	Anti-HeLa IC_50_ = 7.11 μM	-	synthetic	[82]
**107d**	Anti-MDA-MB-231 IC_50_ = 19.39 μM	-	synthetic	[82]
**107e**	Anti-HeLa IC_50_ = 28.12 μM	-	synthetic	[82]
**107f**	Anti-HeLa IC_50_ = 12.31 μM	-	synthetic	[82]
**107g**	Anti-MDA-MB-231 IC_50_ = 23.35 μM	-	synthetic	[82]
**107h**	Anti-MDA-MB-231 IC_50_ > 40 μM	-	synthetic	[82]
**107i**	Anti-HeLa IC_50_ = 16.29 μM	-	synthetic	[82]
**107j**	Anti-HeLa IC_50_ = 28.29 μM	-	synthetic	[82]
**107k**	Anti-HeLa IC_50_ = 32.25 μM	-	synthetic	[82]
**107l**	Anti-HeLa IC_50_ = 12.09 μM	-	synthetic	[82]
**108**	Anti-HeLa IC_50_ = 11.2 μM/L Anti-HT-29 IC_50_ = 21.3 μM/L Anti-Bel 7404 IC_50_ = 28.9 μM/L Anti-SGC 7901 IC_50_ = 10.3 μM/L	-	synthetic	[83]
**109**	-	-	synthetic	[83]
**110**	-	Cysteine protease falcipain-2	synthetic	[84]
**111**	Anti-ERK IC_50_ = 0.6 μM	ERK2	synthetic	[85]
**112**	Anti-ERK IC_50_ = 0.16 μM	ERK2	synthetic	[85]
**113**	Anti-PC-3 IC_50_ = 3.12 μM Anti-DU-145 IC_50_ = 3.99 μM Anti-NCI-H460 IC_50_ = 3.96 μM Anti-4 T1 IC_50_ = 1.81 μM	-	synthetic	[86]
**114a**	Anti-A2780 IC_50_ = 25 μg/mL	-	synthetic	[87]
**114b**	Anti-A2780 IC_50_ = 62 μg/mL	-	synthetic	[87]
**115**	Anti-MCF-7 GI_50_ = 38.3 Μm	-	synthetic	[88]
**116**	Anti-MCF-7 GI_50_ = 10.5 μM	-	synthetic	[88]
**117**	Anti-MCF-7 IC_50_ = 0.41 μM	p53-MDM2	synthetic	[89]
**118**	Anti-MCF-7 IC_50_ = 0.89 μM	p53-MDM2	synthetic	[89]
**119**	Anti-MRC-5 IC_50_ = 70.8 μM	Tubulin	synthetic	[90]
**120**	Anti-MCF-7 IC_50_ = 0.6 μM	Tubulin	synthetic	[90]
**121**	Anti-A549 IC_50_ = 2.3 μM	Tubulin	synthetic	[90]
**122**	Anti-MCF-7 IC_50_ = 1.0 μM	Tubulin	synthetic	[90]
**123a**	low antitumor activity	Caspase-3	synthetic	[91]
**123b**	low antitumor activity	Caspase-3	synthetic	[91]
**123c**	low antitumor activity	Caspase-3	synthetic	[91]
**123d**	low antitumor activity	Caspase-3	synthetic	[91]
**124a**	Anti-HeLa IC_50_ = 0.37 μM	Ras/Raf/MEK/ERK	synthetic	[92]
**124b**	Anti-HeLa IC_50_ = 0.36 μM	Ras/Raf/MEK/ERK	synthetic	[92]
**124c**	Anti-MDA-MB-231 IC_50_ = 0.90 μM	Ras/Raf/MEK/ERK	synthetic	[92]
**124d**	Anti-HeLa IC_50_ = 1.22 μM	Ras/Raf/MEK/ERK	synthetic	[92]
**125a**	Anti-HeLa IC_50_ = 1.18 μM	Ras/Raf/MEK/ERK	synthetic	[92]
**125b**	Anti-HeLa IC_50_ = 0.83 μM	Ras/Raf/MEK/ERK	synthetic	[92]
**125c**	Anti-HeLa IC_50_ = 0.99 μM	Ras/Raf/MEK/ERK	synthetic	[92]
**125d**	Anti-HeLa IC_50_ = 0.08 μM	Ras/Raf/MEK/ERK	synthetic	[92]
**125e**	Anti-HeLa IC_50_ > 50 μM	Ras/Raf/MEK/ERK	synthetic	[92]
**125f**	Anti-HeLa IC_50_ > 50 μM	Ras/Raf/MEK/ERK	synthetic	[92]
**125g**	Anti-HeLa IC_50_ > 50 μM	Ras/Raf/MEK/ERK	synthetic	[92]
**125h**	Anti-HeLa IC_50_ = 46.01 μM	Ras/Raf/MEK/ERK	synthetic	[92]
**126a**	Anti-MDA-MB-231 IC_50_ = 5.32 μM	Ras/Raf/MEK/ERK	synthetic	[92]
**126b**	Anti-MDA-MB-231 IC_50_ = 12.25 μM	Ras/Raf/MEK/ERK	synthetic	[92]
**126c**	Anti-MDA-MB-231 IC_50_ = 13.17 μM	Ras/Raf/MEK/ERK	synthetic	[92]
**126d**	Anti-HeLa IC_50_ = 4.28 μM	Ras/Raf/MEK/ERK	synthetic	[92]
**126e**	Anti-HeLa IC_50_ > 50 μM	Ras/Raf/MEK/ERK	synthetic	[92]
**126f**	Anti-HeLa IC_50_ = 30.94 μM	Ras/Raf/MEK/ERK	synthetic	[92]
**126g**	Anti-HeLa IC_50_ > 50 μM	Ras/Raf/MEK/ERK	synthetic	[92]
**126h**	Anti-HeLa IC_50_ > 50 μM	Ras/Raf/MEK/ERK	synthetic	[92]
**127a**	Anti-MDA-MB-231 IC_50_ = 18.75 μM	Ras/Raf/MEK/ERK	synthetic	[92]
**127b**	Anti-HeLa IC_50_ = 12.82 μM	Ras/Raf/MEK/ERK	synthetic	[92]
**127c**	Anti-MDA-MB-231 IC_50_ = 31.57 μM	Ras/Raf/MEK/ERK	synthetic	[92]
**127d**	Anti-HeLa IC_50_ = 10.92 μM	Ras/Raf/MEK/ERK	synthetic	[92]
**127e**	Anti-HeLa IC_50_ > 50 μM	Ras/Raf/MEK/ERK	synthetic	[92]
**127f**	Anti-HeLa IC_50_ > 50 μM	Ras/Raf/MEK/ERK	synthetic	[92]
**127g**	Anti-HeLa IC_50_ > 50 μM	Ras/Raf/MEK/ERK	synthetic	[92]
**127h**	Anti-HeLa IC_50_ > 50 μM	Ras/Raf/MEK/ERK	synthetic	[92]
**128a**	Anti-A549 IC_50_ = 15.21 μM	-	synthetic	[93]
**128b**	Anti-A549 IC_50_ = 12.65 μM	-	synthetic	[93]
**128c**	Anti-A549 IC_50_ = 13.34 μM	-	synthetic	[93]
**128d**	Anti-A549 IC_50_ = 12.23 μM	-	synthetic	[93]
**128e**	Anti-A549 IC_50_ = 8.34 μM	-	synthetic	[93]
**128f**	Anti-A549 IC_50_ = 5.34 μM	-	synthetic	[93]
**128g**	Anti-H1975 IC_50_ = 12.95 μM	-	synthetic	[93]
**128h**	Anti-H1975 IC_50_ = 14.31 μM	-	synthetic	[93]
**128i**	Anti-A549 IC_50_ = 10.28 μM	-	synthetic	[93]
**128j**	Anti-A549 IC_50_ = 9.01 μM	-	synthetic	[93]
**128k**	Anti-A549 IC_50_ = 23.91 μM	-	synthetic	[93]
**128l**	Anti-A431 IC_50_ = 12.56 μM	-	synthetic	[93]
**129a**	Anti-K562 IC_50_ = 0.850 μM	Tubulin	synthetic	[94]
**129b**	Anti-K562 IC_50_ = 0.011 μM	Tubulin	synthetic	[94]
**129c**	Anti-K562 IC_50_ = 0.127 μM	Tubulin	synthetic	[94]
**129d**	Anti-K562 IC_50_ = 0.009 μM	Tubulin	synthetic	[94]
**129e**	Anti-K562 IC_50_ = 0.108 μM	Tubulin	synthetic	[94]
**129f**	Anti-K562 IC_50_ = 1.055 μM	Tubulin	synthetic	[94]
**129g**	Anti-K562 IC_50_ = 0.069 μM	Tubulin	synthetic	[94]
**129h**	Anti-K562 IC_50_ = 0.563 μM	Tubulin	synthetic	[94]
**129i**	Anti-K562 IC_50_ > 1 μM	Tubulin	synthetic	[94]
**129j**	Anti-K562 IC_50_ > 1 μM	Tubulin	synthetic	[94]
**129k**	Anti-K562 IC_50_ > 1 μM	Tubulin	synthetic	[94]
**129l**	Anti-K562 IC_50_ = 0.346 μM	Tubulin	synthetic	[94]
**129m**	Anti-K562 IC_50_ = 0.074 μM	Tubulin	synthetic	[94]
**129n**	Anti-K562 IC_50_ > 1 μM	Tubulin	synthetic	[94]
**129o**	Anti-K562 IC_50_ > 1 μM	Tubulin	synthetic	[94]
**130a**	Anti-K562 IC_50_ = 0.040 μM	Tubulin	synthetic	[94]
**130b**	Anti-K562 IC_50_ = 0.026 μM	Tubulin	synthetic	[94]
**130c**	Anti-K562 IC_50_ = 0.015 μM	Tubulin	synthetic	[94]
**130d**	Anti-K562 IC_50_ = 1.239 μM	Tubulin	synthetic	[94]
**131**	Anti-HCT116 IC_50_ = 3.31 μM Anti-U87-MG IC_50_ = 1.47 μM Anti-HepG2 IC_50_ = 3.66 μM Anti-BGC823 IC_50_ = 1.77 μM Anti-PC9 IC_50_ = 3.12 μM	NF-*κ*B	synthetic	[95]
**132**	Anti-HepG2 IC_50_ = 14.3 μM	-	synthetic	[96]
**133**	Anti-HepG2 IC_50_ = 9.2 μM	-	synthetic	[96]
**134a**	Anti-HepG2 IC_50_ = 14.3 μM	Hsp90^N^	synthetic	[97]
**134b**	Anti-HepG2 IC_50_ = 9.2 μM	Hsp90^N^	synthetic	[97]
**134c**	Anti-HepG2 IC_50_ = 10.9 μM	Hsp90^N^	synthetic	[97]
**134d**	Anti-HepG2 IC_50_ = 13.1 μM	Hsp90^N^	synthetic	[97]
**134e**	Anti-HepG2 IC_50_ = 6.4 μM	Hsp90^N^	synthetic	[97]
**134f**	Anti-Hela IC_50_ = 6.9 μM	Hsp90^N^	synthetic	[97]
**134g**	Anti-HepG2 IC_50_ = 16.1 μM	Hsp90^N^	synthetic	[97]
**135**	Anti-HCT-116 IC_50_ = 0.16 μM	HER1, HER2, proteasome, and hTS appear as promising targets for these compounds	synthetic	[98]
**136a**	Anti-HCT-116 IC_50_ = 1.55 μM	HER1, HER2, proteasome, and hTS appear as promising targets for these compounds	synthetic	[98]
**136b**	Anti-RPMI-8226 IC_50_ = 1.29 μM	HER1, HER2, proteasome, and hTS appear as promising targets for these compounds	synthetic	[98]
**136c**	Anti-MDA-MB-435 IC_50_ = 0.30 μM	HER1, HER2, proteasome, and hTS appear as promising targets for these compounds	synthetic	[98]
**136d**	Anti-HCT-116 IC_50_ = 0.26 μM	HER1, HER2, proteasome, and hTS appear as promising targets for these compounds	synthetic	[98]
**136e**	-	HER1, HER2, proteasome, and hTS appear as promising targets for these compounds	synthetic	[98]
**136f**	-	HER1, HER2, proteasome, and hTS appear as promising targets for these compounds	synthetic	[98]
**136g**	-	HER1, HER2, proteasome, and hTS appear as promising targets for these compounds	synthetic	[98]
**137a**	Anti-A549 IC_50_ = 6.1 μM	EGFR, BRAF^V600E^	synthetic	[99]
**137b**	Anti-Panc-1 IC_50_ = 2.9 μM	EGFR, BRAF^V600E^	synthetic	[99]
**138a**	Anti-MCF-7 IC_50_ = 23.1 μM	EGFR, BRAF^V600E^	synthetic	[99]
**138b**	Anti-MCF-7 IC_50_ = 29.5 μM	EGFR, BRAF^V600E^	synthetic	[99]
**139a**	Anti-MCF-7 IC_50_ = 3.2 μM	EGFR, BRAF^V600E^	synthetic	[99]
**139b**	Anti-Panc-1 IC_50_ = 4.5 μM	EGFR, BRAF^V600E^	synthetic	[99]
**140**	Anti-HCT116 IC_50_ = 2.11 μM Anti-U87MG IC_50_ = 2.47 μM Anti-HepG2 IC_50_ = 4.71 μM Anti-BGC823 IC_50_ = 5.23 μM Anti-PC9 IC_50_ = 3.00 μM	NF-*κ*B, STAT3	synthetic	[100]
**141a**	Anti-A549 IC_50_ = 19.24 μM Anti-NCI-H460 IC_50_ = 24.61 μM		synthetic	[101]
**141b**	Anti-A549 IC_50_ = 18.21 μM	STAT3	synthetic	[101]
**141c**	-	STAT3	synthetic	[101]
**142a**	Anti-A549 IC_50_ = 13.78 μM Anti-NCI-H460 IC_50_ = 17.13 μM	STAT3	synthetic	[101]
**142b**	Anti-A549 IC_50_ = 14.34 μM Anti-NCI-H460 IC_50_ = 18.32 μM	STAT3	synthetic	[101]
**142c**	Anti-A549 IC_50_ = 12.94 μM	STAT3	synthetic	[101]
**142d**	Anti-A549 IC_50_ = 13.05 μM	STAT3	synthetic	[101]
**143**	Anti-HIF-1a IC_50_ = 0.05 μM	HIF-1a	synthetic	[102]
**144**	Anti-CYP1A1 IC_50_ = 117.6 μM Anti-CYP1B1 IC_50_ = 1.0 μM Anti-CYP1A2 IC_50_ > 1000 μM	CYP1B1	synthetic	[103]
**145a**	Anti-MGC-803 IC_50_ = 1.86 μM	Caspase3/9, cleaved-PARP	synthetic	[104]
**145b**	Anti-MGC-803 IC_50_ = 2.39 μM	Caspase3/9, cleaved-PARP	synthetic	[104]
**145c**	Anti-MGC-803 IC_50_ = 2.63 μM	Caspase3/9, cleaved-PARP	synthetic	[104]
**145d**	Anti-MGC-803 IC_50_ = 1.62 μM	Caspase3/9, cleaved-PARP	synthetic	[104]
**145e**	Anti-MGC-803 IC_50_ = 1.38 μM	Caspase3/9, cleaved-PARP	synthetic	[104]
**145f**	Anti-MGC-803 IC_50_ = 3.24 μM	Caspase3/9, cleaved-PARP	synthetic	[104]
**145g**	Anti-MGC-803 IC_50_ = 3.82 μM	Caspase3/9, cleaved-PARP	synthetic	[104]
**145h**	Anti-MGC-803 IC_50_ = 2.28 μM	Caspase3/9, cleaved-PARP	synthetic	[104]
**145i**	Anti-MGC-803 IC_50_ = 4.25 μM	Caspase3/9, cleaved-PARP	synthetic	[104]
**145j**	Anti-MGC-803 IC_50_ = 3.22 μM	Caspase3/9, cleaved-PARP	synthetic	[104]
**146a**	Anti-MGC-803 IC_50_ = 8.26 μM	Caspase3/9, cleaved-PARP	synthetic	[104]
**146b**	Anti-MGC-803 IC_50_ = 4.63 μM	Caspase3/9, cleaved-PARP	synthetic	[104]
**146c**	Anti-MGC-803 IC_50_ = 4.54 μM	Caspase3/9, cleaved-PARP	synthetic	[104]
**146d**	Anti-MGC-803 IC_50_ = 8.25 μM	Caspase3/9, cleaved-PARP	synthetic	[104]
**146e**	Anti-MGC-803 IC_50_ = 3.73 μM	Caspase3/9, cleaved-PARP	synthetic	[104]
**146f**	Anti-MGC-803 IC_50_ = 10.21 μM	Caspase3/9, cleaved-PARP	synthetic	[104]
**147**	10 μM Anti-MCF-7 Inhibition = 44.76% Anti-A549 Inhibition = 44.26%	Topoisomerase II	synthetic	[105]
**148a**	Anti-EAC IC_50_ = 62.25 μM	Vascular Endothelial Growth Factor (VEGF)	synthetic	[106]
**148b**	Anti-A375 IC_50_ = 69.52 μM	Vascular Endothelial Growth Factor (VEGF)	synthetic	[106]
**148c**	Anti-ACHN IC_50_ = 62.65 μM	Vascular Endothelial Growth Factor (VEGF)	synthetic	[106]
**149**	Anti-MOLT-4 EC_50_ = 33.4 μM	-	synthetic	[107]
**150**	Anti-RPMI-8226 EC_50_ = 23.3 μM	-	synthetic	[107]
**151**	Anti-MOLT-4 EC_50_ = 33.5 μM	-	synthetic	[107]
**152**	Anti-RPMI-8226 EC_50_ = 16.3 μM	-	synthetic	[107]
**153**	Anti-MOLT-4 EC_50_ = 24.4 μM	-	synthetic	[107]

Summary: Among the recognized anticancer drugs, such as camptothecin and cabozantinib, quinoline scaffolds are contained. Therefore, quinoline has been widely studied and is considered to be an efficient chemical structure in antitumor research. At present, among all the biological activities related to quinoline, anticancer activity has been reported the most. Anticancer drugs containing quinoline structure are divided into the following three main structural categories: non-fused quinoline (such as cabozantinib); fusion quinoline compounds (such as camptothecin); metal complexes with quinoline or phenanthroline ligands.

The compounds containing quinoline structure antitumor activity are mainly the first two, and most of them can show strong antitumor activity. For example, for HeLa cells, **105a** and **105b** had the strongest activity (IC_50_ values were 0.37 and 0.36 μM, respectively). For MCF-7 cells, **120** had the strongest activity (IC_50_ values were 0.60 μM). For A549 cells, **121** had the strongest activity (IC_50_ values were 2.3 μM). In addition, the mechanism of action is mainly aimed at the mechanism of cell division, or the induction of apoptosis. However, the research on the mechanism of action is not deep enough and there is still a lack of in vivo research.

## 4. Conclusions

Natural products have always been a rich source of effective drugs and will continue to be an important source of new pharmacological lead drugs. However, natural physiologically active chemicals may have adverse pharmacological properties that limit their use, such as cytotoxicity, excessive lipophilicity, or poor oral absorption. Another major obstacle to the use of natural products in drug research is the inability to obtain these derivatives from sustainable sources. The quinoline ring has many medicinal values, and it is becoming more and more popular as a multifunctional drug chemical scaffold. Due to the wide range of biological functions of quinoline molecules, natural compounds with structural changes are often used as quinoline molecules and developed into key heterocyclic scaffolds to help discover new drugs with new structures and new processes. We have been looking for new natural product quinoline derivatives that can produce potential biological activity. This literature review shows that quinoline scaffolds have considerable biological relevance in anti-osteoporosis, anti-virus, anti-diabetes, anti-inflammation, anti-thrombosis, anti-parasitic, antimalarial, antibacterial, and anticancer studies, which leads to the emergence of many efficient quinoline compounds in many therapeutic fields. Among them, anti-malaria and antitumor are the two most popular research fields. Observing quinoline-based antimalarial drugs, it can be seen that most of them still have traditional pharmacodynamic units, which also exist in quinoline antimalarial drugs, such as chloroquine, amodiaquine, and primaquine. In cancer research, there are many types of tumors, and the occurrence and progression of tumors are also complex. There are many cancer-related targets, and the chemical drug space exploration around quinoline antitumor drugs has great diversity, and it is easier to develop antitumor drugs with strong activity and small side effects. In short, in the future research of medicinal chemistry, quinoline drugs will continue to be used as superior scaffolds for the development of derivatives with high biological activity, among which antimalarial and antitumor development are of the greatest value. This review provides a comprehensive data resource of natural product quinoline derivatives for pharmaceutical chemists engaged in drug design and development, which is helpful for pharmaceutical companies to carry out richer and more organized drug discovery actions in experimental research so that the scientific community can reasonably design and develop various optimized, new, and targeted quinoline derivatives.

## Abbreviations

3D7strain	Plasmodium falciparum
ACHE	Acetylcholinesterase
ACI	Coccidiosis inhibition rate of Eimeria tenella
ALP	Alkaline phosphatase
ALT	Alanine aminotransferase
AST	Aspartate aminotransferase
BCHE	Cholinesterase
BMP-2	Bone morphogenetic protein-2
DMSO	Dimethyl sulfoxide
FMR	Insecticidal inhibition rate
FRD	Fumarate reductase
FXa	Activation of X factor
HDL-C	High-density lipoprotein cholesterol
IL-6	Interleukin-6
KM mice	Kunming mice
LPS	Lipopolysaccharides
MR	The mite inhibition rate
OA	Oleanolic acid
OCLs	Osteoclast-like multinucleated cells
OCN	Osteoblast secretory protein
PC-3 cells	Human prostate cancer cell line
RUNX-2	Runt-related transcription factor-2
SNB-19	Human glioma adherent cell line
THP1 cells	Human monocytic leukemia
U-937 cells	Cell line exhibiting monocyte morphology

## References

[1] Newman D.J., Cragg G.M. (2020). Natural Products as Sources of New Drugs over the Nearly Four Decades from January 1981 to September 2019. J. Nat. Prod..

[2] Patel R.V., Park S.W. (2014). Access to a new class of biologically active quinoline based 1,2,4-triazoles. Eur. J. Med. Chem..

[3] Narwal S., Kumar S., Verma P.K. (2016). Synthesis and therapeutic potential of quinoline derivatives. Res. Chem. Inter..

[4] Feng L., Lv K., Liu M., Wang S., Zhao J., You X., Li S., Cao J., Guo H. (2012). Synthesis and in vitro antibacterial activity of gemifloxacin derivatives containing a substituted benzyloxime moiety. Eur. J. Med. Chem..

[5] Musiol R., Serda M., Hensel-Bielowka S., Polanski J. (2010). Quinoline-Based Antifungals. Curr. Med. Chem..

[6] Medapi B., Renuka J., Saxena S., Sridevi J.P., Medishetti R., Kulkarni P., Yogeeswari P., Sriram D. (2015). Design and synthesis of novel quinoline-aminopiperidine hybrid analogues as *Mycobacterium tuberculosis* DNA gyraseB inhibitors. Bioorg. Med. Chem..

[7] Ma X., Zhou W., Brun R. (2009). Synthesis, in vitro antitrypanosomal and antibacterial activity of phenoxy, phenylthio or benzyloxy substituted quinolones. Bioorg. Med. Chem. Lett..

[8] Rossiter S., Peron J.M., Whitfield P.J., Jones K. (2005). Synthesis and anthelmintic properties of arylquinolines with activity against drug-resistant nematodes. Bioorg. Med. Chem. Lett..

[9] Sun J., Zhu H., Yang Z.M., Zhu H.L. (2013). Synthesis, molecular modeling and biological evaluation of 2-aminomethyl-5-(quinolin-2-yl)-1,3,4-oxadiazole-2(3H)-thione quinolone derivatives as novel anticancer agent. Eur. J. Med. Chem..

[10] Majerz-Maniecka K., Musiol R., Skorska-Stania A., Tabak D., Mazur P., Oleksyn B.J., Polanski J. (2011). X-ray and molecular modelling in fragment-based design of three small quinoline scaffolds for HIV integrase inhibitors. Bioorg. Med. Chem..

[11] Rano T.A., Sieber-McMaster E., Pelton P.D., Yang M., Demarest K.T., Kuo G.H. (2009). Design and synthesis of potent inhibitors of cholesteryl ester transfer protein (CETP) exploiting a 1,2,3,4-tetrahydroquinoline platform. Bioorg. Med. Chem. Lett..

[12] Roma G., Grossi G., Di Braccio M., Piras D., Ballabeni V., Tognolini M., Bertoni S., Barocelli E. (2008). 1,8-Naphthyridines VII. New substituted 5-amino[1,2,4]triazolo[4,3-a][1,8]naphthyridine-6-carboxamides and their isosteric analogues, exhibiting notable anti-inflammatory and/or analgesic activities, but no acute gastrolesivity. Eur. J. Med. Chem..

[13] Mantoani S.P., Chierrito T.P., Vilela A.F., Cardoso C.L., Martinez A., Carvalho I. (2016). Novel Triazole-Quinoline Derivatives as Selective Dual Binding Site Acetylcholinesterase Inhibitors. Molecules.

[14] Duarte Y., Fonseca A., Gutiérrez M., Adasme-Carreño F., Muñoz-Gutierrez C., Alzate-Morales J., Santana L., Uriarte E., Álvarez R., Matos M.J. (2019). Novel Coumarin-Quinoline Hybrids: Design of Multitarget Compounds for Alzheimer’s Disease. Chemistry.

[15] Wang X.Q., Xia C.L., Chen S.B., Tan J.H., Ou T.M., Huang S.L., Li D., Gu L.Q., Huang Z.S. (2015). Design, synthesis, and biological evaluation of 2-arylethenylquinoline derivatives as multifunctional agents for the treatment of Alzheimer’s disease. Eur. J. Med. Chem..

[16] Tintas M.L., Foucout L., Petit S., Oudeyer S., Gourand F., Barre L., Papamicael C., Levacher V. (2014). New developments in redox chemical delivery systems by means of 1,4-dihydroquinoline-based targetor: Application to galantamine delivery to the brain. Eur. J. Med. Chem..

[17] Benchekroun M., Pachon-Angona I., Luzet V., Martin H., Oset-Gasque M.J., Marco-Contelles J., Ismaili L. (2019). Synthesis, antioxidant and Abeta anti-aggregation properties of new ferulic, caffeic and lipoic acid derivatives obtained by the Ugi four-component reaction. Bioorg. Chem..

[18] Shah M.S., Najam-Ul-Haq M., Shah H.S., Farooq Rizvi S.U., Iqbal J. (2018). Quinoline containing chalcone derivatives as cholinesterase inhibitors and their in silico modeling studies. Comput. Biol. Chem..

[19] Li J.F., Zhao Y., Cai M.M., Li X.F., Li J.X. (2009). Synthesis and evaluation of a novel series of heterocyclic oleanolic acid derivatives with anti-osteoclast formation activity. Eur. J. Med. Chem..

[20] Maurya S.W., Dev K., Singh K.B., Rai R., Siddiqui I.R., Singh D., Maurya R. (2017). Synthesis and biological evaluation of heterocyclic analogues of pregnenolone as novel anti-osteoporotic agents. Bioorg. Med. Chem. Lett..

[21] Li F., Lee E.M., Sun X., Wang D., Tang H., Zhou G.C. (2020). Design, synthesis and discovery of andrographolide derivatives against Zika virus infection. Eur. J. Med. Chem..

[22] Baltina L.A., Lai H.C., Liu Y.C., Huang S.H., Hour M.J., Baltina L.A., Nugumanov T.R., Borisevich S.S., Khalilov L.M., Petrova S.F. (2021). Glycyrrhetinic acid derivatives as Zika virus inhibitors: Synthesis and antiviral activity in vitro. Bioorg. Med. Chem..

[23] Wang L.J., Geng C.A., Ma Y.B., Huang X.Y., Luo J., Chen H., Zhang X.M., Chen J.J. (2012). Synthesis, biological evaluation and structure-activity relationships of glycyrrhetinic acid derivatives as novel anti-hepatitis B virus agents. Bioorg. Med. Chem. Lett..

[24] Papi Reddy K., Singh A.B., Puri A., Srivastava A.K., Narender T. (2009). Synthesis of novel triterpenoid (lupeol) derivatives and their in vivo antihyperglycemic and antidyslipidemic activity. Bioorg. Med. Chem. Lett..

[25] Nam K.Y., Damodar K., Jeon S.H., Lee J.T., Lee Y. (2021). Design and Synthesis of π-Extended Resveratrol Analogues and In Vitro Antioxidant and Anti-Inflammatory Activity Evaluation. Molecules.

[26] Bian M., Zhen D., Shen Q.K., Du H.H., Ma Q.Q., Quan Z.S. (2021). Structurally modified glycyrrhetinic acid derivatives as anti-inflammatory agents. Bioorg. Chem..

[27] Ma Q., Bian M., Gong G., Bai C., Liu C., Wei C., Quan Z.S., Du H.H. (2022). Synthesis and Evaluation of Bakuchiol Derivatives as Potent Anti-inflammatory Agents In Vitro and In Vivo. J. Nat. Prod..

[28] Chen P., Zhang D., Li M., Wu Q., Lam Y.P.Y., Guo Y., Chen C., Bai N., Malhotra S., Li W. (2019). Discovery of novel, potent, isosteviol-based antithrombotic agents. Eur. J. Med. Chem..

[29] Leverrier A., Bero J., Frederich M., Quetin-Leclercq J., Palermo J. (2013). Antiparasitic hybrids of Cinchona alkaloids and bile acids. Eur. J. Med. Chem..

[30] Nisha, Kumar K., Bhargava G., Land K.M., Chang K.H., Arora R., Sen S., Kumar V. (2014). N-Propargylated isatin-Mannich mono- and bis-adducts: Synthesis and preliminary analysis of in vitro activity against *Tritrichomonas foetus*. Eur. J. Med. Chem..

[31] Coa J.C., García E., Carda M., Agut R., Vélez I.D., Muñoz J.A., Yepes L.M., Robledo S.M., Cardona W.I. (2017). Synthesis, leishmanicidal, trypanocidal and cytotoxic activities of quinoline-chalcone and quinoline-chromone hybrids. Med. Chem. Res..

[32] Huang J., Lv M., Thapa S., Xu H. (2018). Synthesis of novel quinolinomatrine derivatives and their insecticidal/acaricidal activities. Bioorg. Med. Chem. Lett..

[33] Wang Z., Zhou L.-J., Wang Y.-I., Weng Y.-B., He J., Nie K. (2011). Synthesis and anticoccidial activities of substituted ethyl 4-hydroxy-11-oxo-11H-chromeno[2,3-g]quinoline-3-carboxylates. J. Chem. Res..

[34] Roussaki M., Hall B., Lima S.C., da Silva A.C., Wilkinson S., Detsi A. (2013). Synthesis and anti-parasitic activity of a novel quinolinone-chalcone series. Bioorg. Med. Chem. Lett..

[35] Pan L., Li X.-Z., Sun D.-A., Jin H., Guo H.-R., Qin B. (2016). Design and synthesis of novel coumarin analogs and their nematicidal activity against five phytonematodes. Chin. Chem. Lett..

[36] Guo Y., Yan Y., Yu X., Wang Y., Zhi X.Y., Hu Y., Xu H. (2012). Synthesis and insecticidal activity of some novel fraxinellone-based esters. J. Agric. Food Chem..

[37] Guo H.Y., Jin C., Zhang H.M., Jin C.M., Shen Q.K., Quan Z.S. (2019). Synthesis and Biological Evaluation of (+)-Usnic Acid Derivatives as Potential Anti-Toxoplasma gondii Agents. J. Agric. Food Chem..

[38] Deng H., Huang X., Jin C., Jin C.M., Quan Z.S. (2020). Synthesis, in vitro and in vivo biological evaluation of dihydroartemisinin derivatives with potential anti-Toxoplasma gondii agents. Bioorg. Chem..

[39] Lombard M.C., N’Da D.D., Breytenbach J.C., Kolesnikova N.I., Tran Van Ba C., Wein S., Norman J., Denti P., Vial H., Wiesner L. (2012). Antimalarial and anticancer activities of artemisinin-quinoline hybrid-dimers and pharmacokinetic properties in mice. Eur. J. Pharm. Sci..

[40] Raj R., Biot C., Carrere-Kremer S., Kremer L., Guerardel Y., Gut J., Rosenthal P.J., Forge D., Kumar V. (2014). 7-chloroquinoline-isatin conjugates: Antimalarial, antitubercular, and cytotoxic evaluation. Chem. Biol. Drug Des..

[41] Raj R., Singh P., Singh P., Gut J., Rosenthal P.J., Kumar V. (2013). Azide-alkyne cycloaddition en route to 1H-1,2,3-triazole-tethered 7-chloroquinoline-isatin chimeras: Synthesis and antimalarial evaluation. Eur. J. Med. Chem..

[42] Nisha, Gut J., Rosenthal P.J., Kumar V. (2014). beta-amino-alcohol tethered 4-aminoquinoline-isatin conjugates: Synthesis and antimalarial evaluation. Eur. J. Med. Chem..

[43] Videnovic M., Opsenica D.M., Burnett J.C., Gomba L., Nuss J.E., Selakovic Z., Konstantinovic J., Krstic M., Segan S., Zlatovic M. (2014). Second generation steroidal 4-aminoquinolines are potent, dual-target inhibitors of the botulinum neurotoxin serotype A metalloprotease and *P. falciparum* malaria. J. Med. Chem..

[44] Leverrier A., Bero J., Cabrera J., Frederich M., Quetin-Leclercq J., Palermo J.A. (2015). Structure-activity relationship of hybrids of Cinchona alkaloids and bile acids with in vitro antiplasmodial and antitrypanosomal activities. Eur. J. Med. Chem..

[45] Sharma B., Kaur S., Legac J., Rosenthal P.J., Kumar V. (2020). Synthesis, anti-plasmodial and cytotoxic evaluation of 1H-1,2,3-triazole/acyl hydrazide integrated tetrahydro-beta-carboline-4-aminoquinoline conjugates. Bioorg. Med. Chem. Lett..

[46] Vinindwa B., Dziwornu G.A., Masamba W. (2021). Synthesis and Evaluation of Chalcone-Quinoline Based Molecular Hybrids as Potential Anti-Malarial Agents. Molecules.

[47] Rodrigues T., Ressurreicao A.S., da Cruz F.P., Albuquerque I.S., Gut J., Carrasco M.P., Goncalves D., Guedes R.C., dos Santos D.J., Mota M.M. (2013). Flavones as isosteres of 4(1H)-quinolones: Discovery of ligand efficient and dual stage antimalarial lead compounds. Eur. J. Med. Chem..

[48] Paul N., Muthusubramanian S. (2013). Synthesis, antimicrobial, and cytotoxicity studies of novel sulfur-linked quinoline–coumarin bisheterocycles. Med. Chem. Res..

[49] Patel D., Brahmbhatt D.I. (2017). A Novel and Efficient Synthesis of Various 7-Hydroxy-9(Furo[2,3-b]Quinolin-2Yl)6H- Benzo[c]Coumarins and Evaluation of their Antimicrobial Activity. Int. J. Pharm. Res. Sch..

[50] Subhedar D.D., Shaikh M.H., Nawale L., Sarkar D., Khedkar V.M., Shingate B.B. (2017). Quinolidene based monocarbonyl curcumin analogues as promising antimycobacterial agents: Synthesis and molecular docking study. Bioorg. Med. Chem. Lett..

[51] Campanico A., Carrasco M.P., Njoroge M., Seldon R., Chibale K., Perdigao J., Portugal I., Warner D.F., Moreira R., Lopes F. (2019). Azaaurones as Potent Antimycobacterial Agents Active against MDR- and XDR-TB. ChemMedChem.

[52] Kumar G., Lathwal E., Saroha B., Kumar S., Kumar S., Chauhan N.S., Kumar T. (2020). Synthesis and Biological Evaluation of Quinoline-Based Novel Aurones. ChemistrySelect.

[53] Sabatini S., Gosetto F., Manfroni G., Tabarrini O., Kaatz G.W., Patel D., Cecchetti V. (2011). Evolution from a natural flavones nucleus to obtain 2-(4-Propoxyphenyl)quinoline derivatives as potent inhibitors of the *S. aureus* NorA efflux pump. J. Med. Chem..

[54] Wang W., Zhang S., Wang J., Wu F., Wang T., Xu G. (2021). Bioactivity-Guided Synthesis Accelerates the Discovery of 3-(Iso)quinolinyl-4-chromenones as Potent Fungicide Candidates. J. Agric. Food Chem..

[55] Gogoi S., Shekarrao K., Duarah A., Bora T.C., Gogoi S., Boruah R.C. (2012). A microwave promoted solvent-free approach to steroidal quinolines and their in vitro evaluation for antimicrobial activities. Steroids.

[56] Balaji G.L., Rajesh K., Priya R., Iniyavan P., Siva R., Vijayakumar V. (2012). Ultrasound-promoted synthesis, biological evaluation and molecular docking of novel 7-(2-chloroquinolin-4-yloxy)-4-methyl-2H-chromen-2-one derivatives. Med. Chem. Res..

[57] Khatkar A., Nanda A., Kumar P., Narasimhan B. (2013). Synthesis and antimicrobial evaluation of ferulic acid derivatives. Res. Chem. Intermed..

[58] Makula S.M.A. (2016). Design, Synthesis and Docking Study of Some Novel Isatin-Quinoline Hybrids as Potential Antitubercular Agents. Anti-Infect. Agents.

[59] Tabbi A., Tebbani D., Caporale A., Saturninob C., Nabavi S.F., Nabavic P.G., Arrac C., Cantürkd Z., Turan-Zitounie G., Merazigf H. (2017). New Adamantyl Chalcones: Synthesis, Antimicrobial and Anticancer Activities. Curr. Top. Med. Chem..

[60] Pan L., Li X., Jin H., Yang X., Qin B. (2017). Antifungal activity of umbelliferone derivatives: Synthesis and structure-activity relationships. Microb. Pathog..

[61] Kalt M.M., Schuehly W., Saf R., Ochensberger S., Solnier J., Bucar F., Kaiser M., Presser A. (2020). Palladium-catalysed synthesis of arylnaphthoquinones as antiprotozoal and antimycobacterial agents. Eur. J. Med. Chem..

[62] Li D.H., Hu P., Xu S.T., Fang C.Y., Tang S., Wang X.Y., Sun X.Y., Li H., Xu Y., Gu X.K. (2017). Lasiokaurin derivatives: Synthesis, antimicrobial and antitumor biological evaluation, and apoptosis-inducing effects. Arch. Pharm. Res..

[63] Abonia R., Insuasty D., Castillo J., Insuasty B., Quiroga J., Nogueras M., Cobo J. (2012). Synthesis of novel quinoline-2-one based chalcones of potential anti-tumor activity. Eur. J. Med. Chem..

[64] Kamal A., Suresh P., Ramaiah M.J., Mallareddy A., Imthiajali S., Pushpavalli S.N., Lavanya A., Pal-Bhadra M. (2012). Synthesis and biological evaluation of 4beta-sulphonamido and 4beta-[(4′-sulphonamido)benzamide]podophyllotoxins as DNA topoisomerase-IIalpha and apoptosis inducing agents. Bioorg. Med. Chem..

[65] Pudhom K., Nuanyai T., Matsubara K., Vilaivan T. (2012). Antiangiogenic activity of 3,4-seco-cycloartane triterpenes from Thai *Gardenia* spp. and their semi-synthetic analogs. Bioorg. Med. Chem. Lett..

[66] Kamal A., Mallareddy A., Suresh P., Lakshma Nayak V., Shetti R.V., Sankara Rao N., Tamboli J.R., Shaik T.B., Vishnuvardhan M.V., Ramakrishna S. (2012). Synthesis and anticancer activity of 4beta-alkylamidochalcone and 4beta-cinnamido linked podophyllotoxins as apoptotic inducing agents. Eur. J. Med. Chem..

[67] Zhao W., Chen L., Li H.M., Wang D.J., Li D.S., Chen T., Yuan Z.P., Tang Y.J. (2014). A rational design strategy of the novel topoisomerase II inhibitors for the synthesis of the 4-O-(2-pyrazinecarboxylic)-4′-demethylepipodophyllotoxin with antitumor activity by diminishing the relaxation reaction of topoisomerase II-DNA decatenation. Bioorg. Med. Chem..

[68] Ayan D., Maltais R., Hospital A., Poirier D. (2014). Chemical synthesis, cytotoxicity, selectivity and bioavailability of 5alpha-androstane-3alpha,17beta-diol derivatives. Bioorg. Med. Chem..

[69] Cui J., Liu L., Zhao D., Gan C., Huang X., Xiao Q., Qi B., Yang L., Huang Y. (2015). Synthesis, characterization and antitumor activities of some steroidal derivatives with side chain of 17-hydrazone aromatic heterocycle. Steroids.

[70] Jin X., Yan L., Li H.-J., Wang R.-L., Hu Z.-L., Jiang Y.-Y., Cao Y.-B., Yan T.-H., Sun Q.-Y. (2015). Novel Triazolyl Berberine Derivatives Prepared via CuAAC Click Chemistry: Synthesis, Anticancer Activity and Structure-Activity Relationships. Anti-Cancer Agents Med. Chem..

[71] Hayat F., Park S.H., Choi N.S., Lee J., Park S.J., Shin D. (2015). Synthesis and anticancer activity of 4-aza-daurinol derivatives. Arch. Pharm. Res..

[72] Srivastava V., Lee H. (2015). Synthesis and bio-evaluation of novel quinolino-stilbene derivatives as potential anticancer agents. Bioorg. Med. Chem..

[73] Raghavan S., Manogaran P., Gadepalli Narasimha K.K., Kalpattu Kuppusami B., Mariyappan P., Gopalakrishnan A., Venkatraman G. (2015). Synthesis and anticancer activity of novel curcumin-quinolone hybrids. Bioorg. Med. Chem. Lett..

[74] Cui J., Liu C., Liu L., Gan C., Lu Y., Chen S., Dong X., Huang Y. (2016). Synthesis and Antitumor Activities of Cholestane Derivatives with a Structure of 3-Hydroxy-6-hydrazone or 6-Carbonyl-3-hydrazone. Chin. J. Org. Chem..

[75] He D.M., Liu L., Zheng J.H., Yang C.H., Huang Y.M., Gan C.F., Cui J.G. (2016). Synthesis and antitumor activity of pyrazoline steroidal aromatic heterocyclic compounds. Chin. J. Med. Chem..

[76] Baji Á., Gyovai A., Wölfling J., Minorics R., Ocsovszki I., Zupkó I., Frank É. (2016). Microwave-assisted one-pot synthesis of steroid–quinoline hybrids and an evaluation of their antiproliferative activities on gynecological cancer cell lines. RSC Adv..

[77] Chaudhary V., Venghateri J.B., Dhaked H.P., Bhoyar A.S., Guchhait S.K., Panda D. (2016). Novel Combretastatin-2-aminoimidazole Analogues as Potent Tubulin Assembly Inhibitors: Exploration of Unique Pharmacophoric Impact of Bridging Skeleton and Aryl Moiety. J. Med. Chem..

[78] Sommerwerk S., Heller L., Kuhfs J., Csuk R. (2016). Selective killing of cancer cells with triterpenoic acid amides—The substantial role of an aromatic moiety alignment. Eur. J. Med. Chem..

[79] Shobeiri N., Rashedi M., Mosaffa F., Zarghi A., Ghandadi M., Ghasemi A., Ghodsi R. (2016). Synthesis and biological evaluation of quinoline analogues of flavones as potential anticancer agents and tubulin polymerization inhibitors. Eur. J. Med. Chem..

[80] Zhang L., Zhang Z., Chen F., Chen Y., Lin Y., Wang J. (2016). Aromatic heterocyclic esters of podophyllotoxin exert anti-MDR activity in human leukemia K562/ADR cells via ROS/MAPK signaling pathways. Eur. J. Med. Chem..

[81] Li Z., Su H., Yu W., Li X., Cheng H., Liu M., Pang X., Zou X. (2016). Design, synthesis and anticancer activities of novel otobain derivatives. Org. Biomol. Chem..

[82] Gu W., Jin X.Y., Li D.D., Wang S.F., Tao X.B., Chen H. (2017). Design, synthesis and in vitro anticancer activity of novel quinoline and oxadiazole derivatives of ursolic acid. Bioorg. Med. Chem. Lett..

[83] Gan C., Liu L., Cui J., Liu Z., Shi H., Lin Q., Sheng H., Yang C., Huang Y. (2017). Synthesis of Some Steroidal Derivatives with Side Chain of 20- and 22- Hydrazone Aromatic Heterocycles and their Antiproliferative Activity. Med. Chem..

[84] Yao G., Chen H., Chen L., Ge M., Yang J., Liu W., Xia M., Hayashi T., Guo C., Ikejima T. (2017). Autophagy promotes apoptosis induction through repressed nitric oxide generation in the treatment of human breast cancer MCF-7 cells with L-A03, a dihydroartemisinin derivative. Med. Chem. Res..

[85] Aly A.A., El-Sheref E.M., Bakheet M.E.M., Mourad M.A.E., Brase S., Ibrahim M.A.A., Nieger M., Garvalov B.K., Dalby K.N., Kaoud T.S. (2019). Design, synthesis and biological evaluation of fused naphthofuro[3,2-c] quinoline-6,7,12-triones and pyrano[3,2-c]quinoline-6,7,8,13-tetraones derivatives as ERK inhibitors with efficacy in BRAF-mutant melanoma. Bioorg. Chem..

[86] Sri Ramya P.V., Guntuku L., Angapelly S., Karri S., Digwal C.S., Babu B.N., Naidu V.G.M., Kamal A. (2018). Curcumin inspired 2-chloro/phenoxy quinoline analogues: Synthesis and biological evaluation as potential anticancer agents. Bioorg. Med. Chem. Lett..

[87] Taheri S., Nazifi M., Mansourian M., Hosseinzadeh L., Shokoohinia Y. (2019). Ugi efficient synthesis, biological evaluation and molecular docking of coumarin-quinoline hybrids as apoptotic agents through mitochondria-related pathways. Bioorg. Chem..

[88] Lipeeva A.V., Zakharov D.O., Gatilov Y.V., Pokrovskii M.A., Pokrovskii A.G., Shults E.E. (2019). Design and Synthesis of 3-(N-Substituted)aminocoumarins as Anticancer Agents from 3-Bromopeuruthenicin. ChemistrySelect.

[89] Shen Q.K., Deng H., Wang S.B., Tian Y.S., Quan Z.S. (2019). Synthesis, and evaluation of in vitro and in vivo anticancer activity of 14-substituted oridonin analogs: A novel and potent cell cycle arrest and apoptosis inducer through the p53-MDM2 pathway. Eur. J. Med. Chem..

[90] Zhao W., He L., Xiang T.L., Tang Y.J. (2019). Discover 4beta-NH-(6-aminoindole)-4-desoxy-podophyllotoxin with nanomolar-potency antitumor activity by improving the tubulin binding affinity on the basis of a potential binding site nearby colchicine domain. Eur. J. Med. Chem..

[91] Prashanth T., Avin B.R.V., Thirusangu P., Ranganatha V.L., Prabhakar B.T., Sharath Chandra J.N.N., Khanum S.A. (2019). Synthesis of coumarin analogs appended with quinoline and thiazole moiety and their apoptogenic role against murine ascitic carcinoma. Biomed. Pharmacother..

[92] Jin X.Y., Chen H., Li D.D., Li A.L., Wang W.Y., Gu W. (2019). Design, synthesis, and anticancer evaluation of novel quinoline derivatives of ursolic acid with hydrazide, oxadiazole, and thiadiazole moieties as potent MEK inhibitors. J. Enzyme Inhib. Med. Chem..

[93] Yang Y.T., Du S., Wang S., Jia X., Wang X., Zhang X. (2019). Synthesis of new steroidal quinolines with antitumor properties. Steroids.

[94] Li W., Xu F., Shuai W., Sun H., Yao H., Ma C., Xu S., Yao H., Zhu Z., Yang D.H. (2019). Discovery of Novel Quinoline-Chalcone Derivatives as Potent Antitumor Agents with Microtubule Polymerization Inhibitory Activity. J. Med. Chem..

[95] Jia X., Liu Q., Wang S., Zeng B., Du G., Zhang C., Li Y. (2020). Synthesis, cytotoxicity, and in vivo antitumor activity study of parthenolide semicarbazones and thiosemicarbazones. Bioorg. Med. Chem..

[96] Hoenke S., Heise N.V., Kahnt M., Hans-Peter D., Rene C. (2020). Betulinic acid derived amides are highly cytotoxic, apoptotic and selective. Eur. J. Med. Chem..

[97] Xu Y., Jing D., Zhao D., Wu Y., Lu X., Haroon U.R., Wang H., Wang L., Cao H. (2020). New modification strategy of matrine as Hsp90 inhibitors based on its specific L conformation for cancer treatment. Bioorg. Med. Chem..

[98] Insuasty D., García S., Abonia R., Insuasty B., Quiroga J., Nogueras M., Cobo J., Borosky G.L., Laali K.K. (2021). Design, synthesis, and molecular docking study of novel quinoline-based bis-chalcones as potential antitumor agents. Arch. Der Pharm..

[99] Mohassab A.M., Hassan H.A., Abdelhamid D., Gouda A.M., Youssif B.G., Tateishi H., Fujita M., Otsuka M., Abdel-Aziz M. (2021). Design and synthesis of novel quinoline/chalcone/1,2,4-triazole hybrids as potent antiproliferative agent targeting EGFR and BRAFV600E kinases. Bioorg. Chem..

[100] Zeng B., Cheng Y., Zheng K., Liu S., Shen L., Hu J., Li Y., Pan X. (2021). Design, synthesis and in vivo anticancer activity of novel parthenolide and micheliolide derivatives as NF-κB and STAT3 inhibitors. Bioorg. Chem..

[101] Song J.-R., Li N., Li D.-P. (2021). Synthesis and anti-proliferation activity of mogrol derivatives bearing quinoline and triazole moieties. Bioorg. Med. Chem. Lett..

[102] Shang F.-F., Wang J.Y., Xu Q., Deng H., Guo H.-Y., Jin X., Li X., Shen Q.-K., Quan Z.-S. (2021). Design, synthesis of novel celastrol derivatives and study on their antitumor growth through HIF-1α pathway. Eur. J. Med. Chem..

[103] Dong J., Huang G., Cui Q., Meng Q., Li S., Cui J. (2021). Discovery of heterocycle-containing α-naphthoflavone derivatives as water-soluble, highly potent and selective CYP1B1 inhibitors. Eur. J. Med. Chem..

[104] Guan Y., Liu X., Yuan X., Liu W., Li Y., Yu G., Tian X., Zhang Y., Song J., Li W. (2021). Design, Synthesis, and Anticancer Activity Studies of Novel Quinoline-Chalcone Derivatives. Molecules.

[105] Thorat N.M., Sarkate A.P., Lokwani D.K., Tiwari S.V., Azad R., Thopate S.R. (2021). N-Benzylation of 6-aminoflavone by reductive amination and efficient access to some novel anticancer agents via topoisomerase II inhibition. Mol. Divers..

[106] Jyothi M., Sherapura A., Khamees H.A., Prabhakar B.T., Khanum S.A. (2022). Synthesis, structure analysis, DFT calculations and energy frameworks of new coumarin appended oxadiazoles, to regress ascites malignancy by targeting VEGF mediated angiogenesis. J. Mol. Struct..

[107] Herrmann L., Yaremenko I.A., Capci A., Struwe J., Tailor D., Dheeraj A., Hodek J., Belyakova Y.Y., Radulov P.S., Weber J. (2022). Synthesis and in vitro Study of Artemisinin/Synthetic Peroxide-Based Hybrid Compounds against SARS-CoV-2 and Cancer. ChemMedChem.

